# Ventricular fibrillation dynamics reveal regional asymmetry in resilience to cardiac arrest and predict clinical outcome

**DOI:** 10.1093/cvr/cvag101

**Published:** 2026-05-28

**Authors:** Andrés Redondo-Rodríguez, Jorge G Quintanilla, Álvaro Macías, Peter Lee, David Calvo, Marinela Couselo-Seijas, Manuel Marina-Breysse, Ana Simón-Chica, Alba García-Escolano, Alba Ramos-Prada, José Manuel Alfonso-Almazán, Jesús Diz-Díaz, Laura Gil-Martínez, Sergio Muñoz-Romero, Carlos Galán-Arriola, Javier Sánchez-González, Juan José González-Ferrer, Victoria Cañadas-Godoy, Ricardo Salgado-Aranda, Alba Cruz-Galbán, María Jesús García-Torrent, José Luis Rojo-Álvarez, Javier Saiz, Borja Ibañez, Julián Pérez-Villacastín, Nicasio Pérez-Castellano, José Jalife, José M Ferrero, David Filgueiras-Rama

**Affiliations:** Centro Nacional de Investigaciones Cardiovasculares (CNIC), Advanced Development in Arrhythmia Mechanisms and Therapy Laboratory, Novel Arrhythmogenic Mechanisms Program, Madrid, Spain; Centro de Investigación Biomédica en Red de Enfermedades Cardiovasculares (CIBERCV), Madrid, Spain; Centro Nacional de Investigaciones Cardiovasculares (CNIC), Advanced Development in Arrhythmia Mechanisms and Therapy Laboratory, Novel Arrhythmogenic Mechanisms Program, Madrid, Spain; Centro de Investigación Biomédica en Red de Enfermedades Cardiovasculares (CIBERCV), Madrid, Spain; Instituto de Investigación Sanitaria del Hospital Clínico San Carlos (IdISSC), Cardiovascular Institute, Madrid, Spain; Centro Nacional de Investigaciones Cardiovasculares (CNIC), Cardiac Arrhythmia Laboratory, Cardiovascular Regeneration Program, Madrid, Spain; Essel Research and Development Inc., Toronto, Canada; Centro de Investigación Biomédica en Red de Enfermedades Cardiovasculares (CIBERCV), Madrid, Spain; Instituto de Investigación Sanitaria del Hospital Clínico San Carlos (IdISSC), Cardiovascular Institute, Madrid, Spain; Centro Nacional de Investigaciones Cardiovasculares (CNIC), Advanced Development in Arrhythmia Mechanisms and Therapy Laboratory, Novel Arrhythmogenic Mechanisms Program, Madrid, Spain; Centro de Investigación Biomédica en Red de Enfermedades Cardiovasculares (CIBERCV), Madrid, Spain; Centro Nacional de Investigaciones Cardiovasculares (CNIC), Advanced Development in Arrhythmia Mechanisms and Therapy Laboratory, Novel Arrhythmogenic Mechanisms Program, Madrid, Spain; Centro de Investigación Biomédica en Red de Enfermedades Cardiovasculares (CIBERCV), Madrid, Spain; Centro Nacional de Investigaciones Cardiovasculares (CNIC), Advanced Development in Arrhythmia Mechanisms and Therapy Laboratory, Novel Arrhythmogenic Mechanisms Program, Madrid, Spain; Centro Nacional de Investigaciones Cardiovasculares (CNIC), Advanced Development in Arrhythmia Mechanisms and Therapy Laboratory, Novel Arrhythmogenic Mechanisms Program, Madrid, Spain; Centro Nacional de Investigaciones Cardiovasculares (CNIC), Advanced Development in Arrhythmia Mechanisms and Therapy Laboratory, Novel Arrhythmogenic Mechanisms Program, Madrid, Spain; Fundación Interhospitalaria Para la Investigación Cardiovascular, Madrid, Spain; Centro Nacional de Investigaciones Cardiovasculares (CNIC), Advanced Development in Arrhythmia Mechanisms and Therapy Laboratory, Novel Arrhythmogenic Mechanisms Program, Madrid, Spain; Centro Nacional de Investigaciones Cardiovasculares (CNIC), Advanced Development in Arrhythmia Mechanisms and Therapy Laboratory, Novel Arrhythmogenic Mechanisms Program, Madrid, Spain; Cardiology Department, Hospital Universitario La Paz, Madrid, Spain; Cardiology Department, Instituto de Investigación Sanitaria Fundación Jiménez Díaz, Madrid, Spain; Centro de Investigación e Innovación en Bioingeniería (Ci2B), Universitat Politècnica de València, Valencia, Spain; Departamento de Teoría de la Señal y Comunicaciones y Sistemas Telemáticos y Computación, Universidad Rey Juan Carlos, Madrid, Spain; Centro de Investigación Biomédica en Red de Enfermedades Cardiovasculares (CIBERCV), Madrid, Spain; Centro Nacional de Investigaciones Cardiovasculares (CNIC), Translational Laboratory for Cardiovascular Imaging and Therapy, Myocardial Homeostasis & Cardiac Injury Program, Madrid, Spain; Clinical Science, Philips Healthcare Iberia, Madrid, Spain; Centro de Investigación Biomédica en Red de Enfermedades Cardiovasculares (CIBERCV), Madrid, Spain; Instituto de Investigación Sanitaria del Hospital Clínico San Carlos (IdISSC), Cardiovascular Institute, Madrid, Spain; Centro de Investigación Biomédica en Red de Enfermedades Cardiovasculares (CIBERCV), Madrid, Spain; Instituto de Investigación Sanitaria del Hospital Clínico San Carlos (IdISSC), Cardiovascular Institute, Madrid, Spain; Instituto de Investigación Sanitaria del Hospital Clínico San Carlos (IdISSC), Cardiovascular Institute, Madrid, Spain; Instituto de Investigación Sanitaria del Hospital Clínico San Carlos (IdISSC), Cardiovascular Institute, Madrid, Spain; Centro de Investigación Biomédica en Red de Enfermedades Cardiovasculares (CIBERCV), Madrid, Spain; Instituto de Investigación Sanitaria del Hospital Clínico San Carlos (IdISSC), Cardiovascular Institute, Madrid, Spain; Departamento de Medicina, Universidad Complutense de Madrid, Madrid, Spain; Departamento de Teoría de la Señal y Comunicaciones y Sistemas Telemáticos y Computación, Universidad Rey Juan Carlos, Madrid, Spain; Centro de Investigación e Innovación en Bioingeniería (Ci2B), Universitat Politècnica de València, Valencia, Spain; Centro de Investigación Biomédica en Red de Enfermedades Cardiovasculares (CIBERCV), Madrid, Spain; Cardiology Department, Instituto de Investigación Sanitaria Fundación Jiménez Díaz, Madrid, Spain; Centro Nacional de Investigaciones Cardiovasculares (CNIC), Translational Laboratory for Cardiovascular Imaging and Therapy, Myocardial Homeostasis & Cardiac Injury Program, Madrid, Spain; Centro de Investigación Biomédica en Red de Enfermedades Cardiovasculares (CIBERCV), Madrid, Spain; Instituto de Investigación Sanitaria del Hospital Clínico San Carlos (IdISSC), Cardiovascular Institute, Madrid, Spain; Fundación Interhospitalaria Para la Investigación Cardiovascular, Madrid, Spain; Departamento de Medicina, Universidad Complutense de Madrid, Madrid, Spain; Centro de Investigación Biomédica en Red de Enfermedades Cardiovasculares (CIBERCV), Madrid, Spain; Instituto de Investigación Sanitaria del Hospital Clínico San Carlos (IdISSC), Cardiovascular Institute, Madrid, Spain; Fundación Interhospitalaria Para la Investigación Cardiovascular, Madrid, Spain; Departamento de Medicina, Universidad Complutense de Madrid, Madrid, Spain; Centro de Investigación Biomédica en Red de Enfermedades Cardiovasculares (CIBERCV), Madrid, Spain; Centro Nacional de Investigaciones Cardiovasculares (CNIC), Cardiac Arrhythmia Laboratory, Cardiovascular Regeneration Program, Madrid, Spain; Centro de Investigación e Innovación en Bioingeniería (Ci2B), Universitat Politècnica de València, Valencia, Spain; Centro Nacional de Investigaciones Cardiovasculares (CNIC), Advanced Development in Arrhythmia Mechanisms and Therapy Laboratory, Novel Arrhythmogenic Mechanisms Program, Madrid, Spain; Centro de Investigación Biomédica en Red de Enfermedades Cardiovasculares (CIBERCV), Madrid, Spain; Instituto de Investigación Sanitaria del Hospital Clínico San Carlos (IdISSC), Cardiovascular Institute, Madrid, Spain

**Keywords:** Ventricular fibrillation, Global ischemia, Cardiac arrest, Myocardial infarction

## Abstract

**Aims:**

Ventricular fibrillation (VF) is the leading cause of sudden cardiac death. Nevertheless, the mechanisms underlying VF dynamics in structurally normal hearts and infarct-related substrates remain incompletely understood. We aimed to characterize the electrophysiological, structural and molecular properties that explain VF dynamics during early phases (seconds) and long-duration episodes (minutes) in experimental pig models. Additionally, we assessed the clinical impact of VF dynamics on neurological prognosis following resuscitated cardiac arrest.

**Methods and results:**

A total of 72 pigs were included in the study. *In vivo* multipolar-catheter recordings revealed that early VF dynamics are independent of the infarct-related substrate. Healthy and infarcted animals consistently showed higher activation rates (AR) in the right ventricle (RV) than in the left ventricle (LV), and this gradient further increased during long-duration VF. *Ex vivo* panoramic optical mapping of long-duration VF episodes resembling cardiac arrest conditions confirmed *in vivo* findings and revealed earlier electrical depression in the LV. AR gradients during VF were primarily driven by an asymmetric response to the global ischaemia conditions intrinsically associated with VF, during which the RV exhibited greater resilience than the LV. Computational simulations, incorporating experimentally derived electrophysiological properties and region-specific metabolic parameters during ischaemia, demonstrated that the fibrillating RV preserves higher excitability than the LV, creating RV-to-LV AR gradients during VF. The translational relevance of these findings was evaluated in patients with VF events, in whom long-term (>2 min) ECG-derived AR patterns (*N* = 3) resembled those documented in pigs. Moreover, in 60 patients with out-of-hospital cardiac arrest, ECG-derived ARs prior to the first defibrillation shock for VF were associated with favourable neurological outcome.

**Conclusion:**

VF dynamics are modulated by asymmetric myocardial tolerance to global ischaemia. Among patients admitted after a VF-related cardiac arrest event, higher ARs prior to the first defibrillation shock are predictive of a favourable neurological outcome.

## Graphical Abstract

**Graphical Abstract:**
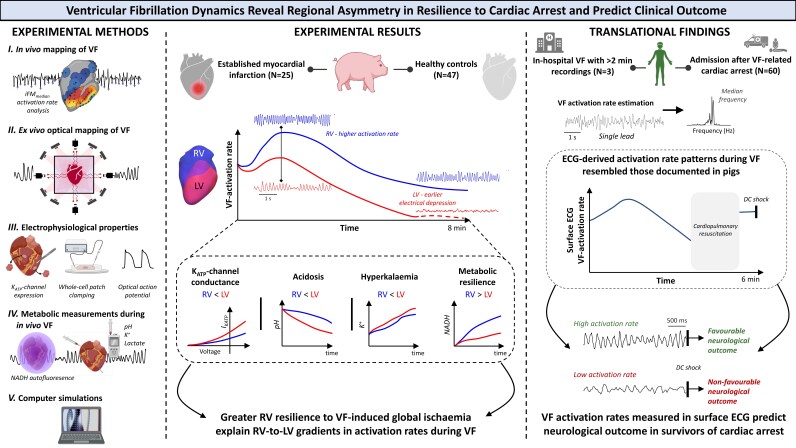

**Time of primary review: 29 days**

## 1. Introduction

Ventricular fibrillation (VF) is the most lethal cardiac arrhythmia and a major cause of sudden cardiac death, accounting for ∼300 000 deaths per year in the United States.^1^ The survival rate for out-of-hospital cardiac arrest is below 10% as the disorganized electrical activity of the ventricles disrupts the effective pumping of blood within seconds, causing global ischaemia and, eventually, death.^1^ Despite these critical implications, the mechanisms underlying early (seconds) and long-duration (minutes) VF episodes remain poorly understood.

Although VF may occur in individuals without a prior diagnosis of cardiac disease, myocardial ischaemia is a major cause of VF-driven cardiac arrest.^2^ Furthermore, the relative risk of VF is significantly higher in patients with a history of myocardial infarction (MI).^2^ Still, while recent reports have elucidated some electrophysiological mechanisms that may trigger and sustain initial VF wavefronts,^3^ their role beyond the first few seconds remains unclear. Understanding the underlying mechanisms of VF beyond these initial seconds is critical, given that interventions in patients with out-of-hospital cardiac arrest are usually delayed for several minutes.^4^ In this context, three global-ischaemia components intrinsically associated with VF are known to affect its temporal evolution. Two such components, acidosis and hyperkalaemia, contribute to reducing cell excitability and activation rates (AR).^5^ Meanwhile, hypoxia, the third component, activates the ATP-sensitive potassium current (*I*_KATP_) which shortens action potential duration (APD), thereby favouring re-entry and increasing the VF-AR.^5,6^ Still, the relative importance and specific time-course of each component during VF remain unknown. Moreover, dynamically evolving inter- and intra-ventricular myocardial heterogeneities may occur, giving rise to distinct AR domains and increasing the spatiotemporal complexity of the arrhythmia.^7^

We hypothesize that ischaemia-driven components during VF-related cardiac arrest are the dominant factors in the modulation of regional and temporal dynamics of VF ARs, regardless of the underlying substrate. Therefore, regional heterogeneities in ventricular ARs across VF episodes will be reproducible both within and between subjects, and driven by intrinsic differences in metabolic tolerance to ischaemia. Based on this, we propose that ventricular ARs during VF serve as dynamic indicators of ischaemia-induced damage, both in the heart and in critical organs relying on its effective pumping function. Therefore, ventricular ARs at the time of defibrillation will predict neurological outcomes in patients with VF-induced cardiac arrest. The study utilizes *in vivo* and *ex vivo* experimental settings, tissue sample analyses from pig models, and experimentally derived computational simulations to overcome the limitations of characterizing long-duration VF episodes in humans. The translational value of the experimental insights into the time-course of ECG signals during VF was further studied in patients with VF-related cardiac arrest.

## 2. Methods

### 2.1 Experimental design

The experimental studies included Large-white strain pigs with and without infarct-related substrate (*N* = 25 and *N* = 47, respectively). Animal experiments complied with ARRIVE guidelines and were performed at the Centro Nacional de Investigaciones Cardiovasculares (Madrid, Spain). All procedures were performed under general anaesthesia (intravenous ketamine 2 mg/kg per h, xylazine 0.2 mg/kg per h, and midazolam iv 0.2 mg/kg per h) and were approved by the competent ethical authority (Ref#PROEX097/17 & Ref#PROEX078.8/21), conforming to the regulations outlined in EU Directive 2010/63EU regarding the protection of animals used for scientific purposes. Pigs with infarct-related substrate underwent an ischaemia-reperfusion protocol affecting the left-descending coronary artery, as reported elsewhere.^8^ At a median of 14 (9, 28) weeks post-ischaemia-reperfusion, three-dimensional late-gadolinium enhancement cardiac magnetic resonance (LGE-CMR) was used to characterize the infarct-related substrate before further testing (see Supplementary material online, *Figure S1*).

Number of animals and characteristics within each experimental group are summarized in Supplementary material online, *Table S1*. In animals undergoing an *in vivo* study (see Supplementary material online, *Table S1A*), euthanasia was performed at the end of the procedure upon VF induction, subsequent asystole and non-recoverable cardiac arrest. In animals undergoing open-chest surgery (see Supplementary material online, *Table S1B*–*D*), the heart was exposed via median sternotomy under general anaesthesia (fentanyl 0.010 mg/kg iv, sevoflurane 3.5%). VF was induced with a 9 V battery, and afterwards euthanasia was performed by irreversible heart excision.

### 2.2 Invasive electrophysiological studies

**Figure 1:**
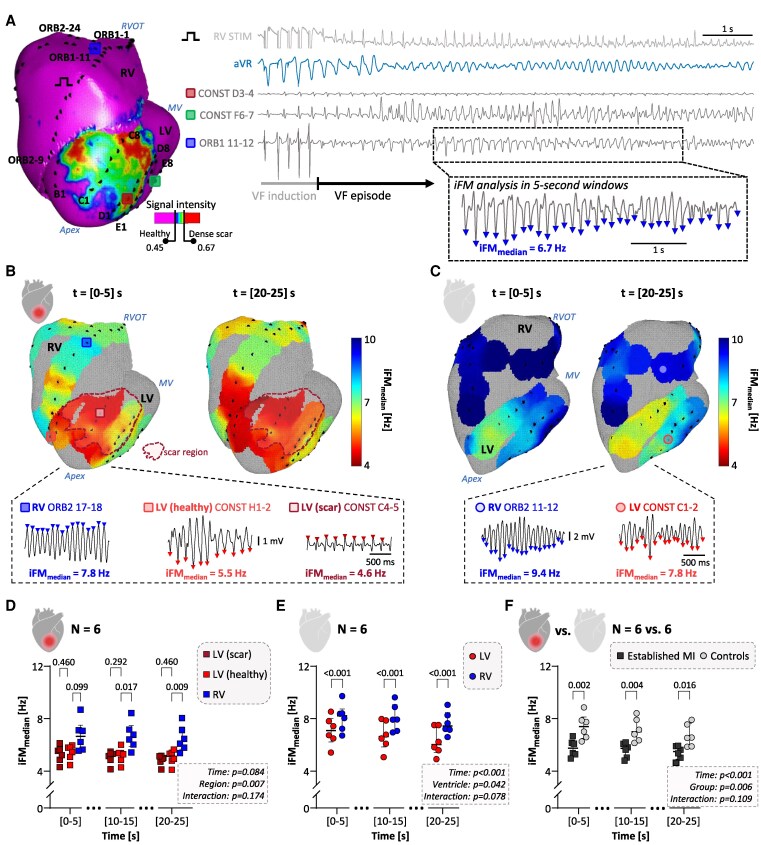
Early stages of *in vivo* ventricular fibrillation are independent of the infarct-related substrate and are characterized by higher activation rates in the right ventricle. (*A*) Left, sample visualization of catheter electrodes on the endocardial surface of the left and right ventricles (LV and RV, respectively) in an animal with established myocardial infarction (MI). MI characterization was performed using late gadolinium-enhancement cardiac magnetic resonance imaging. An 8 × 8-pole basket-catheter (CONST, labelled *A*–*H* and 1–8) was positioned inside the LV, and two 24-pole catheters (ORB1, ORB2) inside the RV. Right, sample surface ECG tracings and intra-cavitary electrograms during an induced ventricular fibrillation (VF) episode. Median values of instantaneous frequency modulations (iFM_median_) of bipolar signals were calculated in 5-s windows to yield an estimation of local activation rates. (*B*, *C*) Sample electrograms and 3D iFM_median_ maps at relevant time-windows during VF in an animal with MI (*B*) and a healthy control (*C*). (*D*) Temporal evolution of iFM_median_ during 30-s VF episodes in animals with MI. Data show the average iFM_median_ value of all electrodes inside the RV, non-infarcted (healthy) and infarcted LV regions (*N* = 6). (*E*) Temporal evolution of the average iFM_median_ during 30-s VF episodes from all electrodes inside the RV and the LV in healthy controls (*N* = 6). (*F*) Average iFM_median_ from all electrodes within the RV and LV, and the comparisons between animals with MI and controls at different time points of VF episodes (*N* = 6 for each group). In (*D*–*F*) data are shown as median and interquartile range. Two-way ANOVA followed by Tukey (*D*, *E*) or Sídák (*F*) *post hoc* correction was used. MV, mitral valve. RVOT, RV outflow tract.

Twelve pigs (*n* = 6 healthy controls, *n* = 6 established-MI) underwent an invasive *in vivo* electrophysiological study (EPS) to characterize early VF phases during short-duration VF (SDVF) episodes (*Figure 1A*, Supplementary material online, *Table S1A*). Percutaneous venous and arterial femoral access were used to reach the right and the left ventricles (RV and LV), respectively. A 64-pole basket catheter (Constellation, Boston Scientific, USA) was positioned in the LV. An additional basket catheter was placed within the RV. Alternatively, in cases with difficulty in properly deploying the basket catheter, two 24-pole catheters (Orbiter ST, Boston Scientific) were positioned towards the septum and the outflow tract of the RV. A screw-in catheter was positioned in the RV for stimulation. VF episodes were induced using basic drive pacing with a maximum of 4 coupled extrastimuli or burst pacing. Electrical signals from intracardiac catheters and surface ECG leads were continuously recorded throughout the experiment with the Labsystem PRO EP recording system (Boston Scientific). After induction and recording VF for 30 s, the episode was terminated with a 200-Joule biphasic DC shock. Between successive episodes, animals were allowed to recover to baseline conditions, which were monitored via ECG and invasive blood pressure measurements.

A second group of animals, including healthy controls (*n* = 5) and animals with established MI (*n* = 6), was studied during a single episode of long-duration VF (LDVF) of at least 8 min (see Supplementary material online, *Table S1A*). The animals underwent simultaneous endocardial and epicardial mapping using multipolar catheters in the LV and RV, as described for SDVF episodes, along with three additional 24-pole catheters positioned along the cardiac silhouette to cover both ventricles. Prior to VF induction, ventricular stimulation including progressively shorter coupling intervals was performed to obtain activation-recovery interval (ARI; a unipolar surrogate of APD) measurements from the LV and RV (see details in the Supplementary material).

In all animals, orthogonal fluoroscopic images were captured during the EPS and registered with the LGE-CMR geometry to project the position of each catheter electrode onto the corresponding LV/RV mesh (Supplementary material online, *Figure S2A*). Each endocardial electrode was assigned to one of nine endocardial regions (see details in the Supplementary material). Catheter electrodes located on the epicardium were assigned either to the LV or the RV (Supplementary material online, *Figure S2B*).

### 2.3 *In vivo* electrical signal processing

Surface ECG signals and intracardiac bipolar electrograms were processed offline with custom-made software in Matlab (MathWorks Inc., USA). Local VF-ARs were calculated using an instantaneous frequency modulation (iFM) analysis,^9^ adapted for bipolar recordings, over 5-s windows of the VF episode with a 50% window overlap to yield the iFM_median_. This provided an accurate estimate of ventricular AR every 2.5 s. Further details and examples on the iFM analysis of bipolar electrograms are described in the Supplementary material and Supplementary material online, *Figures S3–S5*. Furthermore, we developed a scoring method, termed *VF-leading score*, to rank endocardial regions based on their AR hierarchy across all 5-s windows of SDVF episodes. A regional score of 100 (or 0) indicates that all electrodes within a given region displayed the highest (or the lowest) ARs (iFM_median_ values) throughout all 5-s windows analysed within a SDVF episode (see Supplementary material online, *Figure S6*). Median frequency (F_median_) was used to estimate ventricular ARs from surface ECG signals during VF (see Supplementary material online, *Figure S7*). Details are provided in the Supplementary material.

### 2.4 *Ex vivo* optical mapping of transmembrane voltage changes

Optical mapping (OM) of transmembrane voltage changes was performed in 12 explanted pig hearts from animals with established MI (*n* = 6) and healthy controls (*n* = 6) to characterize epicardial VF dynamics during LDVF episodes (Panoramic OM protocol, Supplementary material online, *Table S1B*). After euthanasia and heart excision, hearts were connected to a Langendorff perfusion system using Tyrode’s solution and submerged in a custom-built, temperature-controlled, 25-L tank filled with saline (maintained at 37°C).^10^ Hearts were loaded upstream the aortic cannula with voltage-sensitive dye (di-4-ANEQ(F)PTEA). Oxygen was removed from the saline superfusion via nitrogen purging until dissolved O_2_ was <5%. VF was induced by ventricular burst pacing and, after which Langendorff-perfusion was halted. The setting aimed to replicate global ischaemia conditions of cardiac arrest *in vivo* upon VF induction. Panoramic optical movies were recorded continuously for at least 8 min using four evenly-spaced CMOS high-speed cameras (120 × 160 superpixels, 400 frames/sec). iFM analysis was performed on optical signals over 5-s windows,^9^ and the iFM_median_ was used as an estimator of the local AR during VF. Pixels from the four camera views were assigned to one of thirteen epicardial regions. Then, iFM_median_ values of pixels within each region were averaged to yield a 2D-schematic epicardial representation.

Seven additional hearts from healthy pigs were used for ratiometric optical measurements of transmembrane voltage changes using optical fibres. This protocol aimed to identify regional epicardial differences in APD (Ratiometric OM protocol, Supplementary material online, *Table S1B*). Briefly, the distal end of a single-core plastic optical fibre (2–3 mm diameter) was sequentially and gently pressed against the surface of eight ventricular regions during ventricular pacing. Blue (465–495 nm) and red (632–652 nm) lights were used to excite the dye-loaded heart surface and generate fluorescence emission corresponding to numerator and denominator signals, respectively. The ratio of these signals yielded optical action potentials with minimal contraction artefact.^11^ The resulting signals at each region and pacing rate were processed offline. Details on OM are described in the Supplementary material.

### 2.5 Gene and protein expression of the ATP-sensitive potassium channel

Twelve animals (healthy controls, *n* = 5; established-MI, *n* = 7) underwent open-chest surgery to expose the heart. Fresh epicardial samples from the anterior wall of the LV and the RV were collected using a scalpel (K_ATP_ gene/protein expression protocol, Supplementary material online, *Table S1C*). Samples were immediately snap-frozen and later used for RT-qPCR and immunoblotting to measure gene and protein expressions of K_ATP_ channel subunits, respectively (see also the Supplementary material).

### 2.6 Whole-cell voltage-clamping in isolated cardiomyocytes

Isolated cardiomyocytes were obtained from the LV and RV of six healthy controls (Whole-cell patch clamp protocol, Supplementary material online, *Table S1C*). The explanted pig heart was cannulated and perfused through a Langendorff-perfusion system. Myocardial samples from the anterior wall of the LV and RV were chopped into chunks and enzymatically digested.^12^ Isolated rod-shaped cardiomyocytes were placed in a perfusion chamber with normal Tyrode’s solution. Whole-cell voltage-clamp recordings were performed as reported elsewhere.^12^  *I*_KATP_ was calculated by subtracting currents recorded in the absence or presence of pinacidil 100 μM (see also the Supplementary material).

### 2.7 Coronary vein blood measurements during VF *in vivo*

Myocardial resilience during global ischaemia was assessed *in vivo* during LDVF episodes in seven healthy pigs. Electrolytes and metabolic indicators (e.g. pH, lactate, blood gases) were measured in blood samples from LV and RV coronary veins (Coronary-vein blood protocol, Supplementary material online, *Table S1D*). Under general anaesthesia, animals underwent open-chest surgery to expose the heart. After baseline measurements in sinus rhythm, blood samples (∼100 μL) were drawn from the RV and LV coronary veins using 24-G lines (0.7 × 19 mm; BD Vialon, BD, USA). At least two samples from each vein were taken sequentially during LDVF. The analysis was performed using the epoc® Blood Analysis System (Siemens Healthineers, Germany). Blood from the coronary sinus was collected as reference. Details are shown in the Supplementary material.

### 2.8 *In vivo* mapping of cardiac NADH autofluorescence during VF

Nicotinamide Adenine Dinucleotide + Hydrogen (NADH) autofluorescence was measured during VF in an additional five healthy pigs (In vivo NADH protocol, Supplementary material online, *Table S1D*) as an indirect marker of aerobic-respiration disruption.^13^ Following anaesthesia, the heart was exposed via median sternotomy. An ultra-violet light lamp (365 nm) was used to homogeneously illuminate the antero-lateral walls of both ventricles. After inducing VF with a 9-V battery, NADH autofluorescence (460 nm) was imaged for 10 min using a CMOS camera (200 × 240 superpixels). Movies were processed off-line to analyse RV and LV autofluorescence during VF within a region of interest with similar baseline fluorescence. Details are provided in the Supplementary material.

### 2.9 Computational simulations

Experimental measurements from the LV and the RV during VF were incorporated into well-established *in silico* ventricular models to replicate the different electrical properties of LV and RV cardiomyocytes during global ischaemia. Action potentials and underlying ionic currents were computed using the O’Hara model, incorporating modifications by Ferrero *et al*. to simulate ischaemia and *I*_KATP_.^14,15^ Single-cell action potentials were generated by pacing the cell at different cycle lengths under normoxic and ischaemic conditions. Tissue simulations were carried out by defining a monodomain virtual anisotropic tissue with two adjacent 5 × 5 cm^2^ halves, representing LV and RV models under ischaemia. Fibrillation was induced using a S1-S2 cross-shock stimulation. Virtual bipoles were positioned 1 mm above the simulated tissue to capture *in vivo*-like bipolar recordings during fibrillation (see the Supplementary material for details).

### 2.10 Clinical study in humans

VF episodes with >2 min of continuous ECG telemetry before defibrillation were used to analyse the time-course of ventricular ARs in three patients with in-hospital cardiac arrest (see Supplementary material online, *Figure S8A*). The only available lead (II) was digitized and divided into 5-s segments with 50% temporal overlap. F_median_ was used to estimate ARs during VF, as described for ECG tracings in pigs (see Supplementary material online, *Figure S8B*). An additional group of 60 patients admitted to the hospital in a comatose status after VF-related cardiac arrest was used to study the clinical value of VF ARs in predicting neurological performance at hospital discharge (see Supplementary material online, *Figure S8A*). Patients were classified according to their neurological performance using the Pittsburgh outcome categorization of brain injury. Patients were recruited from two institutions in Spain (Hospital Clínico San Carlos and Hospital Universitario La Paz, Madrid). The ethics review committee approved the studies, in accordance with the ethical guidelines of the Declaration of Helsinki and European guidelines for good clinical practice. All patients or their legally authorized representative provided written informed consent. Details are provided in the Supplementary material.

### 2.11 Statistical analysis

Data normality was assessed using the Shapiro-Wilk test. Data are expressed as median and interquartile range, or mean and 95% confidence interval. Comparisons between two groups were performed using the unpaired/paired *t*-test, or the Mann–Whitney/Wilcoxon test, as appropriate. For variables with a normal distribution, comparisons between more than two groups were performed using one- or two-way analysis of variance (ANOVA) followed by Tukey´s or Sídák´s *post hoc* tests. For non-normally distributed variables, Dunn´s or Holm-Sídák correction was applied for multiple comparisons. For patch-clamp data analysis, individual values of cardiomyocytes were averaged per animal and ventricle, and these mean values were used for statistical testing. In patients, univariate logistic regression was performed to identify clinical variables statistically associated with neurological outcome. For variables with *P-*value < 0.05, odds ratios and receiver operating characteristic area under the curve (AUROC) values were used to assess predictive performance. AUROC values are reported with their 95% confidence intervals. Comparisons between AUROC were performed using DeLong’s test. A two-tailed *P*-value < 0.05 was considered statistically significant. Analyses and data visualization were performed with GraphPad Prism10 (GraphPad Software Inc., CA) or Matlab.

## 3. Results

### 3.1 *In vivo* early VF phases show faster activation rates in the RV than in the LV, independent of an infarct-related substrate

In animals with established MI, the fibrotic substrate was located primarily in the LV (*Figure 1A*). *Figure 1B* and *C* show sample iFM_median_ maps at significant time windows during SDVF episodes in an animal with established MI and in a healthy control, respectively. Overall, during the first SDVF episode, animals with established MI showed significantly higher iFM_median_ values in bipoles located in the RV compared to bipoles located in healthy or scarred regions of the LV (e.g. *t* = [20–25] s; RV: 6.3 [5.7, 7.4] Hz; LV healthy: 5.1 [4.6, 5.5] Hz; LV scar: 5.0 [4.7, 5.8] Hz; RV vs. LV healthy: *P* = 0.009; LV healthy vs. LV scar: *P* = 0.460, *Figure 1D*). Similarly, in healthy controls, iFM_median_ values were significantly higher in the RV than in the LV during all the time-windows of first SDVF episodes (e.g. *t* = [20–25] s; RV: 7.4 [7.0, 8.5] Hz, LV: 6.0 [5.4, 7.5] Hz; *P* < 0.001, *Figure 1E*). The overall average iFM_median_ from both ventricles was significantly lower in animals with established MI than in healthy controls across all time windows (e.g. *t* = [20–25] s: 5.5 [4.9, 5.9] Hz vs. 6.5 [5.9, 7.6] Hz, *P* = 0.016, *Figure 1F*).

Furthermore, regional hierarchies in AR were analysed using the *VF-leading score* (see Supplementary material online, *Figure S9A*). In the first SDVF episode induced in animals with established MI, RV regions showed a statistically higher average VF-leading score than LV regions (76.1 [70.2, 78.6] vs. 39.1 [36.2, 41.3], *P* < 0.001, Supplementary material online, *Figure S9B*). In the RV endocardium, no significant intra-ventricular differences emerged among regions, suggesting homogeneous behaviour within the RV (*P* = 0.100, Supplementary material online, *Figure S9B*). Similarly, in healthy controls, RV regions showed a statistically higher average VF-leading score than LV regions (72.2 [62.2, 81.7] vs. 41.2 [34.3, 45.8], *P* = 0.008, Supplementary material online, *Figure S9C*), without statistically significant differences among RV regions (*P* = 0.154, Supplementary material online, *Figure S9C*). This pattern was consistent throughout the four additional SDVF episodes induced per animal, in both established-MI animals and healthy controls (see Supplementary material online, *Figure S10*).

### 3.2 Activation-rate gradients consolidate on the endocardium and emerge on the epicardium during *in vivo* long-duration VF episodes

**Figure 2:**
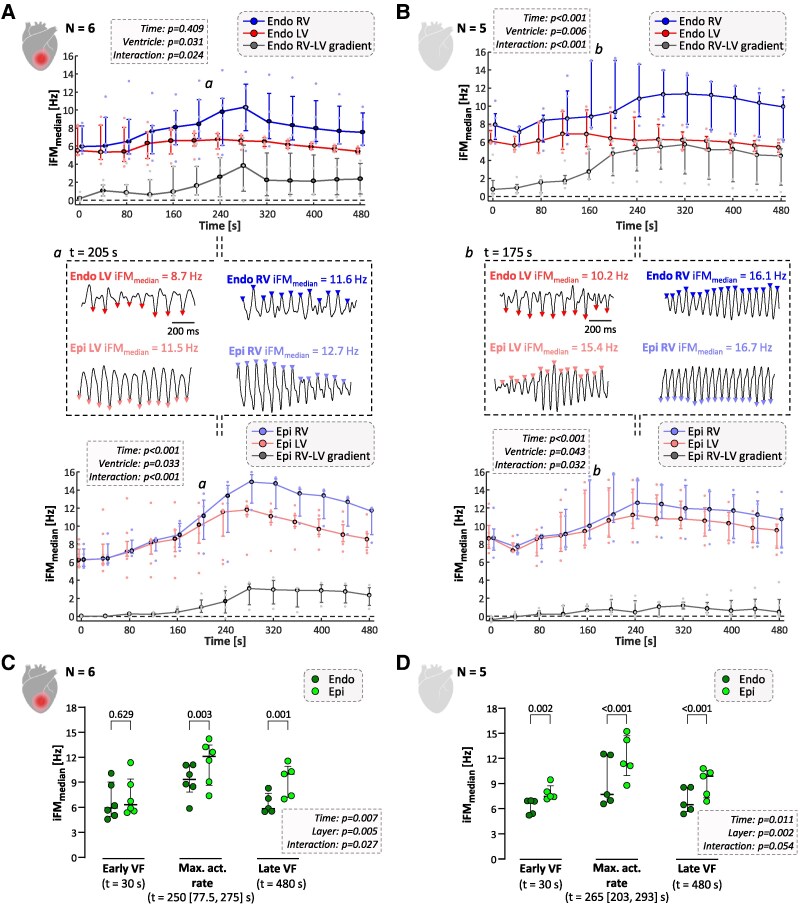
Right-to-left and epicardium-to-endocardium activation rate gradients consolidate during long-duration episodes of *in vivo* ventricular fibrillation. (*A*, *B*) Temporal evolution of the average median instantaneous frequency modulation (iFM_median_) of the bipoles located on the endocardium (top row) and epicardium (bottom row) of the left and right ventricles (LV and RV, respectively) during long-duration ventricular fibrillation (VF). Electrograms were recorded *in vivo* in animals with established myocardial infarction (MI) (*A*) and healthy controls (*B*) (*N* = 6 and *N* = 5, respectively). The RV-to-LV gradient, calculated as their paired difference, is shown in grey. Middle row, 1-s tracings and iFM annotations from sample bipoles located on the epicardium and the endocardium of the LV and RV (additional examples in Supplementary material online, *Figure S11*, *S12*). Data are expressed as median and interquartile range. In two animals with MI, epicardial access to the RV was not possible (*N* = 4 for RV epicardial and RV-to-LV epicardial gradient data). (*C*, *D*), Quantification and comparison of the average iFM_median_ of the endocardium and the epicardium at specific time points (end of early-VF – 30 s; time-of-maximal activation rate; late-VF – 8 min) in animals with MI (*C*) and controls (*D*) (*N* = 6 and *N* = 5, respectively). In one infarcted pig, the VF episode could not be recorded for 8 min. In all panels, two-way ANOVA followed by Sídák *post hoc* correction was used.

*Figure 2A* and *B* show the time-course of the iFM_median_ values during LDVF in animals with established MI and healthy controls, respectively. All animals experienced an acceleration phase in which ARs increased (time-of-maximal AR: 250 [77.5, 275] s in established MI; 265 [203, 293] s in controls, *Figure 2C* and *D* and Supplementary material online, *Figure S11*, *S12*), followed by a deceleration phase. The endocardial RV-to-LV AR gradient documented during SDVF was exacerbated during later stages of VF, and was statistically significant in both pigs with MI (max. gradient: 3.8 [0.98, 6.1] Hz at *t* = 280 s, overall RV-vs.-LV: *P* = 0.031, *Figure 2A*) and controls (max. gradient: 5.8 [2.5, 6.3] Hz at *t* = 320 s, overall RV-vs.-LV: *P* = 0.006, *Figure 2B*).

In the epicardium, the RV-to-LV AR gradient was also present in both animals with MI (max. gradient: 3.0 [1.4, 4.0] Hz, *t* = 320 s, overall RV-vs.-LV: *P* = 0.033, *Figure 2A*) and in controls (max. gradient: 1.2 [0.8, 1.6] Hz, *t* = 320 s, overall RV-vs.-LV: *P* = 0.043, *Figure 2B*). Moreover, epicardial bipoles showed significantly higher average iFM_median_ values than endocardial bipoles at the time-of-maximal AR in both groups (established MI: 12.1 [8.7, 13.5] Hz vs. 9.4 [7.8, 11.1] Hz, *P* = 0.003, *Figure 2C*; healthy controls: 11.4 [10.0, 14.7] Hz vs. 7.7 [6.8, 12.5] Hz, *P* < 0.001, *Figure 2D*). These gradients were maintained until later stages of VF (*t* = 8 min, established MI: 10.0 [7.2, 10.9] Hz vs. 5.9 [5.5, 7.7] Hz, *P* = 0.001, *Figure 2C*; healthy controls: 9.9 [7.3, 10.5] Hz vs. 6.5 [5.7, 8.6] Hz, *P* < 0.001, *Figure 2D*).

### 3.3 *Ex vivo* panoramic OM confirms RV-to-LV epicardial activation rate gradients during VF and reveals earlier electrical depression in the LV

**Figure 3:**
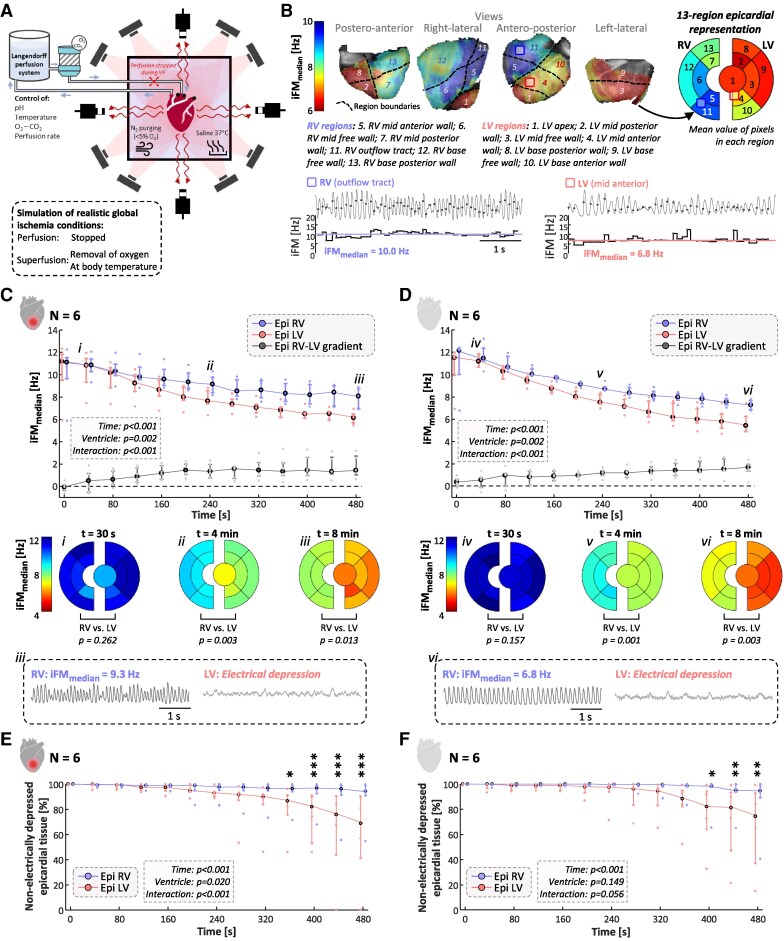
*Ex vivo* panoramic optical mapping demonstrates right-to-left epicardial activation rate gradients and earlier electrical depression in the left ventricle during long-duration ventricular fibrillation. (*A*) Schematic of a panoramic optical mapping setting (created with *BioRender.com*) aiming to replicate global ischaemia during ventricular fibrillation (VF). (*B*) Four mapping views and sample 2D-schematic visualization of median instantaneous frequency (iFM_median_) values during a VF episode in a healthy heart. Pixels within the regions delimited by dashed black lines are averaged onto the 13-region 2D-schematic visualization. Below, iFM analysis of sample optical signals from the right ventricle (RV) and the left ventricle (LV). (*C*, *D*) Top row, temporal evolution of the average iFM_median_ of all the pixels on the LV and the RV surface during long-duration VF episodes in hearts with established myocardial infarction (MI) (*C*) and controls (*D*) (*N* = 6 for each group). The RV-to-LV gradient, calculated as their paired difference, is shown in grey. Middle row, 2D representation of the average iFM_median_ of each epicardial region at significant time points, with the inter-ventricular comparison for the average of all regions in each ventricle (complete data distribution shown in Supplementary material online, *Figure S15*). Bottom row, sample optical signals from the RV and the LV during late stages of VF, showing electrical depression in the LV. (*E*, *F*) Proportion of non-electrically depressed epicardial surface of the RV and LV over the time-course of long-duration VF episodes in hearts with MI (*E*) and controls (*F*) (*N* = 6 for each group). All quantifications are shown as median and interquartile range. Two-way ANOVA followed by Sídák *post hoc* correction was used. In (*E*, *F*) specific comparisons with *P < 0.05; **P < 0.01; ***P < 0.001 are shown to clarify the earlier onset of electrical depression in the LV.

Given the *in vivo* findings, a panoramic OM setup, replicating the global ischaemia conditions of VF *in vivo*, was used to characterize LDVF episodes on the epicardial surface at high spatial resolution (*Figure 3A* and *B*). The epicardial RV-to-LV gradient in iFM_median_ values was statistically significant in hearts with established MI (1.4 [1.0, 2.7] Hz gradient after 8 min in VF; overall RV-vs.-LV: *P* = 0.002, *Figure 3C*, Supplementary material online, *Figure S13*) and in healthy controls (1.7 [1.4, 2.0] Hz gradient after 8 min; overall RV-vs.-LV: *P* = 0.002, *Figure 3D*, Supplementary material online, *Figure S14*). The RV showed progressively faster ARs than the LV during LDVF episodes (*Figure 3C* and *D*, Supplementary material online, *Figure S15*). The LV showed earlier signs of electrical depression and, after 8 min of VF, the proportion of the epicardial surface with preserved optical action potentials was significantly lower in the LV than in the RV, both in hearts with MI (LV: 68.9% [41.0%, 89.7%] vs. RV: 94.2% [90.8%, 98.6%], *P* < 0.001, *Figure 3E*) and controls (LV: 74.4% [36.9%, 92.8%] vs. RV: 94.7% [89.1%, 98.7%], *P* = 0.005, *Figure 3F*). Altogether, data from LDVF episodes suggest that the RV may exhibit greater resilience to VF-induced global ischaemia than the LV, enabling the RV to maintain higher ARs after several minutes in VF.

### 3.4 Interventricular differences in baseline electrophysiological parameters and the pinacidil-induced ATP-sensitive K^+^ current do not explain activation-rate gradients during VF

**Figure 4:**
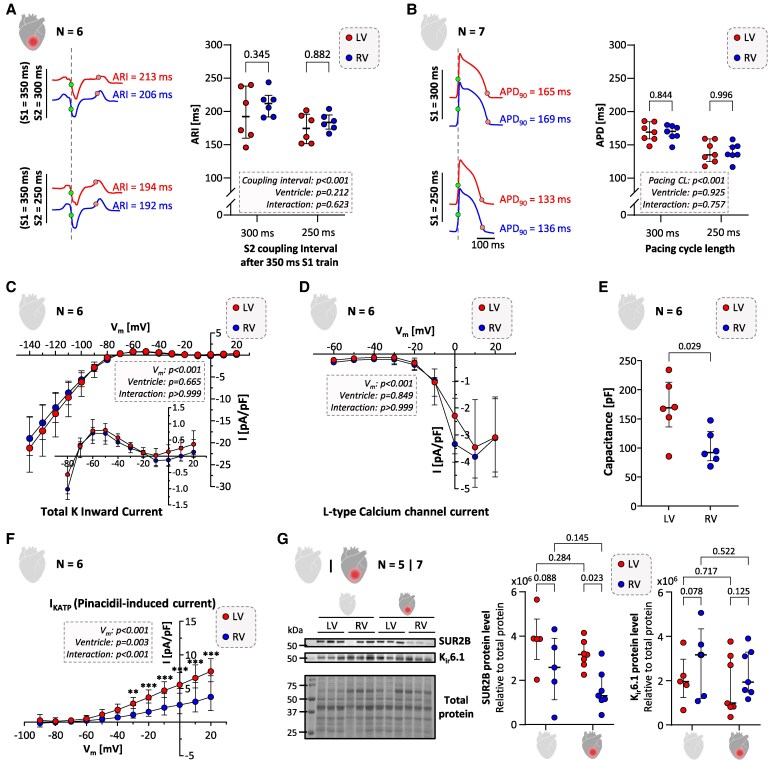
Interventricular differences in baseline action potential duration and in the maximal conductance of K_ATP_ channels do not explain the right-to-left activation rate gradients observed during ventricular fibrillation. (*A*) *In vivo* activation-recovery intervals (ARI) from the endocardium of the left and right ventricles (LV and RV, respectively) in pigs with established myocardial infarction (MI) (*N* = 6. Additional data in Supplementary material online, *Figure S16*). (*B*) *Ex vivo* average LV and RV optical action potential durations (APD) in healthy pig hearts (*N* = 7. Additional data in Supplementary material online, *Figure S17*). (*C*, *D*) Current-voltage (I-V) relationship of total K^+^ inward (*C*) and L-type calcium-channel currents (*D*) in LV and RV isolated cardiomyocytes from healthy animals (*N* = 6). (*E*) Cell-membrane capacitance of LV and RV cardiomyocytes. (*F*) I-V relationship of the inward ATP-sensitive potassium current (*I*_KATP_) in LV and RV cardiomyocytes. (*G*) Immunoblots and protein quantification of K_ir_6.1 and SUR2B K_ATP_ channel subunits in pigs with MI and controls, relative to the total protein (*N* = 5 and *N* = 7, respectively). In all panels, data expressed as median and interquartile ranges. In (*C*–*F*) data represent the average of cardiomyocytes within each pig and ventricle (*N* = 6 pigs; *n* = 16 and *n* = 14 cardiomyocytes for LV and RV, respectively). In (*A*–*D*, *F*) two-way ANOVA followed by Sídák *post hoc* correction was used. In (*E*) paired *t*-test was performed. *P < 0.05, **P < 0.01, ***P < 0.001.

In animals with MI*, in vivo* ARI measurements from the endocardium of the RV and LV were not significantly different during S1 or S1-S2 pacing (*Figure 4A* and Supplementary material online, *Figure S16*). Similarly, epicardial action potentials measured *ex vivo* in healthy controls using optical fibres and ratiometry (see Supplementary material online, *Figure S17A*, *S17B*) showed that the shorter the pacing cycle length, the more homogeneous was the APD between the RV and LV (*Figure 4B*), and among intraventricular regions (see Supplementary material online, *Figure S17C*). Furthermore, no significant differences were observed in the optical upstroke duration of the action potential between the RV and LV (see Supplementary material online, *Figure S17D*).

Whole-cell voltage-clamp recordings in isolated cardiomyocytes from the LV and RV (*n* = 16 and *n* = 14, respectively) showed no differences in total inward K^+^ current (*P* = 0.665, *Figure 4C*) or L-type calcium current (*P* = 0.849, *Figure 4D*). Isolated LV cardiomyocytes exhibited a significantly higher capacitance compared to those from the RV (169 [136, 213] pF vs. 92 [78, 128] pF, *P* = 0.029, *Figure 4E*). In the presence of 100 µM pinacidil, *I*_KATP_ was significantly higher in isolated LV cardiomyocytes than in RV cardiomyocytes at voltage values higher than −30 mV (*P* = 0.003, *Figure 4F*). Further analyses of the five K_ATP_ channel subunits showed, across animal models and in both ventricles, gene expression levels of K_ir_6.1 > SUR2B > K_ir_6.2 > SUR2A. No gene expression was detected for SUR1 (see Supplementary material online, *Figure S18A*, *S18B*). K_ir_6.1 showed no interventricular differences at the transcript or protein level in any group (*Figure 4G*, Supplementary material online, *Figure S18A*, *S18B*). In healthy animals, SUR2B mRNA expression was significantly higher in the LV than in the RV (*P* = 0.001, Supplementary material online, *Figure S18A*). In animals with MI this difference was statistically significant at the protein level (*P* = 0.023, *Figure 4G*). In healthy animals, K_ir_6.2-expression was also significantly higher in the LV than in the RV at both the transcript and protein levels (*P* = 0.009 and *P* = 0.036, respectively, Supplementary material online, *Figure S18A*, *S18C*). No interventricular differences were observed in SUR2A gene or protein expression in any group (see Supplementary material online, *Figure S18A*, *S18C*). These findings suggest that *I*_KATP_ might contribute to an AR gradient in the opposite direction to that observed experimentally. Therefore, other ischaemia-associated factors must contribute to the genesis of the observed RV-to-LV AR gradients.

### 3.5 The RV exhibits greater resilience than the LV to global myocardial ischaemia during VF

**Figure 5:**
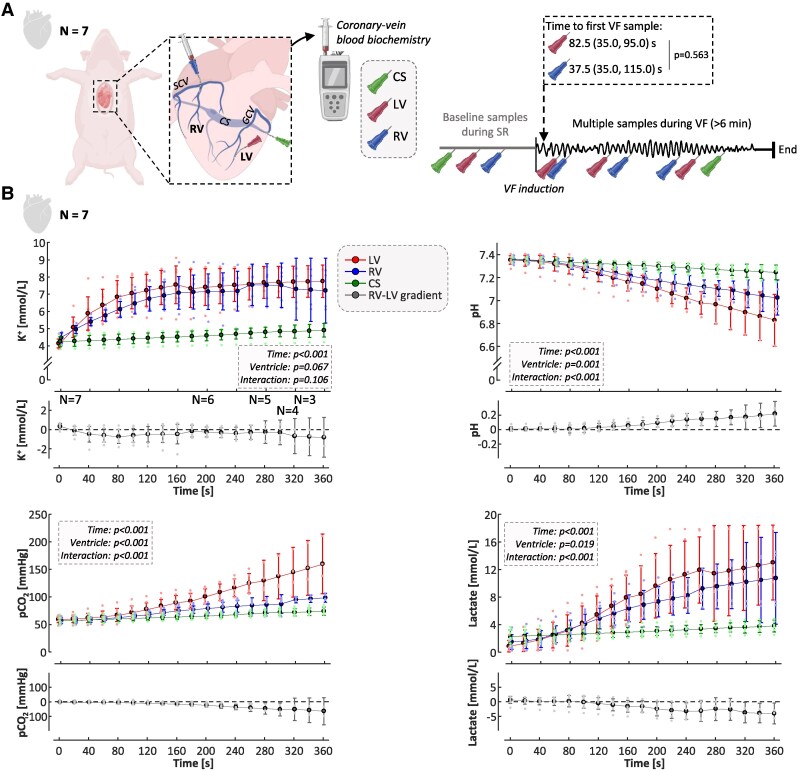
Time-course of metabolism-related parameters within the left and right ventricles during *in vivo* ventricular fibrillation. (*A*) Schematic of the experimental setting (created with *BioRender.com*). The time-to-first VF blood sample is expressed as median and interquartile range, and paired *t*-test was performed for comparison. (*B*) Evolution of K^+^, pH, pCO_2_ and lactate values measured over the course of ventricular fibrillation (VF) in blood samples from the coronary sinus (CS), and epicardial coronary veins of left and right ventricles (LV and RV, respectively). Parameters were assumed to follow a linear progression between samples and until the last sample. Data are shown as mean and 95% confidence interval. The paired RV-to-LV gradient is shown in grey. The number of animals (*N* = 7) for comparisons is the same in all graphs (updated whenever the last sample was taken in a pig). Two-way ANOVA followed by Sídák *post hoc* correction was used. GCV/SCV: great/small cardiac vein. SR: sinus rhythm.

After baseline measurements during sinus rhythm (see Supplementary material online, *Figure S19*), blood samples from LV and RV epicardial veins (3 [2, 4] samples and 3 [2, 3] samples, respectively) were drawn at different time points during LDVF (*Figure 5A*). Hyperkalaemia rapidly developed in both ventricles over the course of ischaemia, but K^+^ levels tended to be higher in LV blood samples than in those from the RV (6 min after VF onset, LV: 7.76 ± 0.93 mmol/L, RV: 7.21 ± 1.88 mmol/L; *P* = 0.067, *Figure 5B*). Ischaemia-induced acidosis progressively decreased the pH in both ventricles, although to significantly lower values in samples from LV veins than in those from the RV (6 min after VF onset, LV: 6.83 ± 0.23, RV: 7.02 ± 0.15, respectively; *P* = 0.001, *Figure 5B*). Lactate and pCO2 confirmed a higher acidosis in LV samples compared to those from the RV (*Figure 5B*). Additional parameters are detailed in Supplementary material online, *Figure S20*.

Cardiac NADH autofluorescence was further measured *in vivo* in 5 healthy pigs at baseline and during VF (see Supplementary material online, *Figure S21*, *S22*). After 160 s in VF, the epicardial surface showed a significant RV-to-LV gradient in relative NADH autofluorescence (gradient: 7.5 [1.3, 17.7], *P* = 0.021, Supplementary material online, *Figure S22A*, *S22B*) which continued to increase until the end of the recording at 10 min (gradient at *t* = 9 min: 19.5 [7.4, 22.7], *P* < 0.001, Supplementary material online, *Figure S22B*).

### 3.6 Computational simulations support the finding that right-to-left activation rate gradients during VF are related to the greater resilience of the RV to ischaemia

**Figure 6:**
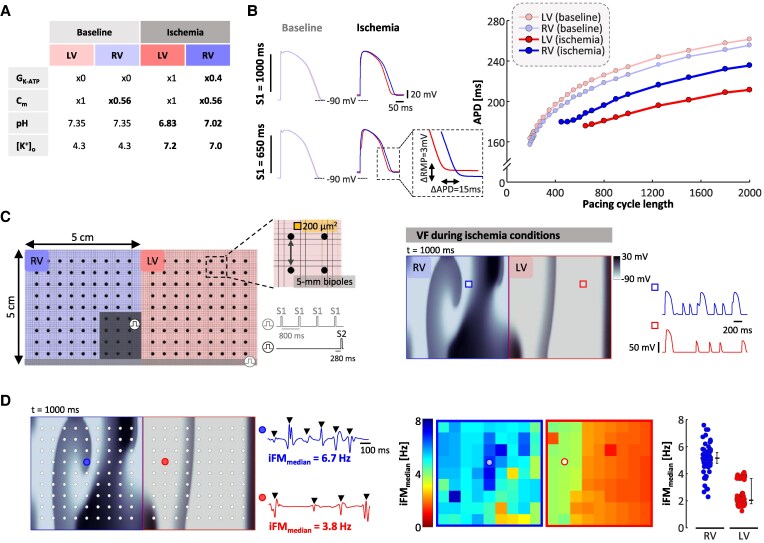
Computational simulations insights into the role of ischaemia components in generating right-to-left activation rate gradients during ventricular fibrillation. (*A*) Summary of experimentally derived parameters included in the simulations for the left and right ventricles (LV and RV, respectively). (*B*) Left, simulated action potentials and, right, restitution curves of action potential duration (APD) from single cell simulations under normoxic and ischaemic conditions. (*C*) Left, schematic view of the 2D virtual tissue incorporating the LV and RV parameters derived from the experimental data. Virtual electrodes were located 1 mm above the tissue and evenly distributed (black dots). Right, snapshot of a fibrillatory episode induced under ischaemic conditions. Sample action potentials from RV and LV locations are also shown. (*D*) Left, snapshot of a simulated fibrillatory episode with sample bipolar electrograms from virtual electrodes on the RV and LV and their median instantaneous frequency modulation (iFM_median_) values. Right, RV and LV maps and distribution of iFM_median_ values from all the simulated bipolar signals (*n* = 81 for each map). RMP: resting membrane potential.

Experimental results suggest that ARs during VF are modulated by greater acidosis and higher extracellular K^+^ concentrations in the LV. These factors may counterbalance the effect of a higher LV K_ATP_ channel conductance, yielding lower excitability in the LV than in the RV. This mechanism likely underlies the RV-to-LV AR gradients observed during VF. To test this hypothesis, computational models incorporated experimentally derived data on LV and RV cell capacitances, K_ATP_ channel conductance, hyperkalaemia and acidosis (*Figure 6A*). Consistent with the experimental data, single-cell simulations during pacing showed similar APD values in the RV and the LV under normoxic conditions, particularly at short cycle lengths (*Figure 6B*). However, under ischaemia, APDs shortened to a greater extent in the LV, likely due to a higher K_ATP_-channel conductance. Under ischaemia, the LV also showed a more depolarized resting membrane potential compared to the RV. This difference was associated with a loss of excitability in the LV at short pacing cycle lengths, at which the RV remained excitable (*Figure 6B*). Further simulations in a two-dimensional anisotropic tissue model, incorporating the RV-LV differential adaptation to VF-induced global ischaemia (*Figure 6C*), revealed an ∼3 Hz right-to-left AR gradient in bipolar signals recorded from virtual bipoles positioned 1 mm above the tissue (*Figure 6D*).

### 3.7 ECG-derived activation rate patterns during VF are similar in humans and experimental pig models

**Figure 7:**
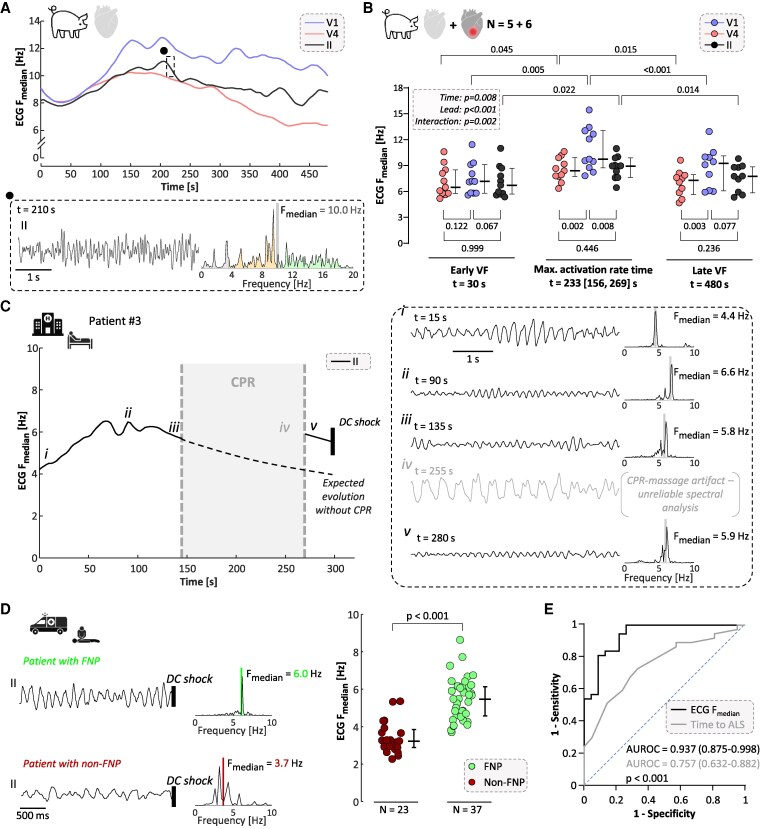
Translational and prognostic value of ECG-derived VF activation rates after cardiac arrest. (*A*) Time-course of median frequency (F_median_) values in V1, V4, and II leads during a long-duration ventricular fibrillation (VF) episode in a healthy pig. Below, sample 5-s ECG tracing and frequency-domain analysis at a specific timepoint (coded with a black dot). (*B*) Quantification of the F_median_ in V1, V4, and II leads at early stages of VF (*Early* VF—30 s), at the time-of-maximal activation rate, and at late stages of VF (*Late* VF—8 min) in animals with and without established myocardial infarction (*N* = 11 [*N* = 10 in *Late* VF since in one pig the VF episode was recorded for less than 8 min]). (*C*) Left, sample time-course of the F_median_ in lead II in a patient with an in-hospital VF episode. Spectral analysis during cardiopulmonary resuscitation (CPR) manoeuvres was not possible due to motions artefact (shaded in grey) and until CPR was interrupted before the direct-current (DC) shock. The expected evolution of F_median_ without CPR is sketched (dashed black line) to highlight the influence of CPR manoeuvres on the activation rate. Right, sample ECG tracings and frequency-domain analysis at specific timepoints (coded *i-v*). (*D*) Left, sample F_median_ values from ECG tracings of patients with out-of-hospital VF and immediately prior to the first DC-shock. Right, comparison of F_median_ values between patients with favourable neurological performance (FNP) and non-FNP at hospital discharge (*N* = 37 and 23, respectively). Data are expressed as median and interquartile range. Mann–Whitney test was performed. (*E*) Comparison of receiver operating characteristic curves (ROC) derived from F_median_ (black) values and the time-to-advanced life support (ALS) (grey) to predict FNP at hospital discharge in patients admitted to hospital in comatose status after a VF event. Comparison between ROC curves was performed using DeLong’s test. Area under the curve values (AUC) are expressed with the 95% confidence interval. Illustrations in (*C*) and (*D*) were created with *BioRender*.

Ventricular AR patterns on surface ECG tracings may have translational value in clinical settings of VF-related cardiac arrest. ARs on surface ECG leads V1, V4, and II (primarily reflecting the electrical activity of the RV, the LV, and the entire myocardium, respectively, Supplementary material online, *Figure S7*) were analysed during *in vivo* LDVF episodes in a group of 6 animals with established MI and 5 healthy controls. A samples case is shown in *Figure 7A*. Overall, the temporal dynamics of RV and LV local electrograms were observable in surface ECG tracings. Lead V1 displayed the highest F_median_ values at the time of maximal AR (V1: 9.7 [8.8, 13.0] Hz; V4: 8.4 [7.7, 9.9] Hz, *P* = 0.002 vs. V1; II: 8.9 [7.6, 9.9] Hz, *P* = 0.008 vs. V1, *Figure 7B*). The decrease in F_median_ values after the acceleration stage was also observable across the three leads (*Figure 7B*).

The same analysis was applied to ECG tracings from three patients who experienced in-hospital VF (see Supplementary material online, *Figure S8A*). In the available lead II, F_median_ values showed an initial acceleration phase in ARs followed by a deceleration phase, remarkably similar to the temporal AR patterns in pigs (*Figure 7C*, Supplementary material online, *Figure S23*). In all patients, cardiopulmonary resuscitation (CPR) manoeuvres were performed after the AR acceleration phase, at a point when, based on the experimental data, ARs would have been expected to continue declining in the absence of intervention. Interestingly, CPR manoeuvres increased F_median_ values before the DC shock exceeding the values expected without CPR (*Figure 7C*, Supplementary material online, *Figure S23*). This suggests that CPR manoeuvres mitigated the ischaemic conditions associated with VF.

### 3.8 VF activation rates predict neurological outcome in comatose survivors of cardiac arrest

Given that VF-ARs represent a direct indicator of cardiac status, they may also serve as an indirect marker of the status of sensitive organs such as the brain, which relies on the heart´s effective pumping function. This was evaluated in a retrospective cohort of 60 patients admitted to the hospital in comatose status after VF-related cardiac arrest (see Supplementary material online, *Tables S2*, Supplementary material online, *Figure S8A*). Patients with favourable neurological performance at hospital discharge (FNP, *N* = 37) showed significantly higher F_median_ values on lead II tracings at the time of the first DC shock than patients without FNP (5.5 Hz [4.7, 6.1] Hz vs. 3.2 Hz [2.9, 3.9] Hz, respectively, *P* < 0.001, *Figure 7D*). Moreover, F_median_ values were a strong predictor of neurological outcome at hospital discharge, significantly outperforming the best clinical variable (i.e. time-to-advanced life support; AUROC = 0.937 vs. AUROC = 0.757, respectively, *P* < 0.001, *Figure 7E*, Supplementary material online, *Table S3*).

## 4. Discussion

This study provides a multiscale characterization of the mechanisms modulating the spatiotemporal dynamics of early and long-duration VF episodes. The main insights are: (i) early VF dynamics are independent of a pre-existing infarct-related substrate and are characterized by consistently higher ARs in RV regions; (ii) AR gradients consolidate and become more pronounced across right-to-left and epicardial-to-endocardial axes during LDVF episodes, with the LV showing earlier electrical depression than the RV, suggesting asymmetric ventricular vulnerability to global ischaemia; (iii) interventricular differences in baseline APD and in the maximal conductance of K_ATP_ channels do not account for the right-to-left AR gradients documented during VF; (iv) metabolism-related biomarkers in blood samples from epicardial RV and LV veins support a greater RV resilience to VF-induced global ischaemia; (v) computational modelling confirmed that the RV maintained higher excitability than the LV under the ischaemic conditions of a VF event, which explains the right-to-left AR gradients documented experimentally *in vivo* and *ex vivo*; (vi) in humans, VF ARs show similar patterns to those documented in pigs; and (vii) VF ARs derived from surface ECG tracings prior to the first DC shock can predict neurological outcomes in comatose patients admitted after out-of-hospital cardiac arrest.

This provides novel insights into the asymmetric dynamics of the RV and LV and the mechanisms modulating AR patterns during VF. Previous series that performed invasive or non-invasive simultaneous mapping of both ventricles did not report RV-to-LV gradients comparable to our data.^3,16,17^ However, those studies only analysed SDVF episodes (∼10–20 s), and focused mostly on wave activation patterns rather than regional heterogeneities in ARs. Some studies focused on LDVF have also shown a similar time-course evolution of overall ventricular ARs in a variety of myocardial substrates.^18^ Others authors reported regional heterogeneities in ARs across the ventricular walls but disagreed on the direction of the gradients or on their underlying mechanisms. Thus, some studies indicated that Purkinje fibres play an important role during SDVF,^3^ and potentially also during LDVF.^17,19^ However, others reported that Purkinje fibres may contribute to, but do not fully explain, the formation of AR gradients during LDVF.^7,20^

Our data show that higher VF ARs in the RV than in the LV are indicative of a better tolerance within the RV to the global ischaemia of a VF event. Metabolic parameters measured during the course of LDVF, and computational simulations incorporating experimentally derived measurements, indicated that a differential RV-LV response to ischaemia ultimately explains lower ARs and earlier electrical depression in the LV. Interestingly, our data showed a lack of baseline APD differences between the LV and the RV, and a greater pinacidil-induced K_ATP_-channel conductance in the LV, in line with previous reports.^21^ However, our results indicated that RV-to-LV AR gradients during VF are primarily driven by differences in acidosis and extracellular K ^+^ . These factors decrease excitability to a larger degree in the LV than in the RV, likely extending the LV’s effective refractory period beyond APD (a phenomenon known as postrepolarization refractoriness)^22^ and limiting the LV increase in ARs.

Our findings align with a recent study showing that dynamic functional adaptations may remain inconspicuous at baseline and only emerge during metabolic stress.^23^ Thus, in our study, AR rate gradients during VF are not explained by baseline structural or functional differences, but arise over the course of VF-associated ischaemia. The differences between the LV and RV in NADH and lactate accumulation support an early energy-collapse in the LV, whilst a sustained shift towards anaerobic energy production helped preserve cellular homeostasis longer in the RV.^24,25^ Prior studies have shown that the RV has the ability to downregulate its metabolic demands during coronary hypoperfusion, thereby maintaining energy stores and conferring additional protection against ischaemia.^26^ Factors such as lower baseline pressure, thinner walls, and reduced stiffness may allow the RV to enhance oxygen-uptake efficiency during hypoxia,^27^ which likely contributes to sustaining RV-to-LV AR gradients during VF. Similarly, the observed epicardium-to-endocardium AR gradients may also reflect a greater endocardial vulnerability to ischaemia,^28^ consistent with larger infarct sizes in the endocardium after MI.^29^ Interestingly, the AR hierarchies documented during VF mirror the extent of MI scarring after coronary occlusion. Thus, the slowest-activating LV (sub)endocardium shows the highest infarction risk, whereas the fastest-activating RV typically shows functional recovery after ischaemia.^30^

The results also support the notion that VF ARs may serve in clinical scenarios as a dynamic marker of cardiac status and, indirectly, as an indicator of potential damage to sensitive organs such as the brain. Specifically, lower ARs on VF tracings prior to DC shock indicate a higher likelihood of severe brain injury, likely reflecting more advanced stages of ischaemia resulting from the prolonged absence of effective perfusion during VF. This is consistent with the predictive value of F_median_ analysis on ECG tracings prior to DC shock in comatose survivors admitted to the hospital after VF-induced cardiac arrest. These findings align with previous studies highlighting the value of VF parameters derived from waveform analysis for predicting defibrillation success and survival.^31^

We acknowledge some limitations. First, the analyses focused on regional and global ventricular ARs to describe VF dynamics and did not assess their correlation with focal or re-entrant waveform patterns. Second, whole-cell patch-clamp experiments did not explore potential differences in other relevant currents, such as *I*_Na_. Moreover, the cell isolation of individual cardiomyocytes did not allow us to distinguish between endocardial and epicardial myocytes. Third, we did not explore the underlying mechanisms behind transmural gradients in ARs. Different transmural distributions of Purkinje fibres have been reported across species, which may play a role in the direction of endo-epicardial AR gradients.^19^ Notwithstanding, some discrepancies in the reported directions of transmural gradients across studies might be explained by methodological limitations (e.g. catheter electrodes vs. transmural plunge needle electrodes). Fourth, despite similar AR patterns, our findings in pigs provide mechanistic plausibility rather than direct equivalence for clinical observations. Our study did not address interventricular asymmetries in ARs and myocardial resilience to global ischaemia in patients. Species-specific differences in myocardial structure, pre-existing remodelling, and differences in the Purkinje network between humans and pigs might affect the mechanistic insights derived from pig data. Fifth, we did not assess the role of the Purkinje system, although faster ARs within Purkinje fibres^19^ would align with our findings, given their greater resistance to ischaemia.^32^ However, high-frequency activations from Purkinje fibres might not propagate into electrically depressed myocardium, remaining undetected when measuring myocardial ARs. Finally, we did not explore therapeutic strategies to abolish AR asymmetries and their potential effect on favouring defibrillation. Farid *et al.*^33^ showed that homogenization of cycle lengths via *I*_KATP_ blockade favoured VF termination in isolated Langendorff-perfused human hearts from transplant recipients with dilated cardiomyopathy. However, other studies have reported that I_KATP_ blockade may impair the channel’s cardioprotective function and worsen the energy demand-supply imbalance during ischaemia.^34^

In conclusion, regional activation rates during VF are directly modulated by asymmetric myocardial tolerance to factors associated with global ischaemia during cardiac arrest. Fast activation rates reflect early VF stages, metabolic resilience, and preserved myocardial excitability which, in clinical scenarios, are predictive of favourable neurological outcome in comatose survivors of cardiac arrest.

Translational perspective VF is the most lethal cardiac arrhythmia, causing global ischaemia and leading to death within a few minutes. Here, we demonstrate that interventricular gradients in endocardial/epicardial activation rates (AR) during VF reflect an asymmetric myocardial tolerance to global ischaemia in the pig. Similar AR patterns were documented in surface ECG tracings, where higher ARs predicted favourable neurological outcomes in patients experiencing VF-related cardiac arrest. The results highlight that ventricular ARs during VF are a dynamic marker of ischaemia-induced damage both in the heart and critical organs relying on its effective pumping function. This may guide resuscitation efforts and improve prognostication.

## Supplementary Material

cvag101_Supplementary_Data

## Data Availability

The data and custom-written code that support the findings of this study are available from the corresponding authors upon reasonable request. Full details of the novel analytical methods used in this article are fully disclosed in the article and the Supplementary material.
