# Supplementary material for: Ventricular fibrillation dynamics reveal regional asymmetry in resilience to cardiac arrest and predict clinical outcome
Source: Cardiovasc Res. 2026 May 28;122(9):1191–205. doi: 10.1093/cvr/cvag101 (PMC13307562; doi:10.1093/cvr/cvag101)
Supplement: cvag101_Supplementary_Data [file cvag101_supplementary_data.zip › Supplementary_Material_R1.pdf]

SUPPLEMENTARY DATA

Ventricular Fibrillation Dynamics Reveal Regional Asymmetry in Resilience to Cardiac Arrest and Predict Clinical Outcome

Authors: Andrés Redondo-Rodríguez[1,2], Jorge G. Quintanilla[1,2,3]*, Álvaro Macías[4], Peter Lee[5], David Calvo[2,3], Marinela Couselo-Seijas[1,2], Manuel Marina-Breysse[1,2], Ana Simón-Chica[1], Alba García-Escolano[1], Alba Ramos-Prada[1,6], José Manuel Alfonso-Almazán[1], Jesús Diz-Díaz[1,7,8], Laura Gil-Martínez[9], Sergio Muñoz-Romero[10], Carlos Galán-Arriola[2,11], Javier Sánchez-González[12], Juan José González-Ferrer[2,3], Victoria Cañadas-Godoy[2,3], Ricardo Salgado-Aranda[3], Alba Cruz-Galbán[3], María Jesús García-Torrent[2,3,13], José Luis Rojo-Álvarez[10], Javier Saiz[9], Borja Ibañez[2,8,11], Julián Pérez-Villacastín[2,3,6], Nicasio Pérez-Castellano[2,3,6], José Jalife[2,4], José M. Ferrero[9], David Filgueiras-Rama[1,2,3]*

Affiliations:

[1]Centro Nacional de Investigaciones Cardiovasculares (CNIC), Advanced Development in Arrhythmia Mechanisms and Therapy Laboratory, Novel Arrhythmogenic Mechanisms Program; Madrid, Spain

[2]Centro de Investigación Biomédica en Red de Enfermedades Cardiovasculares (CIBERCV); Madrid, Spain

[3]Instituto de Investigación Sanitaria del Hospital Clínico San Carlos (IdISSC), Cardiovascular Institute; Madrid, Spain.

[4]Centro Nacional de Investigaciones Cardiovasculares (CNIC), Cardiac Arrhythmia Laboratory, Cardiovascular Regeneration Program; Madrid, Spain

[5]Essel Research and Development Inc.; Toronto, Canada.

[6]Fundación Interhospitalaria para la Investigación Cardiovascular; Madrid, Spain

[7]Cardiology Department, Hospital Universitario La Paz; Madrid, Spain

[8]Cardiology Department, Instituto de Investigación Sanitaria Fundación Jiménez Díaz; Madrid, Spain

[9]Centro de Investigación e Innovación en Bioingeniería (Ci2B), Universitat Politècnica de València; Valencia, Spain.

[10]Departamento de Teoría de la Señal y Comunicaciones y Sistemas Telemáticos y Computación, Universidad Rey Juan Carlos; Madrid, Spain

[11]Centro Nacional de Investigaciones Cardiovasculares (CNIC), Translational Laboratory for Cardiovascular Imaging and Therapy, Myocardial Homeostasis & Cardiac Injury Program; Madrid, Spain

[12]Philips Healthcare Iberia; Madrid, Spain

[13]Universidad Complutense de Madrid; Madrid, Spain

***Corresponding authors:** Jorge G. Quintanilla & David Filgueiras-Rama.
Email: jgquintanilla@cnic.es; Email: david.filgueiras@cnic.es.

SUPPLEMENTARY TABLES

Table S1A. Pigs characteristics for *in vivo* protocols

	**Early-VF protocol**		**Long-duration-VF protocol**	
	eMI (N=6)	Healthy (N=6)	eMI (N=6)	Healthy (N=5)
Female, n (%)	4 (66.7)	1 (16.7)	0 (0)	0 (0)
Age, months	7 (6, 9)	6 (4, 6)	5 (5, 6)	5 (4, 6)
Weight, kg	79 (75, 97)	74 (61, 79)	58 (50, 62)	55 (51, 65)
Time post-MI, weeks	16 (12, 23)	N/A	9 (9, 10)	N/A

Data are expressed as median and interquartile ranges and n (%), as appropriate. eMI: established myocardial infarction. MI: myocardial infarction. VF: ventricular fibrillation

Table S1B. Pigs characteristics for *ex vivo* protocols

	**Panoramic OM protocol**		**Ratiometric OM protocol**	
	eMI (N=6)	Healthy (N=6)	eMI (N=0)	Healthy (N=7)
Female, n (%)	1 (16.7)	0 (0)	-	2 (28.6)
Age, months	5 (4, 7)	4 (4, 5)	-	7 (7, 9)
Weight, kg	65 (54, 89)	55 (53, 69)	-	68 (61, 72)
Time post-MI, weeks	10 (6, 15)	N/A	-	N/A

Data are expressed as median and interquartile ranges and n (%), as appropriate. eMI: established myocardial infarction. MI: myocardial infarction. OM: optical mapping

Table S1C. Pigs characteristics for *in vitro* protocols

	**K_{ATP} gene/protein expression**		**Whole-cell patch clamp**	
	eMI (N=7)	Healthy (N=5)	eMI (N=0)	Healthy (N=6)
Female, n (%)	2 (28.6)	1 (20.0)	-	3 (50.0)
Age, months	10 (10, 12)	6 (6, 6)	-	5 (4, 6)
Weight, kg	122 (114, 140)	88 (77, 96)	-	51 (46, 61)
Time post-MI, weeks	31 (28, 39)	N/A	-	N/A

Data are expressed as median and interquartile ranges and n (%), as appropriate. eMI: established myocardial infarction. K_{ATP}: ATP-sensitive inward rectifier potassium current channels. MI: myocardial infarction.

Table S1D. Pigs characteristics for open-chest protocols

	***In vivo* NADH protocol**		**Coronary-vein blood protocol**	
	eMI (N=0)	Healthy (N=5)	eMI (N=0)	Healthy (N=7)
Female, n (%)	-	0 (0)	-	5 (71.4)
Age, months	-	7 (5, 7)	-	8 (8, 9)
Weight, kg	-	70 (67, 75)	-	92 (89, 115)
Time post-MI, weeks	-	N/A	-	N/A

Data are expressed as median and interquartile ranges and n (%), as appropriate. eMI: established myocardial infarction. MI: myocardial infarction.

Table S2. Baseline characteristics of the retrospective series of patients with VF-induced cardiac arrest.

**Patients from Group 2 (N=60)**	
Clinical variables	
Age (years)	59 (47, 68)
Female, n (%)	7 (11.7)
Hypertension, n (%)	28 (46.7)
Diabetes, n (%)	10 (16.7)
Dyslipidaemia, n (%)	22 (36.7)
Atrial fibrillation, n (%)	11 (18.3)
Smoking habit, n (%)	25 (41.7)
Previous MI, n (%)	15 (25.0)
Previous revascularization, n (%)	7 (11.7)
Previous stroke, n (%)	2 (3.3)
Chronic renal failure, n (%)	2 (3.3)
DCM, n (%)	2 (3.3)
COPD, n (%)	8 (13.3)
Severe valvulopathy, n (%)	2 (3.3)
Heart failure, n (%)	14 (23.3)
Family history of SCD, n (%)	10 (16.7)
Acquired LQTS, n (%)	1 (1.7)
Brugada syndrome, n (%)	1 (1.7)
Preexcitation, n (%)	1 (1.7)
Congenital LQTS, n (%)	1 (1.7)
HCM, n (%)	3 (5.0)
Background treatment	
Betablockers, n (%)	16 (26.7)
Aspirin, n (%)	9 (15.0)
Thienopyridines, n (%)	1 (1.7)
Amiodarone, n (%)	2 (3.3)
Calcium antagonists, n (%)	3 (5.0)
Statins, n (%)	18 (30.0)
ACE inhibitors, n (%)	13 (21.7)
ARBs, n (%)	4 (6.7)
Diuretics, n (%)	14 (23.3)
Aldosterone inhibitors, n (%)	3 (5.0)
Anticoagulants, n (%)	12 (20.0)
Cardiac arrest variables	
Number of shocks delivered before ROSC	3 (2, 5)
Time to ALS (min)	8 (5, 11)
Time performing ALS (min)	14 (6, 20)
Post-ROSC reduced LVEF, n (%)	20 (33.3)

Data are expressed as median and interquartile ranges and n (%) as appropriate. ACE: angiotensin-converting enzyme. ALS: advanced life support. ARBs: angiotensin II receptor blockers. COPD: chronic obstructive pulmonary disease. DCM: dilated cardiomyopathy. HCM: hypertrophic cardiomyopathy. LVEF: left ventricle ejection fraction. LQTS: long-QT syndrome. MI: myocardial infarction. ROSC: return of spontaneous circulation. SCD: sudden cardiac death.

Table S3. Univariate analysis of clinical characteristics of the retrospective series of patients with VF-induced cardiac arrest.

**Patients from Group 2 (N=60)**					
	**non-FNP (n=23)**	**FNP (n=37)**	**p-value**	**OR (CI 95%)**	**AUC (CI 95%)**
Clinical variables					
Age (years)	65 (50, 72)	56 (42, 65)	**0.032**	0.955 (0.916, 0.996)	0.659 (0.519, 0.799)
Female, n (%)	4 (17.4)	3 (8.1)	0.286		
Hypertension, n (%)	10 (43.5)	18 (48.6)	0.696		
Diabetes, n (%)	6 (26.1)	4 (10.8)	0.133		
Dyslipidaemia, n (%)	9 (39.1)	13 (35.1)	0.755		
Atrial fibrillation, n (%)	7 (30.4)	4 (10.8)	0.066		
Smoking habit, n (%)	9 (39.1)	16 (43.2)	0.753		
Previous MI, n (%)	8 (34.8)	7 (18.9)	0.173		
Previous revascularization, n (%)	4 (17.4)	3 (8.1)	0.523		
Previous stroke, n (%)	1 (4.3)	1 (2.7)	0.732		
Chronic renal failure, n (%)	1 (4.3)	1 (2.7)	0.732		
DCM, n (%)	2 (8.7)	0 (0)	>0.999		
COPD, n (%)	3 (13.0)	5 (13.5)	0.958		
Severe valvulopathy, n (%)	1 (4.3)	2 (5.4)	0.855		
Heart failure, n (%)	9 (39.1)	5 (13.5)	**0.028**	0.243 (0.069, 0.858)	0.628 (0.477, 0.779)
Family history of SCD, n (%)	3 (13.0)	7 (18.9)	0.772		
Acquired LQTS, n (%)	0 (0)	1 (2.7)	>0.999		
Brugada syndrome, n (%)	0 (0)	1 (2.7)	>0.999		
Preexcitation, n (%)	0 (0)	1 (2.7)	>0.999		
Congenital LQTS, n (%)	0 (0)	1 (2.7)	>0.999		
HCM, n (%)	1 (4.3)	2 (5.4)	0.855		
Background treatment					
Betablockers, n (%)	5 (21.7)	11 (29.7)	0.498		
Aspirin, n (%)	3 (13.0)	6 (16.2)	0.738		
Thienopyridines, n (%)	0 (0)	1 (2.7)	>0.999		
Amiodarone, n (%)	2 (8.7)	0 (0)	>0.999		
Calcium antagonists, n (%)	2 (8.7)	1 (2.7)	0.326		
Statins, n (%)	6 (26.1)	12 (32.4)	0.603		
ACE inhibitors, n (%)	3 (13.0)	10 (27.0)	0.210		
ARBs, n (%)	3 (13.0)	1 (2.7)	0.155		
Diuretics, n (%)	6 (26.1)	8 (21.6)	0.691		
Aldosterone inhibitors, n (%)	1 (4.3)	2 (5.4)	0.855		
Anticoagulants, n (%)	7 (30.4)	5 (13.5)	0.119		
Cardiac arrest variables					
N shocks before ROSC	5 (2, 7)	3 (2, 4)	**0.016**	0.760 (0.608, 0.951)	0.704 (0.564, 0.844)
Time to ALS (min)	10 (8, 15)	5 (4, 10)	**0.004**	0.850 (0.759, 0.950)	0.757 (0.632, 0.882)
Time performing ALS (min)	16 (13, 38)	8 (4, 16)	**0.014**	0.953 (0.916, 0.990)	0.752 (0.631, 0.872)
Post-ROSC reduced LVEF, n (%)	10 (43.5)	10 (27.0)	0.192		
Spectral analysis					
Median Frequency (Hz)	3.2 (2.9, 3.9)	5.5 (4.6, 6.1)	**<0.001**	9.316 (3.030, 28.65)	0.937 (0.875, 0.998)
Dominant Frequency (Hz)	3.2 (2.9, 4.0)	5.5 (4.4, 6.5)	**<0.001**	5.905 (2.405, 14.50)	0.920 (0.846, 0.995)
VF segment duration (s)	3.0 (2.9, 5.5)	3.9 (2.9, 5.4)	0.618		

Data are expressed as median and interquartile ranges and n (%) as appropriate. Statistical comparisons between patients with and without favourable neurological performance (FNP) were performed using a univariate logistic regression analysis. In variables with $p<0.05$, odds ratio (OR) and area under the curve (AUC) were calculated. VF: ventricular fibrillation. Other abbreviations as in Table S2.

Table S4. Gene primers (*sus scrofa*) used to amplify cDNA in RT-qPCR

Gene	Forward primer	Reverse primer
GAPDH	CCATCTTCCAGGAGCGAGAT	AGAAGGGGCAGAGATGATGA
K_{ir}6.1 (KCNJ8)	GGTTTGGAGTCCACTGTGTGTGTGA	GGGCATTCCTCTGTCATCATCCTC
K_{ir}6.2 (KCNJ11)	TGATGAGGACCACAGCCTACTGGA	AGGACAGGGAATCTGGAGAGATGCT
SUR1 (ABCC8)	TGCCGCACGTCTTCCTACTCTTCA	CAGGATGCCCTCTGCAATCTCACA
SUR2A (ABCC9)	ATGAGGCAACAGCTTCCATTGACA	AAGACTAAAACAAGGCCTGCATCCA
SUR2B (ABCC9)	GCTTCCATTGACATGGCCACAGA	GCCAAGAGGCTTTCTGGAGTGTCA

GAPDH: Glyceraldehyde-3-phosphate dehydrogenase. K_{ir}6.1: ATP-sensitive inward rectifier potassium channel 8. K_{ir}6.2: ATP-sensitive inward rectifier potassium channel 11. SUR2A: Sulfonylurea receptor 2A (ATP-binding cassette, sub-family C, member 9, variant A). SUR2B: Sulfonylurea receptor 2B (ATP-binding cassette, sub-family C, member 9, variant B).

Table S5. Primary and secondary antibodies used in Western Blots

Target antigen	Working concentration	Catalog #	Vendor
K_{ir}6.1 (KCNJ8)	1:200	APC-105	Alomone labs
K_{ir}6.2 (KCNJ11)	1:200	APC-020	Alomone labs
SUR2A (ABCC9)	1:1000	OASE00279	Aviva
SUR2B (ABCC9)	1:1000	MABn511	Merck Millipore
Polyclonal Goat Anti-Mouse (iG/HRP)	1:10000	P044701-2	Dako
Goat Anti-Rabbit (iG/HRP)	1:10000	P044801-2	Dako

K_{ir}6.1: ATP-sensitive inward rectifier potassium channel 8. K_{ir}6.2: ATP-sensitive inward rectifier potassium channel 11. SUR2A: Sulfonylurea receptor 2A (ATP-binding cassette, sub-family C, member 9, variant A). SUR2B: Sulfonylurea receptor 2B (ATP-binding cassette, sub-family C, member 9, variant B).

SUPPLEMENTARY FIGURES

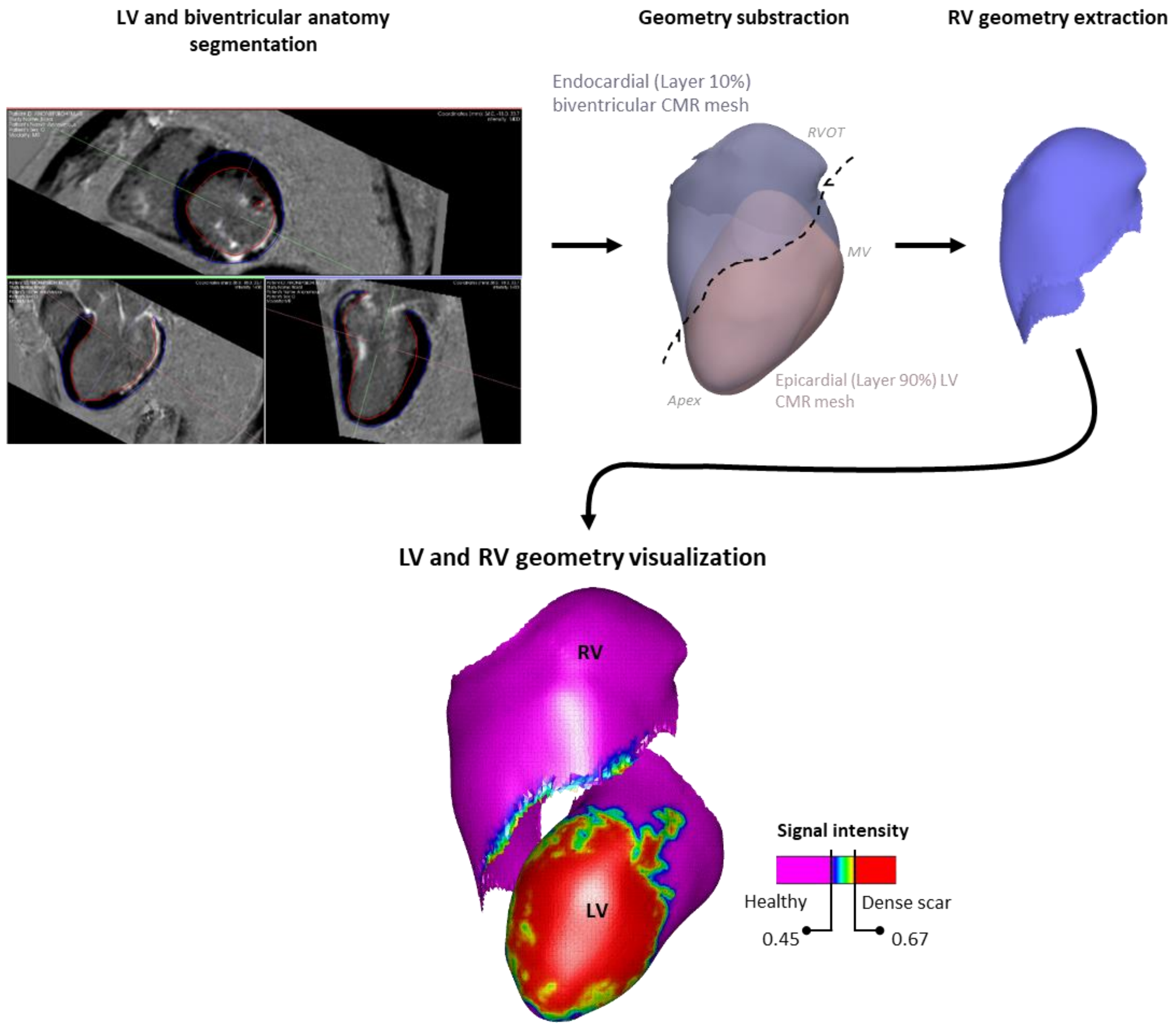

Figure S1. Schematic workflow of cardiac magnetic resonance imaging segmentation. Late-gadolinium enhancement cardiac magnetic resonance (LGE-CMR) images were used to characterize the infarct-related substrate in animals with established myocardial infarction. Segmentations of left ventricular (LV) and biventricular anatomies were performed using the ADAS3D software (top left). The epicardial layer of the LV segmentation was subtracted from endocardial layer of the biventricular geometry to obtain an endocardial right ventricular (RV) geometry (top right). The resulting geometries were used to characterize the infarct-related substrate of both ventricles using 0.45 and 0.67 normalized signal intensity criteria for heterogeneous and dense scar, respectively (bottom row).

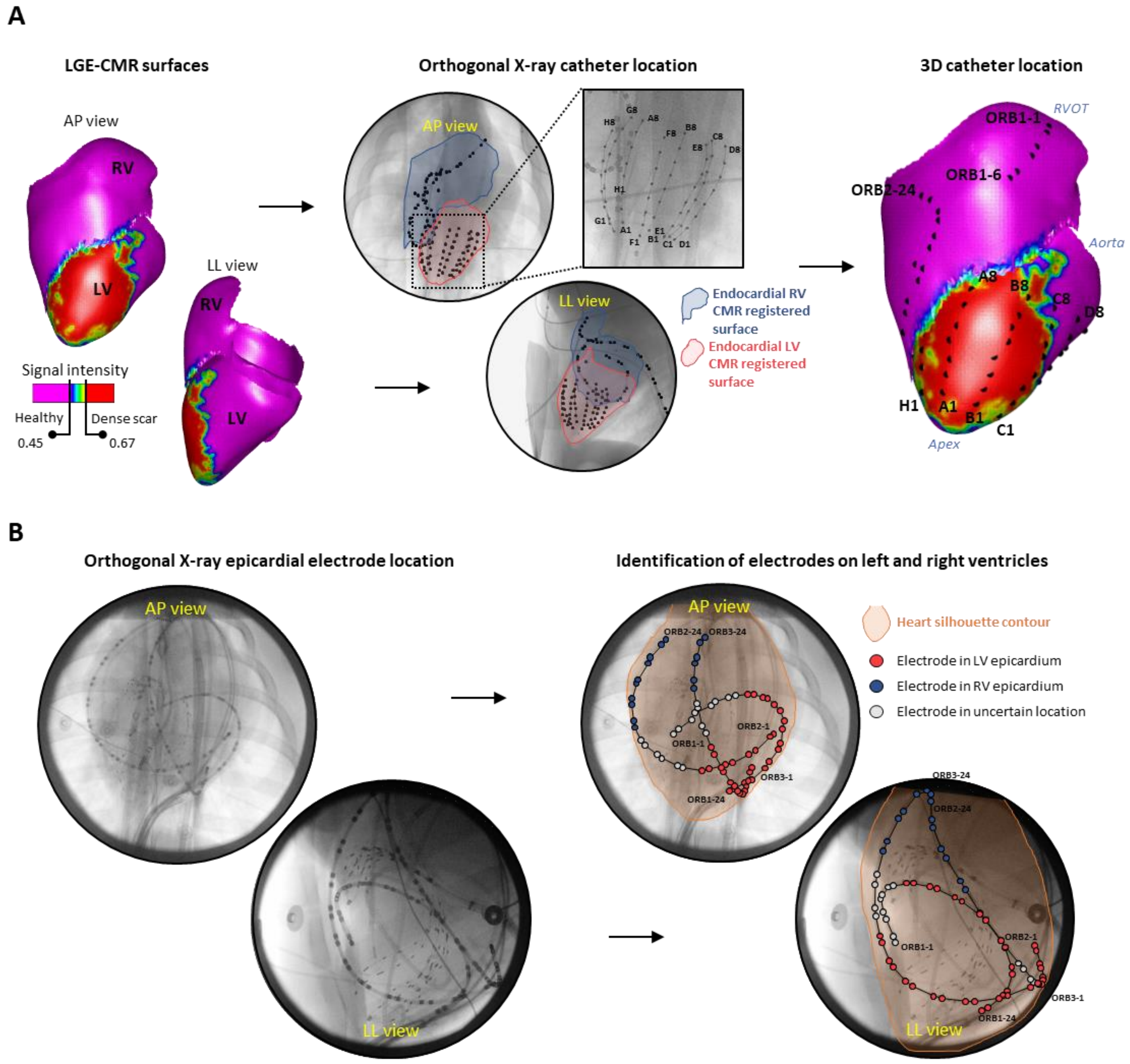

Figure S2. Schematic workflow of catheter location on ventricular surfaces in the three-dimensional space. A Late-gadolinium enhancement cardiac magnetic resonance (LGE-CMR) images were used to characterize the infarct-related substrate using a 0.45 and 0.67 normalized-signal-criteria for heterogeneous and dense scar, respectively (left column). Catheters positioned inside the heart during the electrophysiological study were identified in orthogonal fluoroscopic images (antero-posterior [AP] and left-lateral [LL] views) and registered with the contours of their corresponding CMR geometries (central column). Catheter electrodes are highlighted with black dots and linearly connected. A zoom-in of the basket catheter is depicted for the AP view, tagging proximal and distal electrodes of each spline (A-H). Catheter electrodes were projected onto their corresponding geometry using a minimum-total-distance criterion, (right column). **B** Identification of catheter electrodes placed on the epicardium. Left, sample orthogonal fluoroscopic heart views showing basket-catheters placed endocardially in the LV and RV and three 24-pole catheters located on the epicardium. Right, 24-pole-catheters electrodes were manually assigned to the epicardium of the LV (red dots) or the RV (blue dots). Electrodes located close to interventricular grooves were not assigned to any ventricle (white dots). Manually-delineated heart contours on the fluoroscopic images are highlighted in orange. RVOT: RV outflow tract.

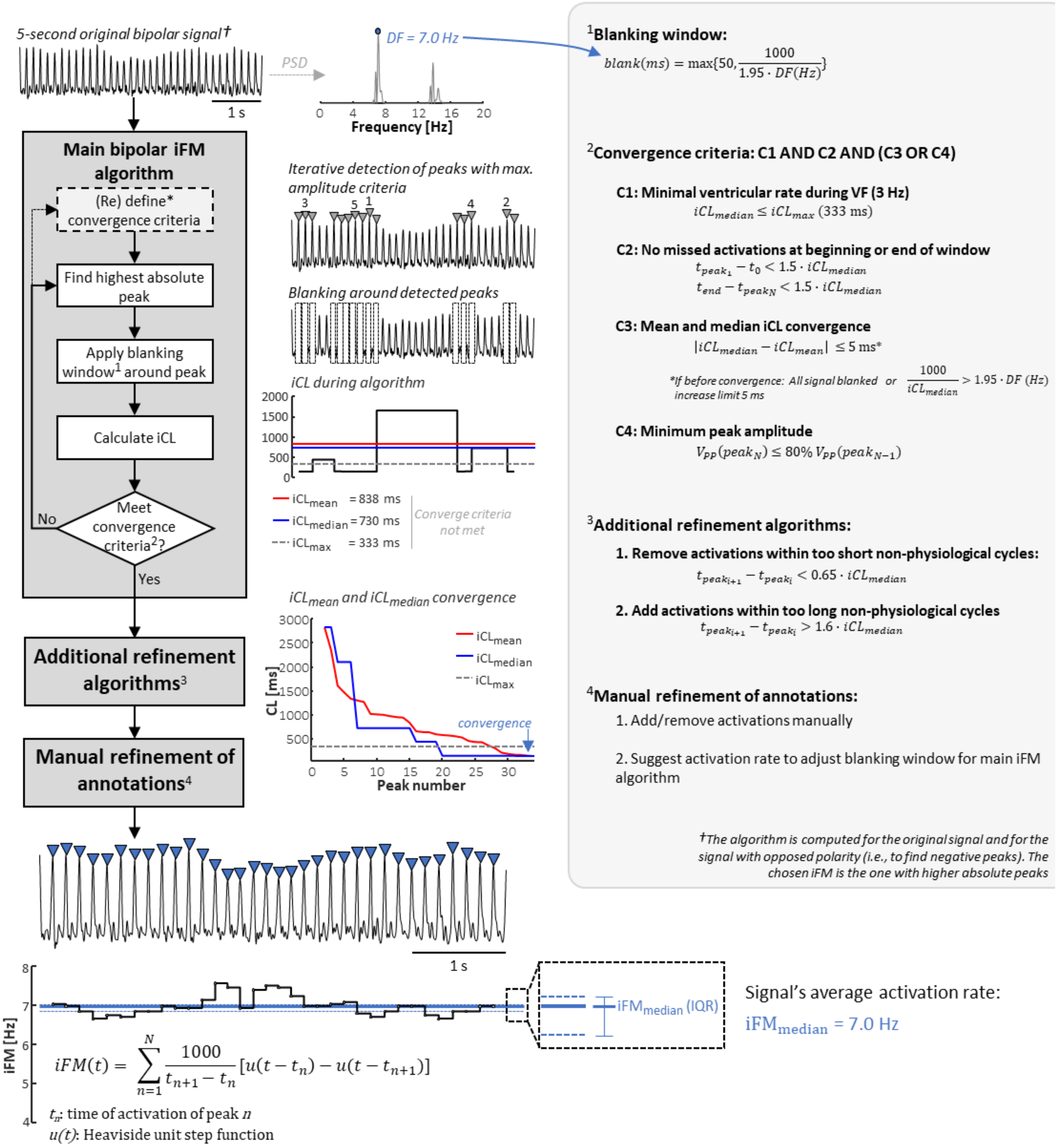

Figure S3. Schematic workflow describing the steps of instantaneous frequency modulation analysis of bipolar signals. DF: dominant frequency. iCL: instantaneous cycle length. iFM: instantaneous frequency modulation. IQR: interquartile range. PSD: power spectral density. Vpp: peak-to-peak voltage.

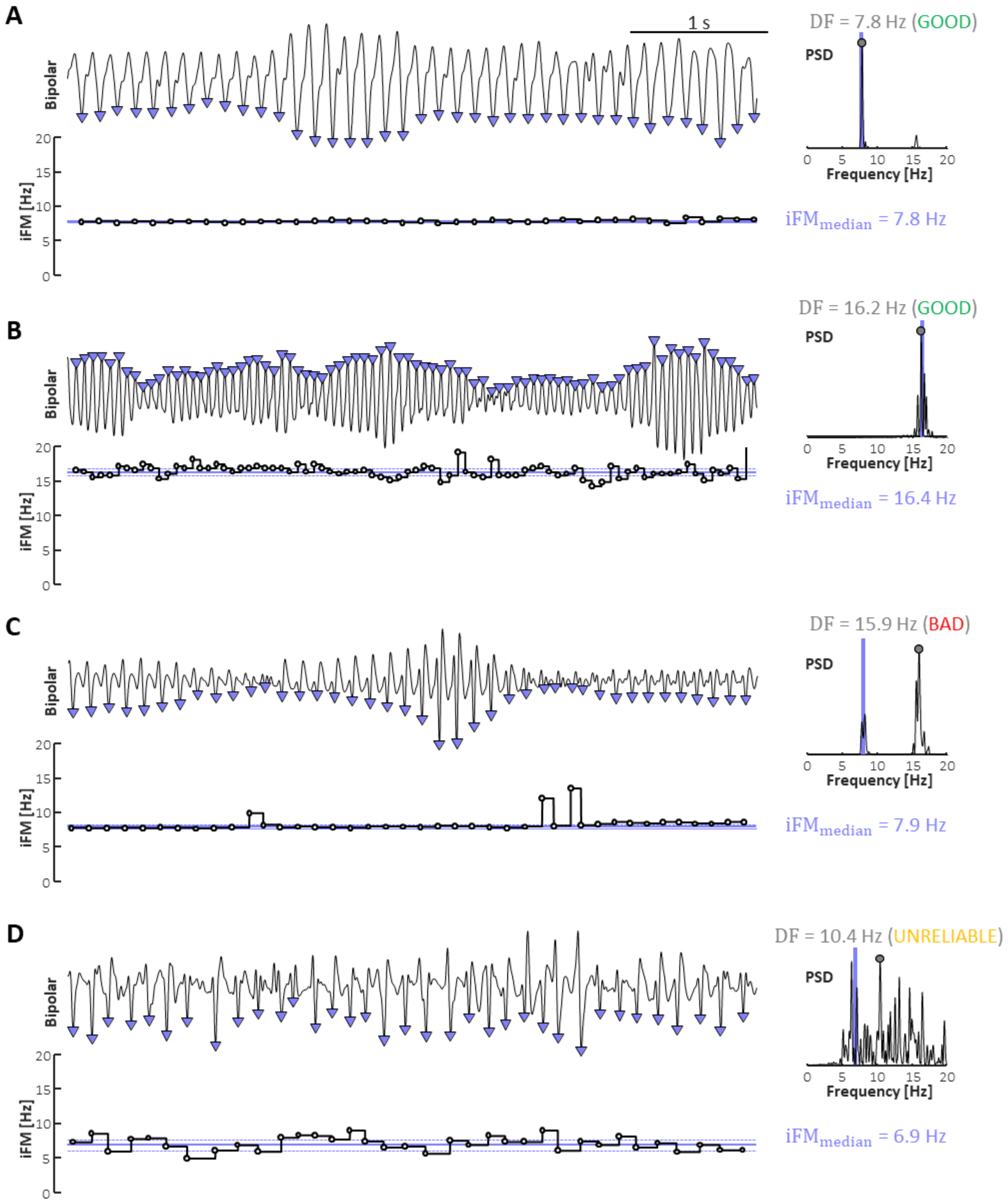

Figure S4. Comparison between instantaneous frequency modulation and dominant frequency analyses in sample fibrillatory bipolar signals. Signals correspond to different ventricular regions at different timepoints of the same ventricular fibrillation episode. For each panel, the first row shows the bipolar signal with blue triangles indicating the annotated activation times using the instantaneous frequency modulation (iFM) algorithm (left), and the power spectral density (PSD) with the dominant frequency (DF; the frequency of the highest peak in the PSD) (right). The bottom row shows the resulting iFM signal. The iFM$_{median}$ and the interquartile iFM are shown with blue thick and dashed lines, respectively. The iFM$_{median}$ is also shown in the PSD with a blue vertical line. **A, B,** Sample signals with a good correlation between iFM$_{median}$ and DF at variable physiological activation rates (~8 Hz in **A**, ~16 Hz in **B**). **C,** Signal with poor correlation between DF and iFM$_{median}$ because the second harmonic was selected as DF. **D,** Signal with poor correlation between DF and iFM$_{median}$ because the PSD displays multiple peaks of similar height, which limits DF analysis to accurately estimate local activation rates.

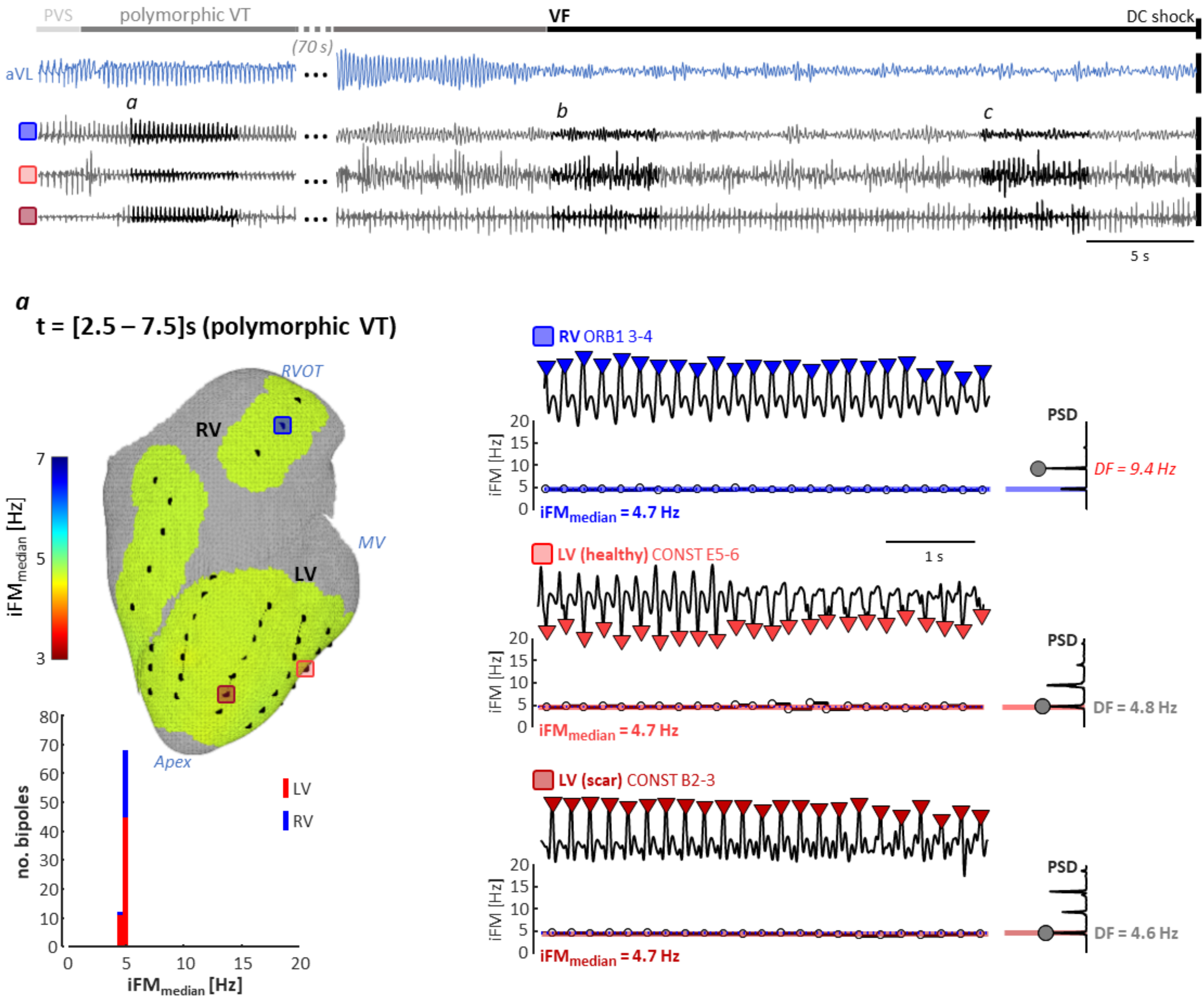

Figure S5. Sample instantaneous frequency modulation analysis of bipolar signals from an *in vivo* ventricular fibrillation episode. Top row shows the programmed ventricular stimulation (PVS) protocol that induced a polymorphic ventricular tachycardia (VT) which finally self-degenerated into ventricular fibrillation (VF). The episode was terminated with a DC shock after 30 seconds in VF. A surface ECG lead (aVL) is shown in blue, on top of sample electrograms (in grey) from intracardiac electrodes. A zoom-in to significant timepoints of the ventricular arrhythmia episode coded with letters *a-c* is shown below. In each panel: Left, the 3D color-coded median instantaneous frequency modulation (iFM_{median}) map. Visible bipoles from the catheters inside the left and right ventricles (LV and RV, respectively) are marked with black dots. A color-coded histogram of all iFM_{median} values is shown below the 3D map. Right, iFM analysis of sample bipoles marked with coloured squares in the 3D map. For each case, the top row shows the bipolar signal with triangles in the detected activation times. The bottom row shows the resulting iFM signal and the iFM_{median} value, along with the power spectral density (PSD) and the dominant frequency (DF) annotation. (**a**) Early window of the induced polymorphic VT. The histogram during polymorphic VT shows that all the endocardium is activating at the same rate. MV: mitral valve. RVOT: RV outflow tract.

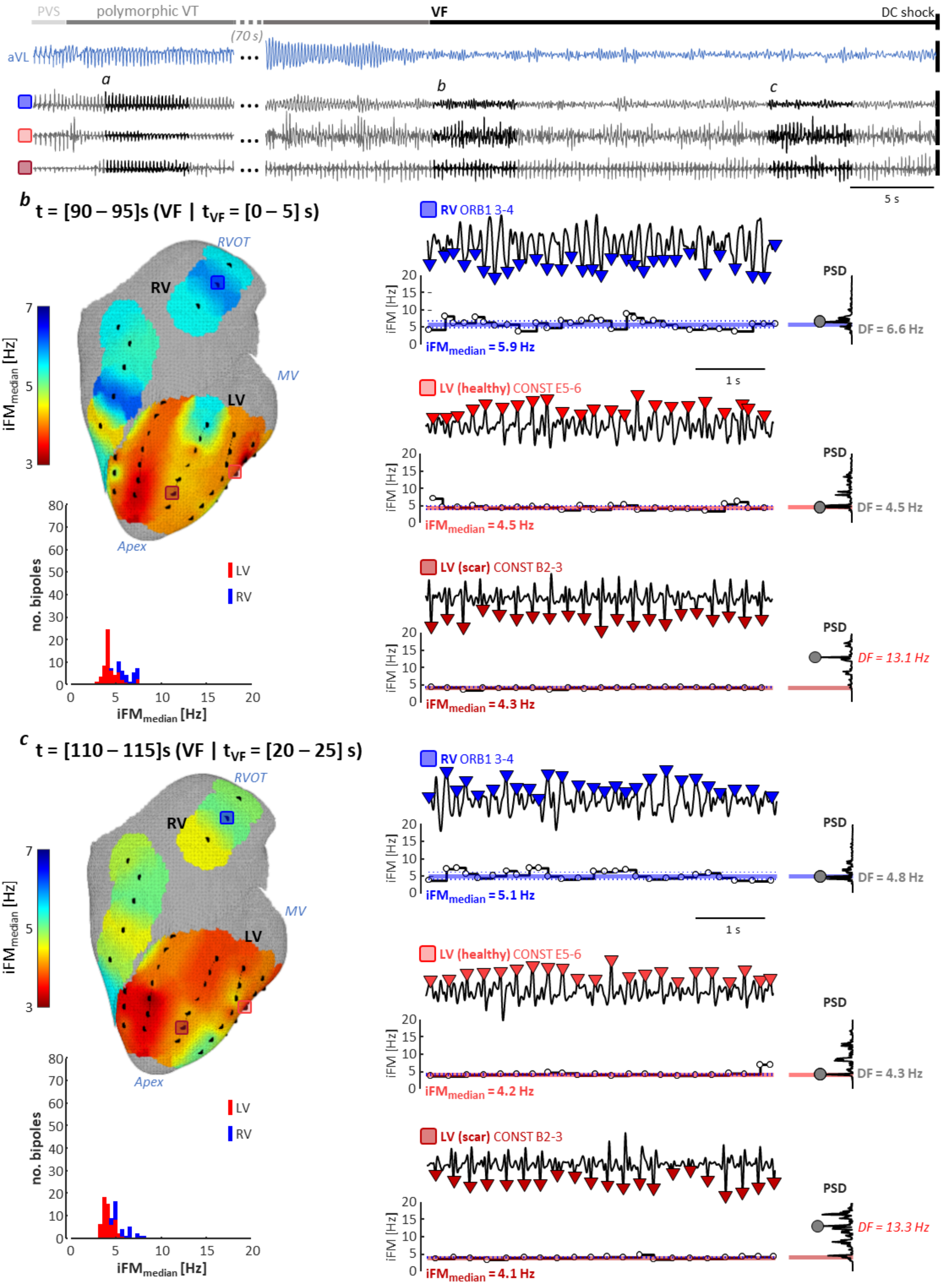

Figure S5 (continued). Sample instantaneous frequency modulation analysis of bipolar signals from an in vivo ventricular fibrillation episode. (b) Initial VF window. **(c)** [20-25] s window of the VF episode. Histograms show that during temporal windows (**b**) and (**c**) the RV is activating faster than the LV. MV: mitral valve. RVOT: RV outflow tract.

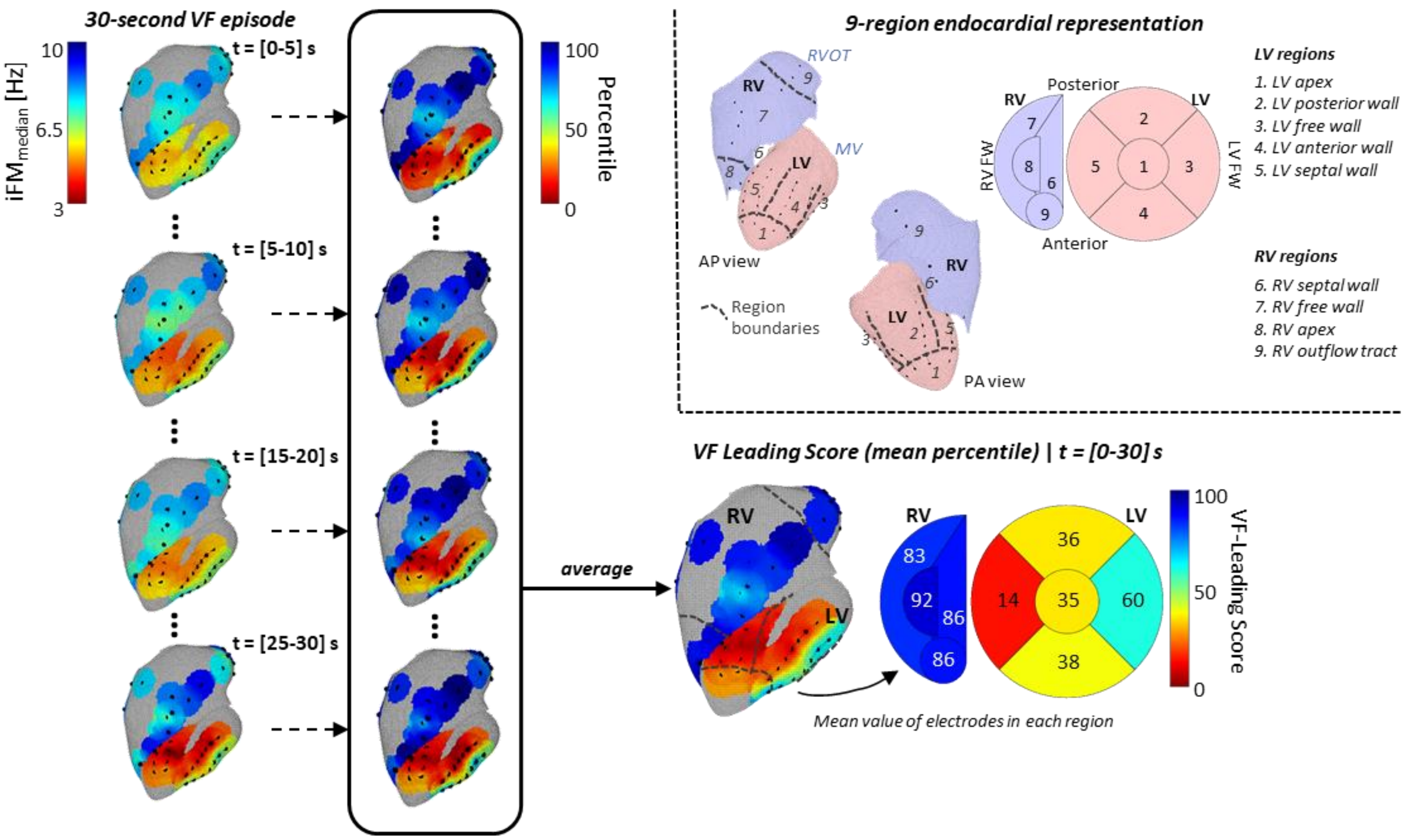

Figure S6. Ventricular fibrillation leading score analysis of *in vivo* endocardial electrograms. Left-most column, median instantaneous frequency modulation (iFM_{median}) maps of the endocardium in 5-second windows during the first 30 seconds of a ventricular fibrillation (VF) episode. Second-most left column shows the percentile values of the iFM_{median} calculated in each window. For each bipole, the iFM_{median} percentile is averaged through all the temporal windows to yield the VF-leading score (bottom right). Scores from the electrodes in each region of the 9-region endocardial division (upper right) are averaged to obtain a single VF score value per region that is displayed in a schematic endocardial representation (bottom right) that allows for inter-case comparisons.

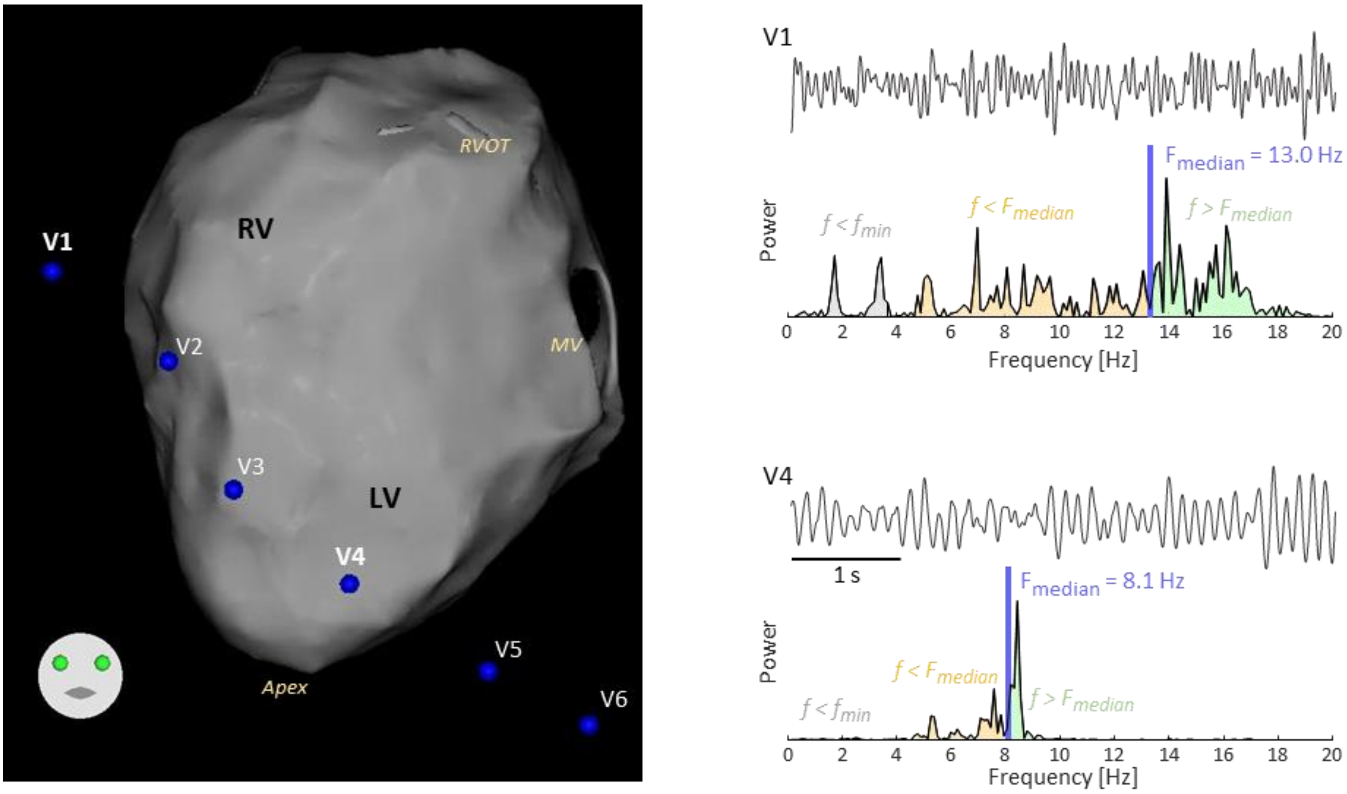

Figure S7. Median frequency analysis in surface ECG tracings during *in vivo* ventricular fibrillation in pigs. Left, spatial location of the six precordial ECG-leads (blue circles) relative to the epicardial geometry of the left and right ventricles (LV and RV, respectively). LV and RV geometries were generated with the electroanatomical mapping system Carto3. V1 lead mainly collects RV electrical activity. Conversely, V4 mainly collects LV electrical activity. Right, sample simultaneous 5-second ECG tracings from V1 and V4 during ventricular fibrillation and their median frequency (F_{median}) values on the power spectral density (PSD). F_{median} is defined as the frequency value that divides the PSD into two subranges with equal total power. The frequency range is selected according to the physiological fibrillatory values observed in the pig (3.75 [fmin] – 20 Hz). Components with frequencies < 3.75 Hz in porcine VF signals usually reflect low-frequency baseline oscillations. The F_{median} value is represented with a vertical blue line. The frequency subranges below and above the F_{median} are represented in light orange and light green, respectively. MV: mitral valve. RVOT: RV outflow tract.

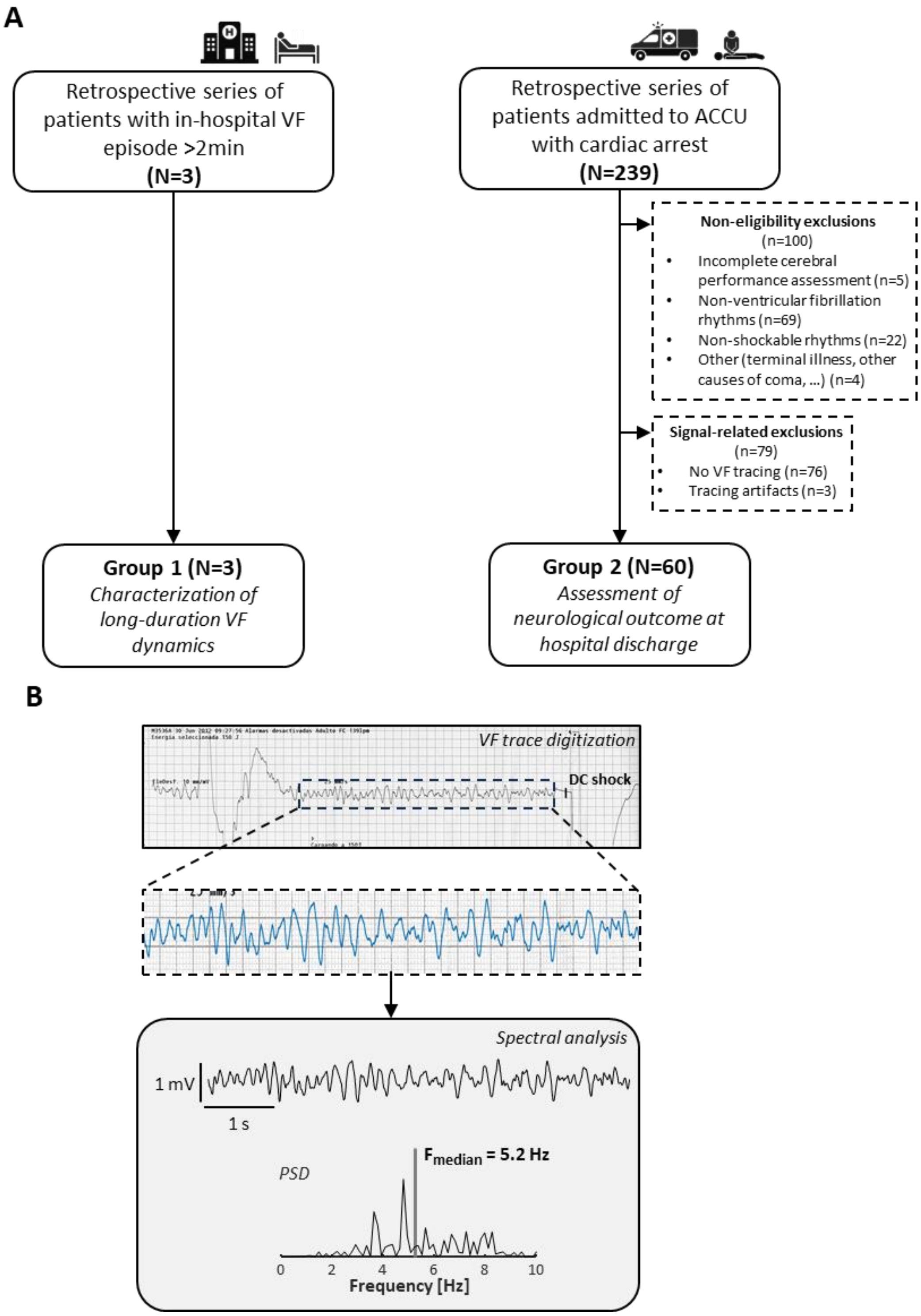

Figure S8. Flowchart of the retrospective series in patients. A, Left, patients with in-hospital ventricular fibrillation (VF) episodes, continuously monitored with single ECG leads for more than 2 minutes, were studied to characterize long-duration VF dynamics (Group 1, N=3). Right, patients admitted to hospital in comatose status (Glasgow Coma Scale ≤8) after a VF-related cardiac arrest event, and eventual return of spontaneous circulation before admission, were studied to determine the clinical value of VF activation rates to predict neurological performance at hospital discharge (Group 2, N=60). **B,** Digitization and spectral analysis process of a sample VF tracing prior to the DC shock. The VF activation rate is estimated with the median frequency (F_{median}), which divides the power spectral density (PSD) into two subranges with equal total power. The frequency range for F_{median} calculation was selected according to the physiological fibrillatory values observed in humans (2 – 10 Hz). Components with frequencies <2 Hz in human VF signals usually reflect low-frequency baseline oscillations. ACCU: Acute cardiac care unit.

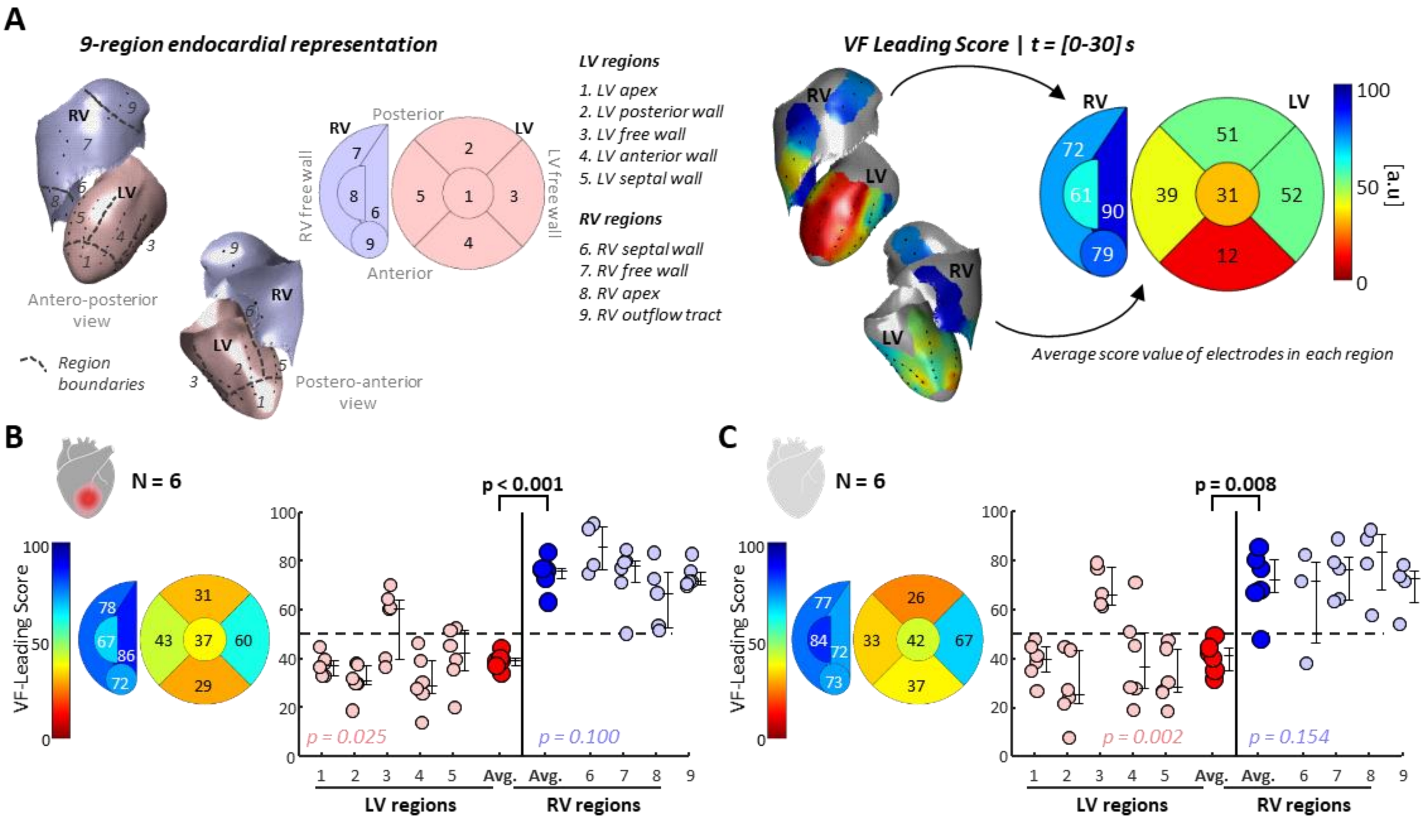

Figure. S9. Early stages *in vivo* ventricular fibrillation is characterized by homogeneously higher activation rates in the right ventricle. A, Left, sample visualization of intra-cavitary catheter electrodes on the endocardial surface of the left and right ventricles (LV and RV, respectively). Electrodes were assigned to one of 9 endocardial regions and visualized in a 2D bird's-eye schematic. Right, sample representation on 3D and 2D maps of the ventricular fibrillation (VF)-leading score. The VF-leading score summarizes the regional hierarchies in activation rates during short-duration VF (SDVF, see Figure S6). **B, C** Quantification and comparison of the average VF-leading score of each endocardial region in pigs with established myocardial infarction (MI) (**B**) and healthy controls (**C**) (N=6 for each group). Left (**B**, **C**), 2D maps of the median VF-leading score of each region for pigs with established MI (**B**) and controls (**C**). Right (**B, C**) Each circle represents the average VF-leading score of a pig within a specific region (N<6 if a region hosted no bipoles). In **B, C**, data are shown as median and interquartile range. Intra-ventricular comparisons were performed using one-way ANOVA followed by Tukey post-hoc correction. P-values for overall intra-ventricular comparisons are shown in light red/blue for the LV/RV. Average VF-leading scores of all LV and RV regions are also shown (dark red and blue circles), and were compared using paired t-test (p-value in black).

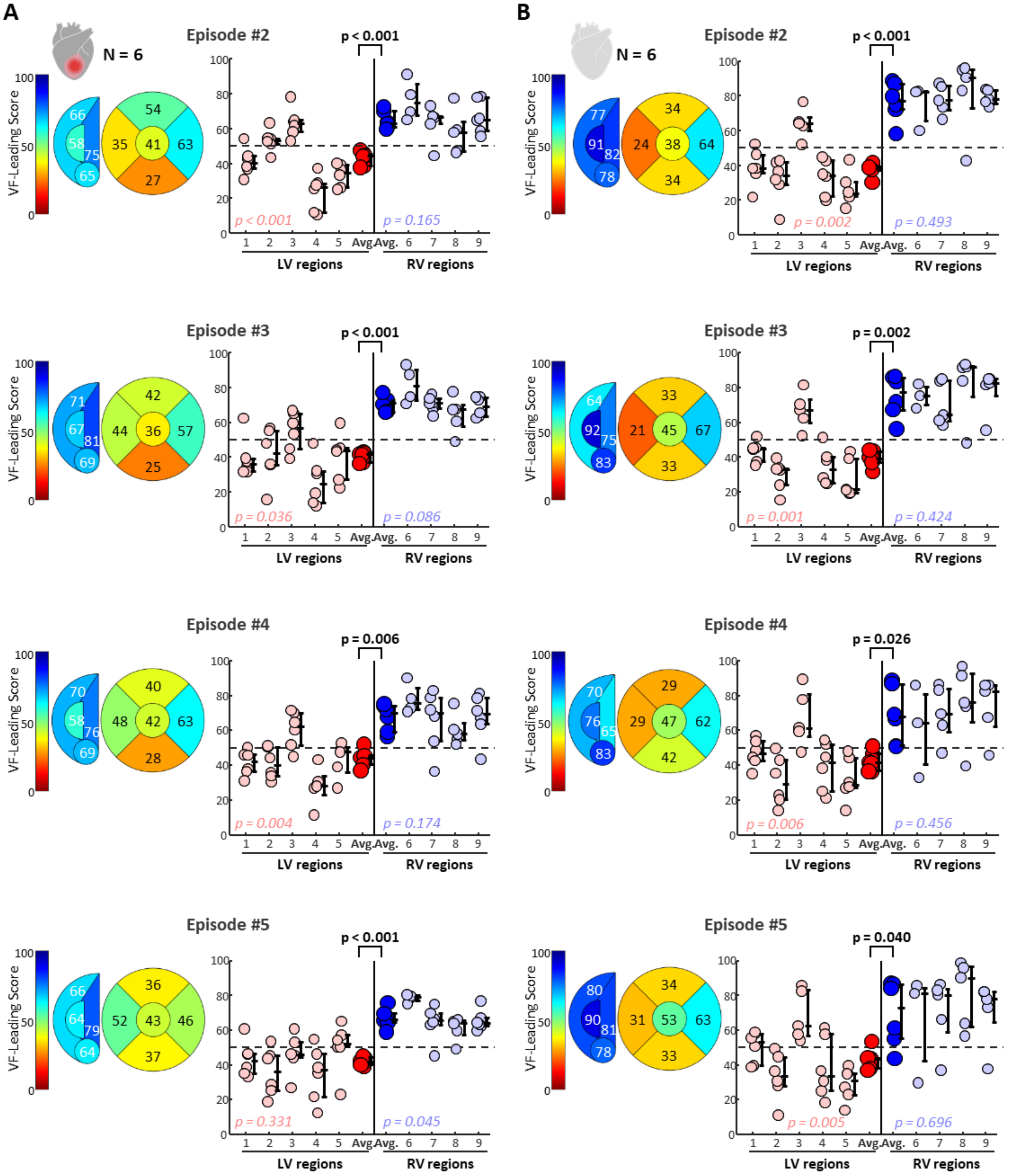

Figure S10. Ventricular fibrillation leading score analysis of *in vivo* endocardial electrograms from successive episodes of short duration ventricular fibrillation A, B, Ventricular fibrillation (VF) leading score of successive VF episodes in healthy controls (**A**) and animals with established myocardial infarction (**B**) (N=6 for each group). Episode 1 in infarcted animals and healthy controls are shown in Figure S9B and S9C, respectively. Endocardial regions are the same as in Figure S6 and S9. In each panel: Left, summarized 2D representation of the median VF-leading score of the data distribution shown on its right. Right, quantification and comparison of the average VF-leading score of each endocardial region and pig. Each circle represents the average VF-leading score of a pig for each region. In some regions the N was <6 since such regions did not have any bipole located in there. Data are shown as median and interquartile range. Intra-ventricular comparisons were performed using one-way ANOVA followed by Tukey post-hoc correction. P-values for overall intra-ventricular comparisons are shown in light red/blue for the LV/RV. Average VF-leading scores of all LV and RV regions are also shown (dark red and blue circles), and were compared using paired t-test (*p*-value in black).

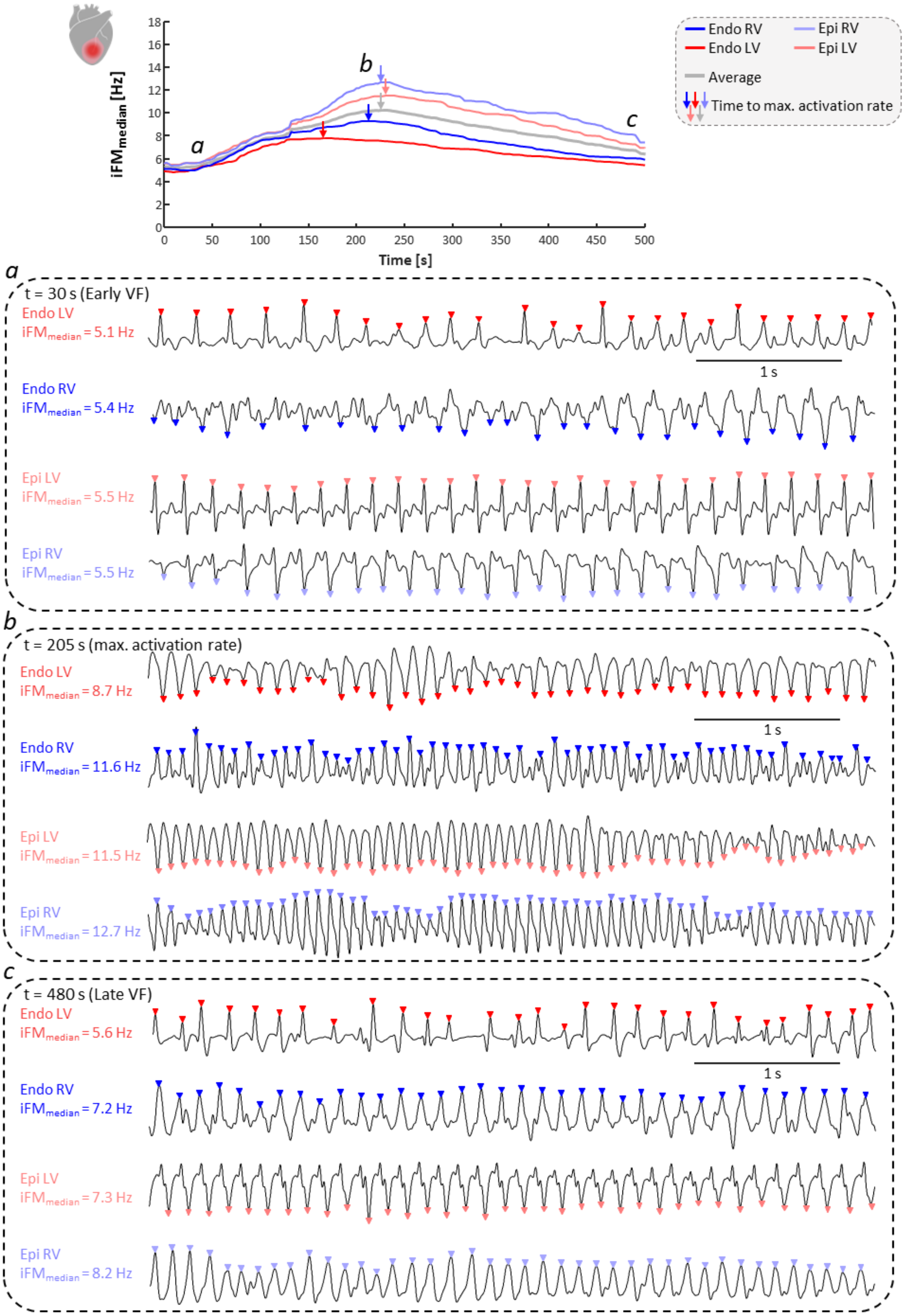

Figure S11. Time-course of endocardial and epicardial activation rates during *in vivo* long-duration ventricular fibrillation in a pig with established myocardial infarction. Top row, sample time-course of the average median instantaneous frequency modulation (iFM_{median}) values of the bipoles located in the epicardium and endocardium of the left and right ventricles (LV and RV, respectively) during an *in vivo* long-duration ventricular fibrillation (VF) episode of a pig with established myocardial infarction. The iFM_{median} average of the mapped surfaces is shown in grey. A color-coded vertical arrow indicates the time-to-maximal iFM_{median} in each mapped surface and in the average tracing. Below, sample bipolar electrograms from each myocardial wall at significant timepoints, coded with a letter in the top graph (***a-c***). Temporal annotations used to calculate iFM are shown in each electrogram with coloured triangles.

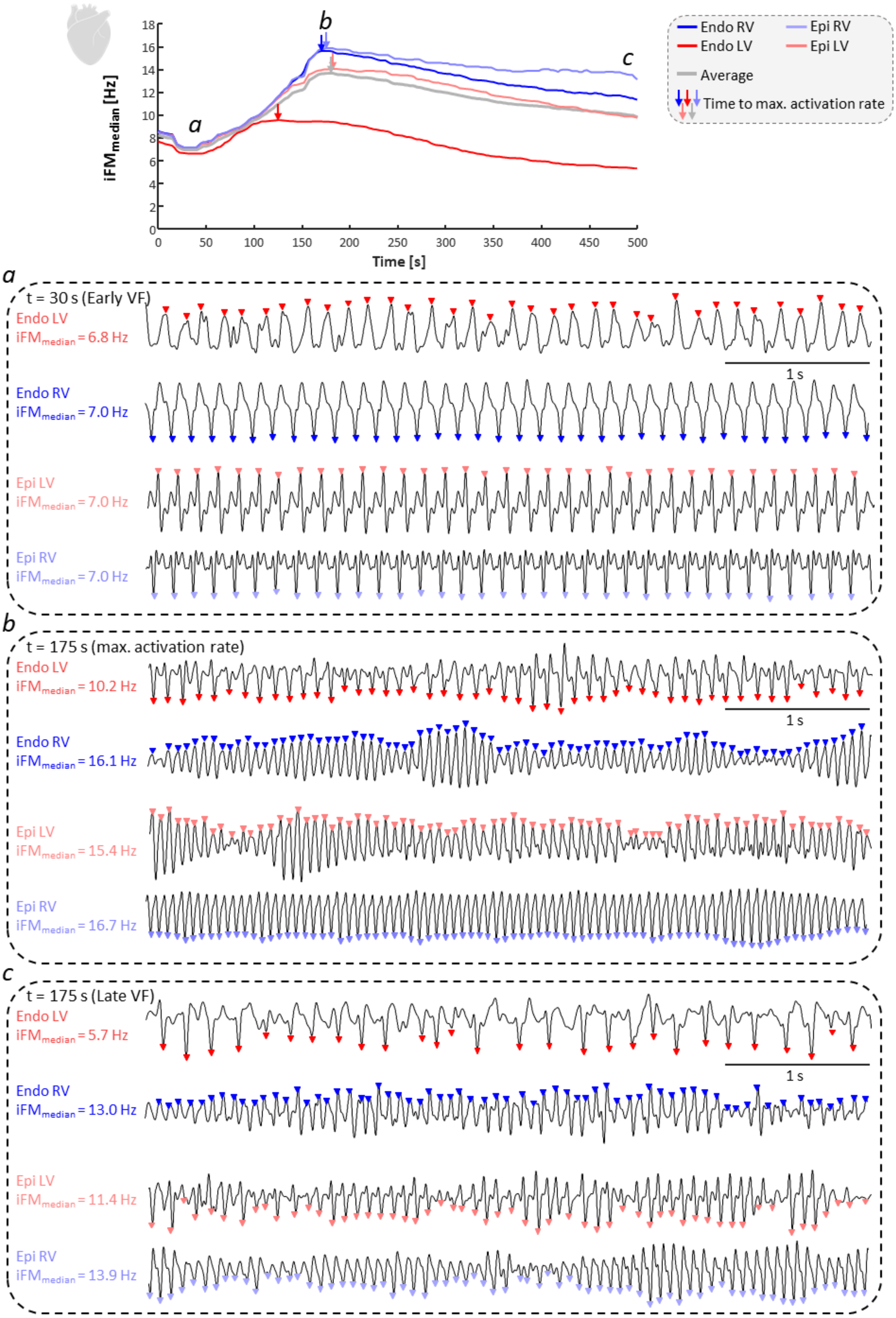

Figure S12. Time-course of endocardial and epicardial activation rates during *in vivo* long-duration ventricular fibrillation in a healthy control. Top row, sample time-course of the average median instantaneous frequency modulation (iFM_{median}) values of the bipoles located in the epicardium and endocardium of the left and right ventricles (LV and RV, respectively) during an *in vivo* long-duration ventricular fibrillation (VF) episode. The iFM_{median} average of the mapped surfaces is shown in grey. A color-coded vertical arrow indicates the time-to-maximal iFM_{median} in each mapped surface and in the average tracing. Below, sample bipolar electrograms from each myocardial wall at significant timepoints, coded with a letter in the top graph (***a-c***). Temporal annotations used to calculate iFM are shown in each electrogram with coloured triangles.

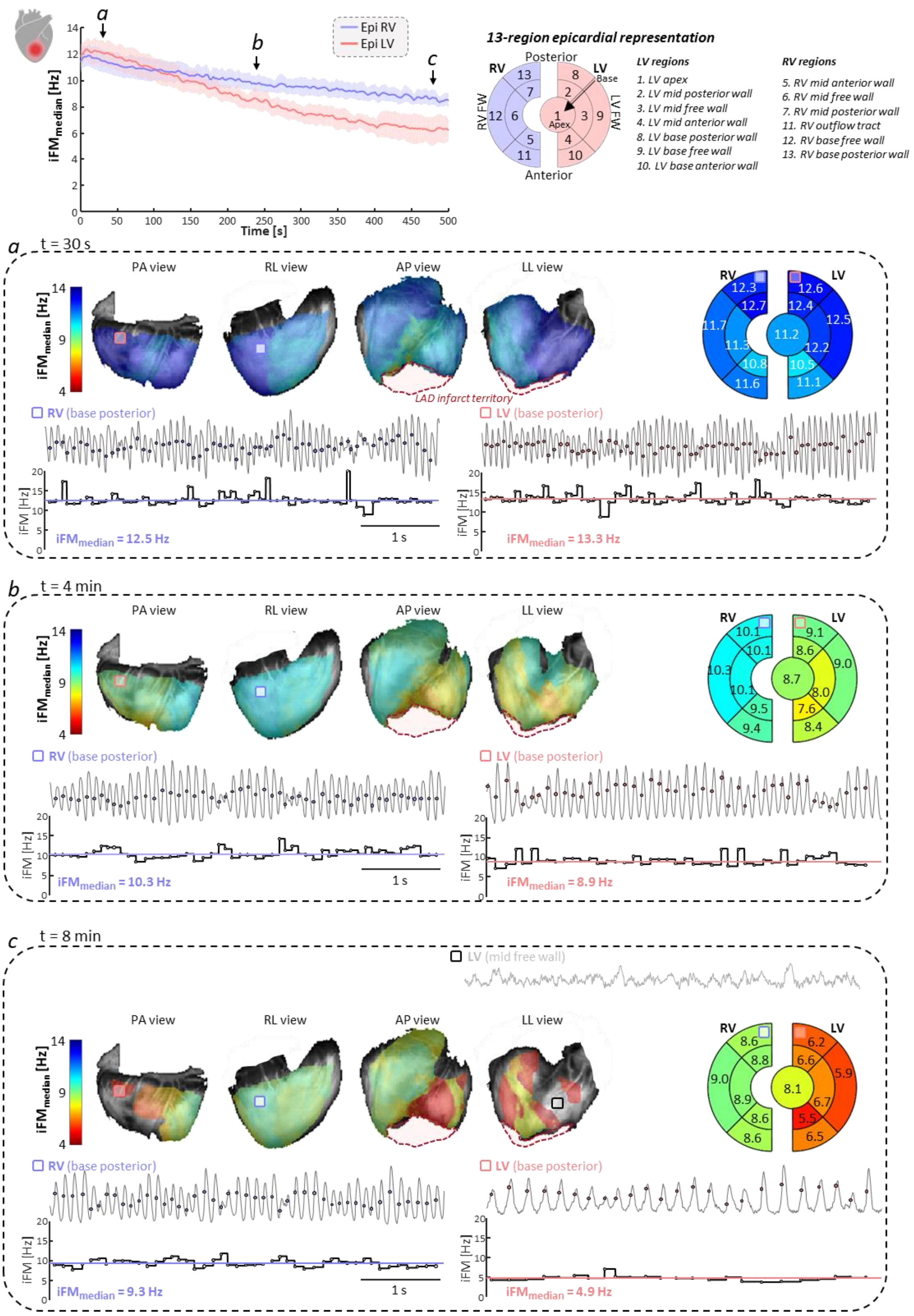

Figure S13. Time-course of activation rates during an *ex vivo* long-duration ventricular fibrillation episode in an animal with established myocardial infarction. Top row, time-course of the average median instantaneous frequency modulation (iFM_{median}) values of the left and right ventricles (LV and RV, respectively) during a long-duration ventricular fibrillation (VF) episode. Shaded areas surrounding the mean represent ±1 standard deviation. Below, panoramic iFM_{median} maps at significant timepoints, coded with a letter in the top graph (***a***-***c***). The color-coded map is super-imposed over the grey-scaled raw image of the heart. iFM analysis was not performed in pixels with poor signal-to-noise. The expected infarcted territory is drawn in the top map (note no fluorescence is emitted by infarcted areas since the voltage sensitive dye does not reach such territories and they display little or no electrical activity at all). The 13-region epicardial schematic representation is shown to the right. Sample optical signals from LV and RV pixels highlighted in the maps as squares are shown below the corresponding map. The temporal annotations used to calculate iFM are marked in each signal at times of maximal positive slope with coloured circles. The resulting iFM signal is shown below each optical signal. A sample optical signal is also shown from a large LV region with electrical depression during late VF stages.

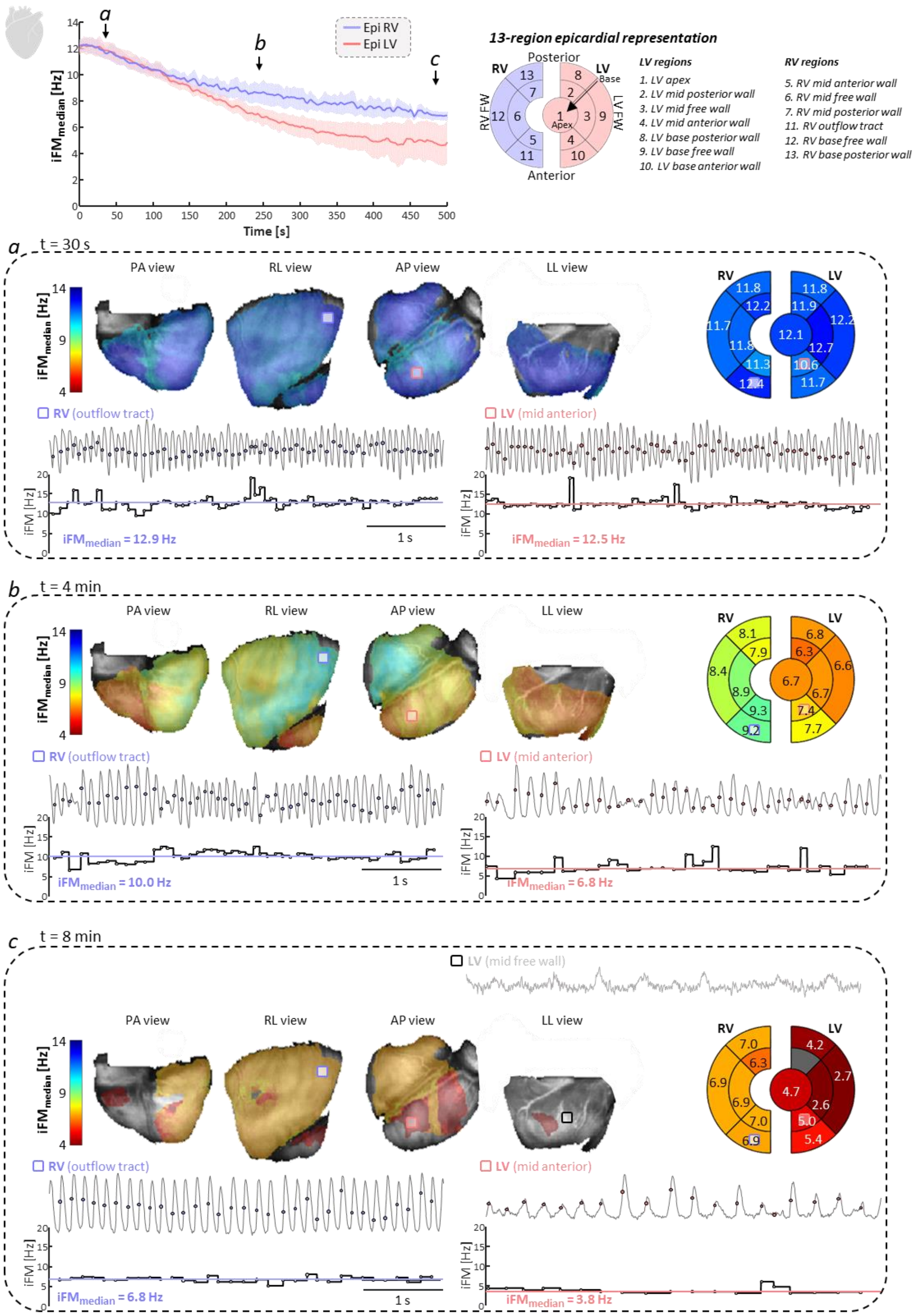

Figure S14. Time-course of activation rates during an *ex vivo* long-duration ventricular fibrillation episode in a healthy control. Top row, time-course of the average median instantaneous frequency modulation (iFM_{median}) values of the left and right ventricles (LV and RV, respectively) during a long-duration ventricular fibrillation (VF) episode. Shaded areas surrounding the mean represent ±1 standard deviation. Below, panoramic iFM_{median} maps at significant timepoints, coded with a letter in the top graph (***a-c***). The color-coded map is super-imposed over the grey-scaled raw image of the heart. iFM analysis was not performed in pixels with poor signal-to-noise ratio. The 13-region epicardial schematic representation is shown to the right. Sample optical signals from LV and RV pixels highlighted in the maps as squares are shown below the corresponding map. The temporal annotations used to calculate iFM are marked in each signal at times of maximal positive slope with coloured circles. The resulting iFM signal is shown below each optical signal. A sample optical signal is also shown from a large LV region with electrical depression during late VF stages.

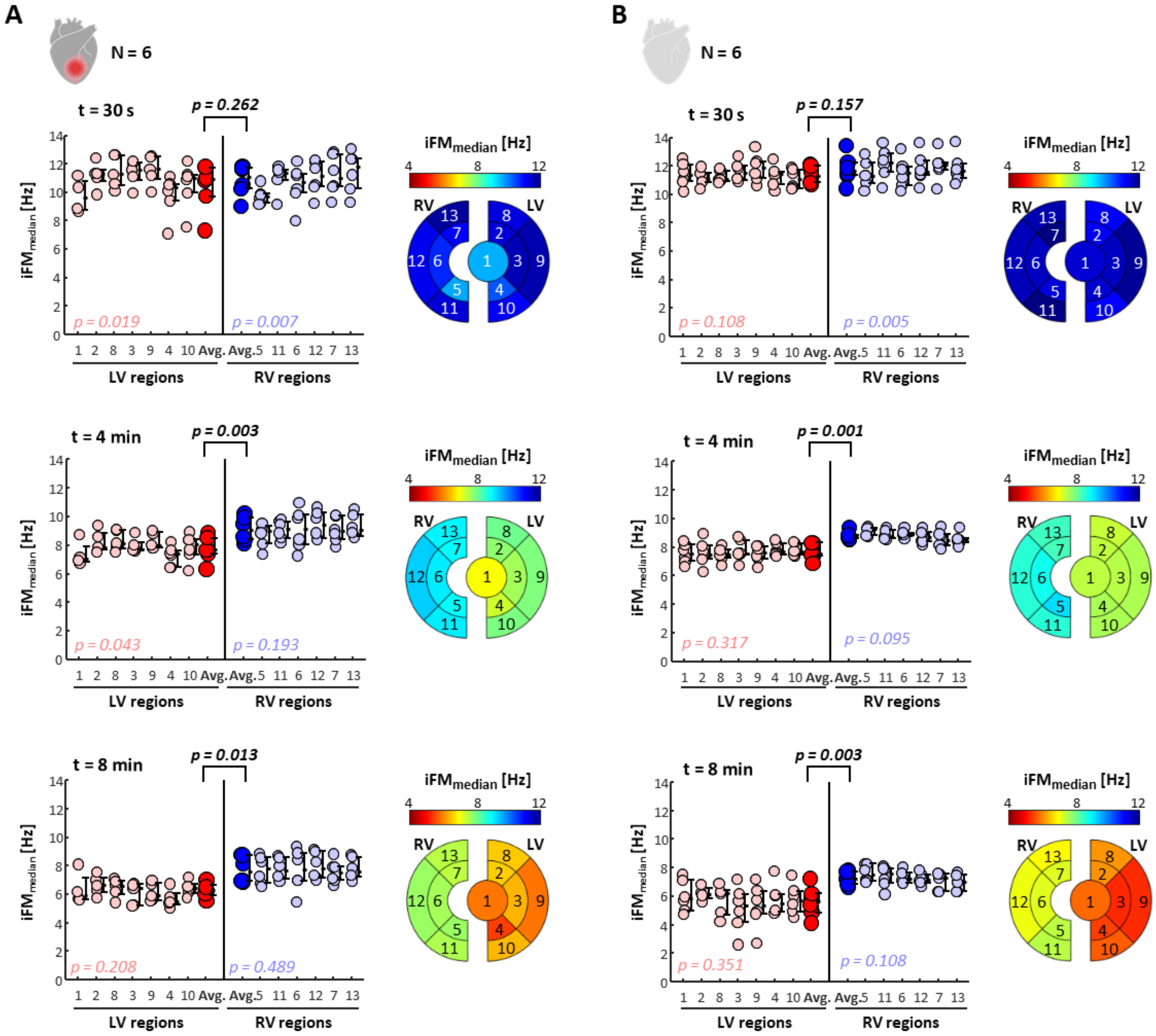

Figure S15. Median instantaneous frequency modulation in epicardial optical signals during *ex vivo* long-duration ventricular fibrillation episodes. A, B, Quantification and comparison of the average median instantaneous frequency modulation (iFM_{median}) values of the 13 epicardial regions, in pigs with established myocardial infarction (**A**) and in healthy controls (**B**), at different timepoints of a long-duration ventricular fibrillation episode (30 seconds: top row; 4 minutes: middle row; 8 minutes: bottom row) (N=6 for each group). Each circle represents the average iFM_{median} for each region and pig. Data are shown as median and interquartile range. Intra-ventricular comparisons were performed using one-way ANOVA followed by Tukey post-hoc correction. P-values for overall intra-ventricular comparisons are shown in light red/blue for the LV/RV. Average iFM_{median} of all LV and RV regions are also shown (dark red and blue circles), and were compared using paired t-test (*p*-value in black). Summarized 2D representation of the median values of the corresponding data distribution is shown to the right of each sub-panel. Regions are labelled 1-13 and are the same as in Figure 3, S13 and S14.

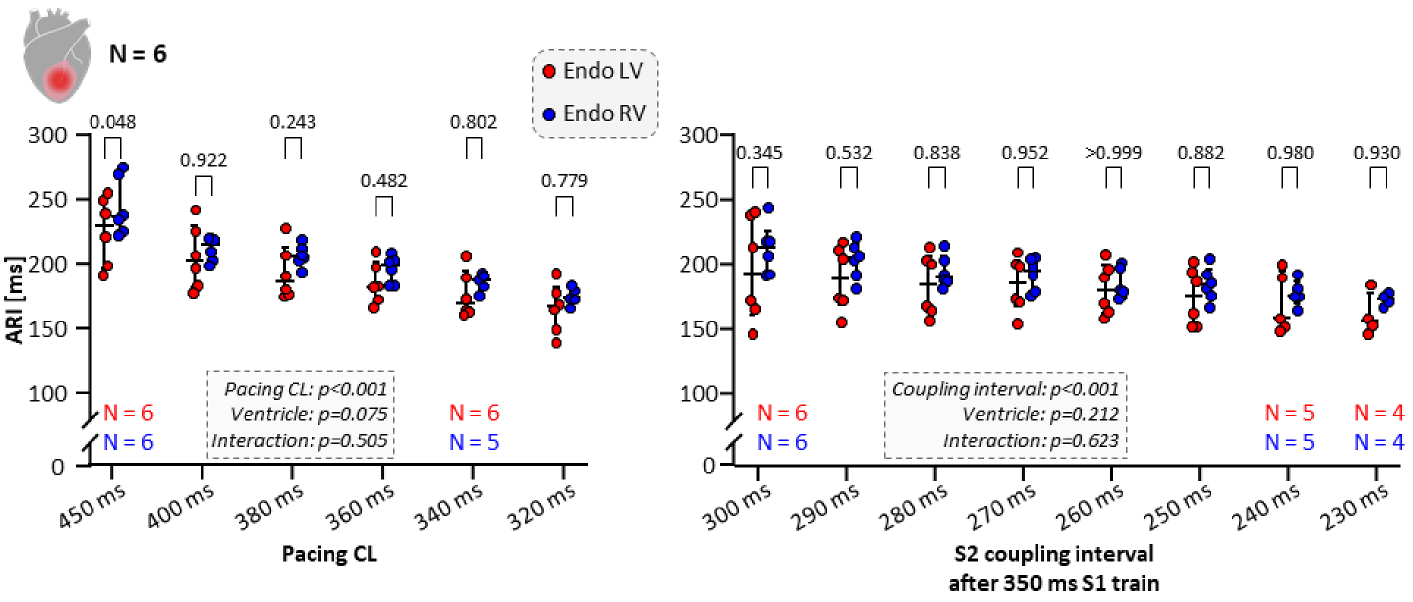

Figure S16. Analysis of *in vivo* activation-recovery intervals. Quantification and comparison of the activation-recovery interval (ARI) measured in unipolar recordings from the endocardium of the left and right ventricles (LV and RV, respectively) at different pacing cycle lengths (CL) and with different coupling intervals of an S2 extrastimulus. The protocol was performed in pigs with established myocardial infarction and started with S1 pacing at different basic drive CL from 450 ms to 320 ms. Then, an S1-S2 protocol was applied to reach shorter coupling intervals (N=6) closer to a ventricular fibrillation CL. When the number of pigs with available data decreased in the LV or the RV due to refractoriness or the risk of inducing ventricular arrhythmia, the updated ‘N’ is specified below the specific comparison (in red for the LV and blue for the RV). Two-way ANOVA followed by Sídák post-hoc correction was used.

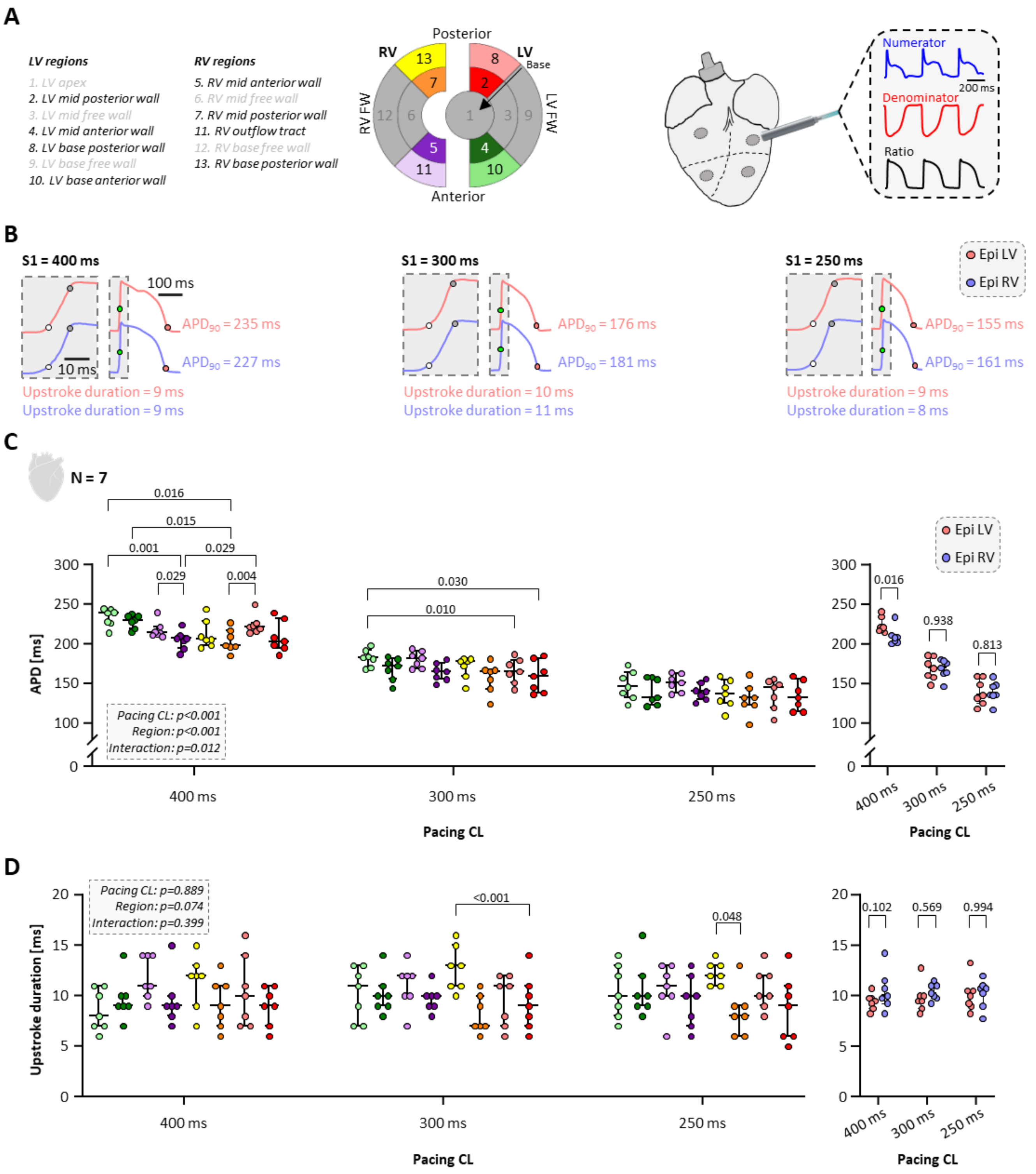

Figure S17. Analysis of *ex vivo* action potentials. A, Top left, list and 2D schematic of the epicardial regions, in which action potential duration (APD) measurements were taken during the *ex vivo* pacing protocol in healthy controls. Regions with available ratiometric signals of transmembrane voltage changes are coloured and highlighted in black. Top right, sample blue and red fluorescence signals recorded with the optical fibre, corresponding to the numerator and denominator signals, respectively. The ratio of these signals yields an optical action potential with minimal motion artefact (black). **B,** Sample optical action potentials from epicardial regions of the left and right ventricles (LV and RV, respectively) during pacing at three different cycle length (CL). Annotation of the activation at the time of maximum slope is marked with a green circle, and annotation at the time of 90% repolarization is marked with a red circle. The difference between these two timepoints yields the action potential duration (APD). To the left of each action potential, a zoom-in to the upstroke is shown. The times of 10% and 90% depolarization are shown with white and grey circles, respectively. The difference between these two timepoints yields the upstroke duration. **C and D,** Left, quantification and distribution of the regional APDs (**C**) and upstroke durations (**D**) measured during the *ex vivo* pacing protocol, color-coded according to the 2D schematic in **A** (N=7). Regional comparisons with $p<0.05$ are specified. Two-way ANOVA followed by Tukey post-hoc correction was used. Right, quantification and comparisons of the average APD (**C**) and upstroke durations (**D**) in different regions of the RV and LV. Multiple Wilcoxon tests with Holm-Sídák correction was performed.

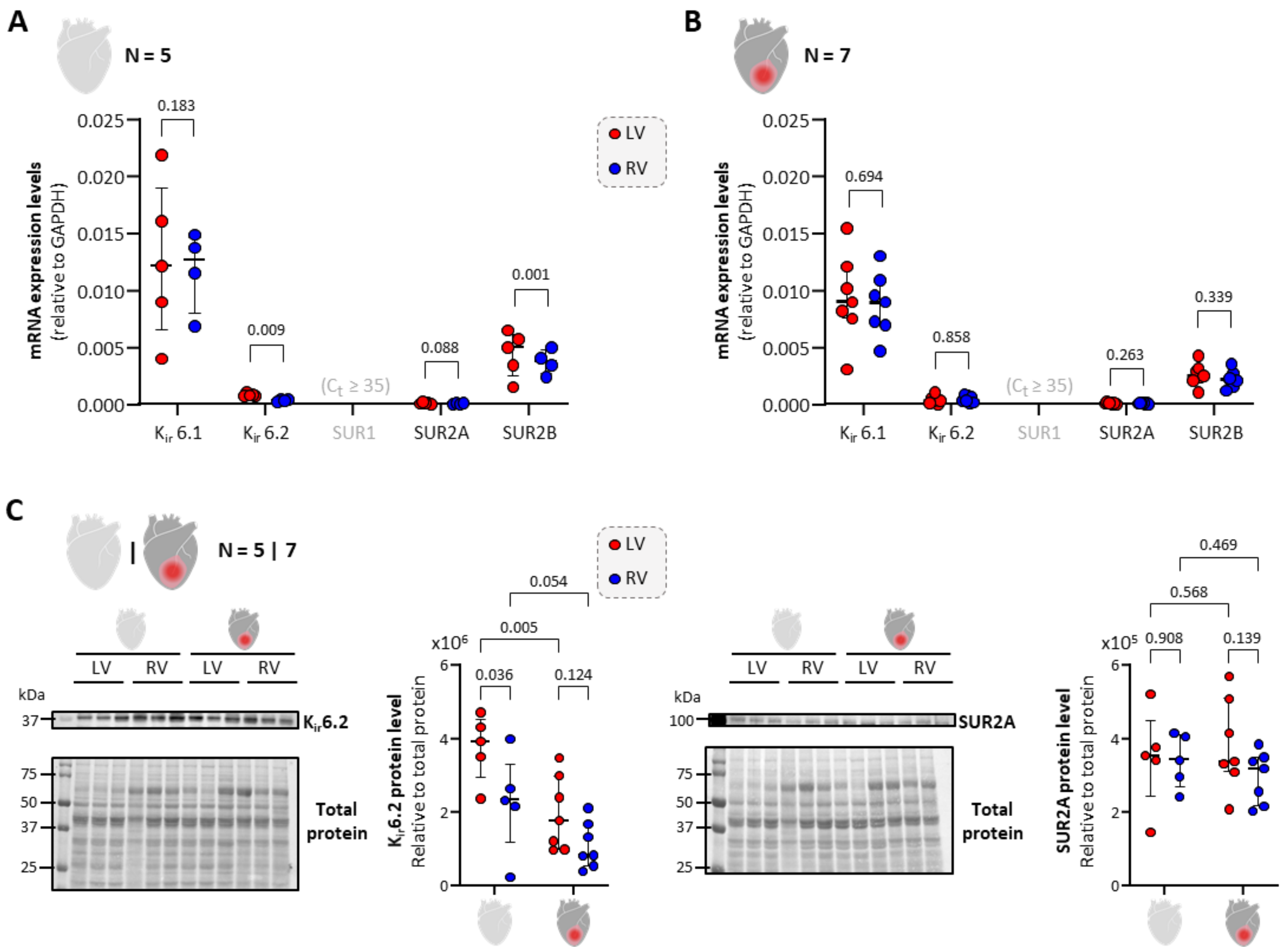

Figure S18. Gene and protein expressions of ATP-sensitive potassium channel subunits in infarcted animals and healthy controls. A, B, mRNA expression levels assessed by RT-qPCR of the 5 subunits of the ATP-sensitive potassium (K_{ATP}) channel, in left and right ventricular (LV and RV, respectively) samples collected in control animals (**A**) and in animals with established myocardial infarction (**B**) (N=5 and 7, respectively). Expression levels are relative to GAPDH levels. Values expressing a $C_t \geq 35$ were discarded (only observed in SUR1). One RV sample from a control pig did not express $C_t < 35$ in any gene. Two-way ANOVA followed by Tukey post-hoc correction was used. **c** Western blot analysis and quantification of K_{ATP} channel subunits K_{ir}6.2 and SUR2A in controls and infarcted animals. Protein expression of K_{ir}6.1 and SUR2B channel subunits are shown in Figure 4G. SUR1 channel subunit is not shown since no gene expression was detected by RT-qPCR. Protein levels are expressed relative to the total protein. Two-way ANOVA was used.

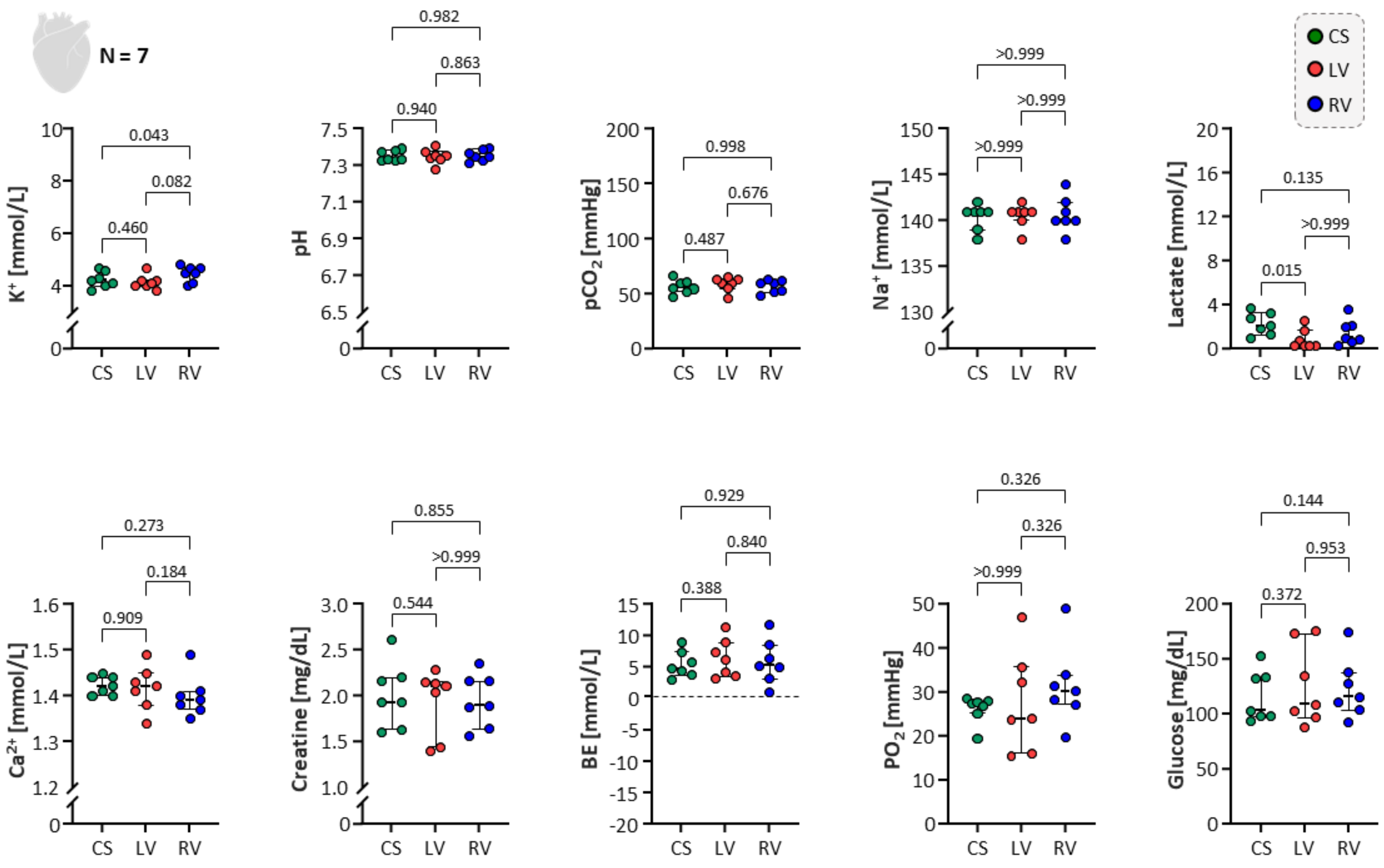

Figure S19. *In vivo* baseline analyses of electrolytes and blood gases in healthy controls. Quantification and comparisons of several parameters measured in blood samples from coronary veins specifically draining from the left ventricle or the right ventricle (LV or RV) (N=7). The coronary veins were cannulated using a small size intravenous line (24 G, 0.7x19 mm). Blood samples (~100 µL) were drawn and analysed using the epoc® Blood Analysis System (Siemens Healthineers, Germany). Blood samples was also taken from the coronary sinus (CS) to measure the same parameters as reference. Baseline measurements refer to data before ventricular fibrillation induction. One-way ANOVA with Tukey post-hoc correction was used for normally distributed parameters (K^+, pH, pCO_2, Ca^{2+}, base excess [BE] and glucose) and Dunn post-hoc correction for non-normally distributed parameters (remaining).

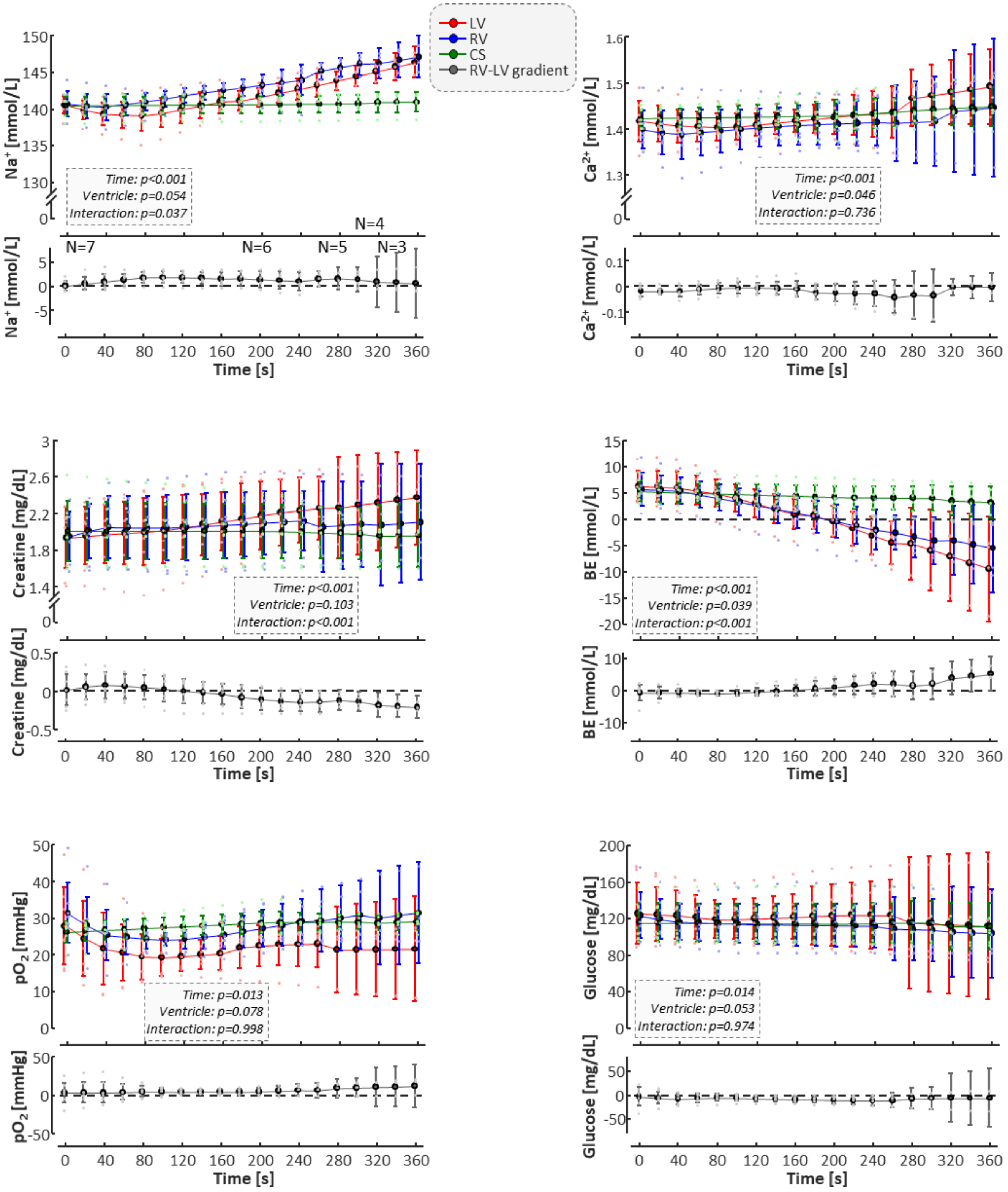

Figure S20. Time-course of metabolic parameters measured in blood samples taken from right and left ventricular coronary veins during ventricular fibrillation. Time-course and quantification of Na^{+}, Ca^{2+}, creatine, base excess (BE), pO_2 and glucose values measured in blood samples taken from left and right ventricular coronary veins (LV and RV, respectively) during ventricular fibrillation (N=7). Data source is the same as in Figure 5. Parameters were assumed to follow a linear progression between samples and until the last sample. After data normality testing, data are shown with mean and 95% confidence interval. Measurements were also taken from blood samples of the coronary sinus (CS) as reference. The paired RV-to-LV gradient is shown in grey. 'N' values shown in top-left panel are the same in all panels (updated whenever the final sample of a pig was taken). Two-way ANOVA followed by Sídák post-hoc correction was used.

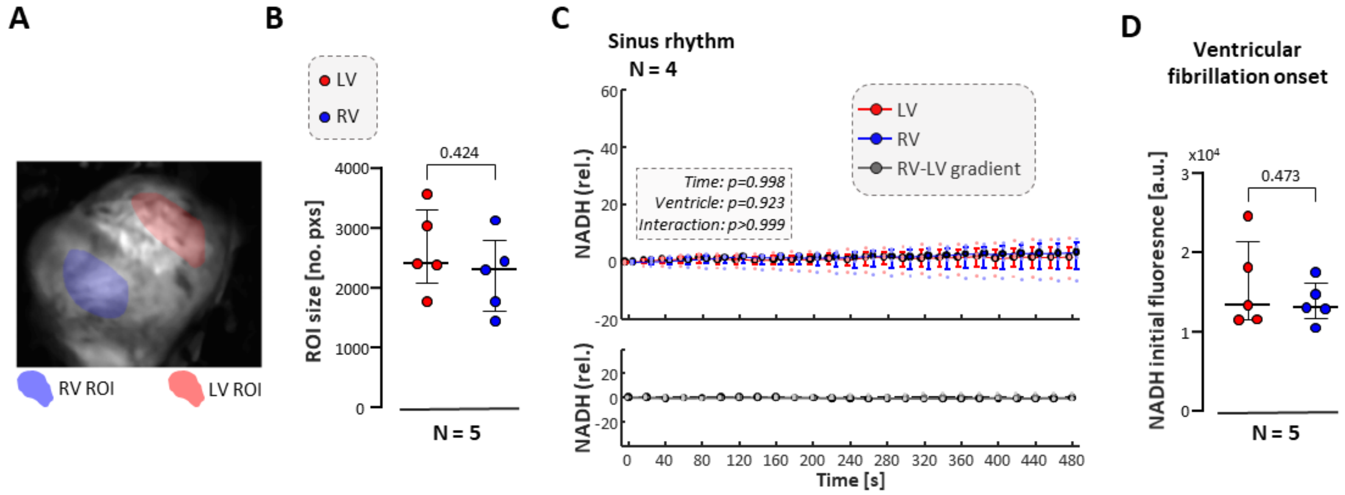

Figure S21. ***In vivo*** **cardiac NADH autofluorescence in healthy animals. A,** Sample frame from a filtered movie of cardiac NADH autofluorescence. The pig heart was exposed via median sternotomy and excited with an ultra-violet lamp, aiming at illuminating homogeneously the antero-lateral walls of the left ventricle (LV) and the right ventricle (RV). Regions of interest (ROI) with comparable size and illumination were delimited for the LV and RV (red and blue areas, respectively). **B,** Quantification and comparison of the size of the selected LV and RV ROIs. **C,** Time-course of average NADH autofluorescence of the LV-ROI and RV-ROI relative to fluorescence values of the initial frame in an 8-minute movie during sinus rhythm. Paired RV-to-LV gradient is shown below. Data are expressed as median and interquartile range. Note that during normoxic sinus rhythm, no NADH accumulation is observed, as aerobic respiration is preserved. **D,** Quantification and comparison of the average NADH autofluorescence of the selected LV and RV ROIs in the initial frame of the recorded ventricular fibrillation episode. In **B and D**, paired t-test was performed. In **C**, two-way ANOVA followed by Sídák post-hoc correction was used.

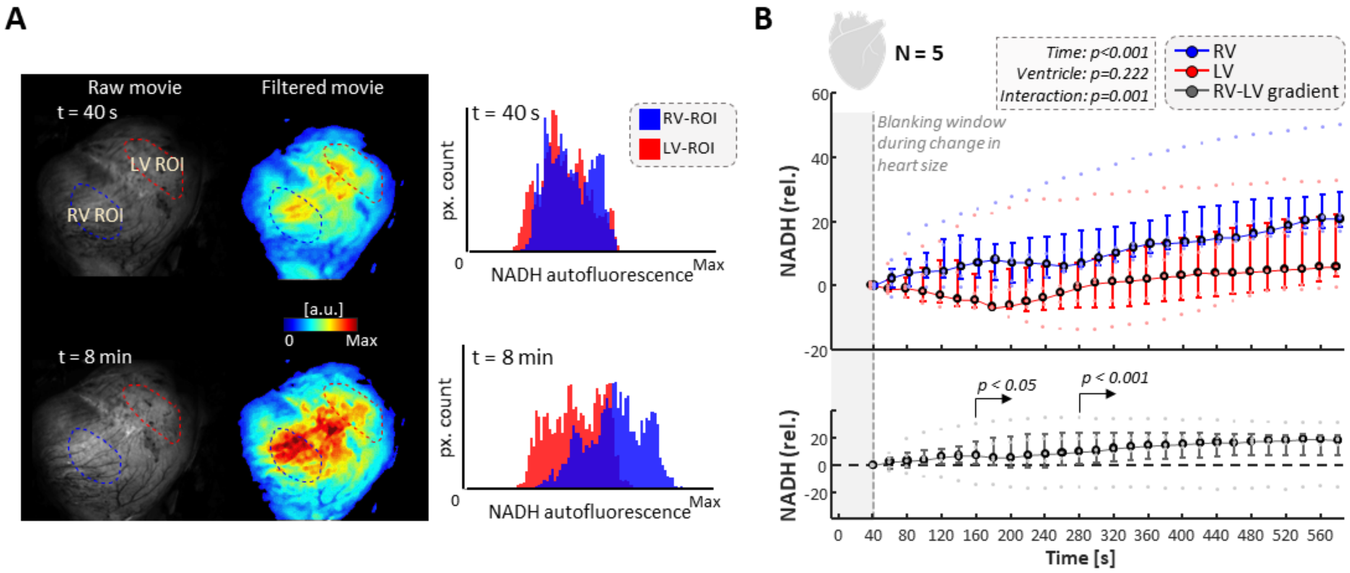

Figure S22. ***In vivo*** **cardiac NADH autofluorescence during long-duration ventricular fibrillation episodes in healthy animals.** In 5 healthy animals, the heart was exposed, and an ultra-violet light lamp was used to homogeneously illuminate the visible epicardial surface and measure NADH autofluorescence during ventricular fibrillation (VF). NADH autofluorescence during VF aimed to provide an indirect marker of aerobic-respiration disruption. A 40-second blanking window was applied following VF onset to minimize signal artefacts associated with sudden changes in heart size and position. These mechanical changes are expected due to passive ventricular filling and dilation resulting from the cessation of effective myocardial contraction upon VF. **A,** Sample case showing the t=40 second (top) and the t=8-minute frame (bottom) NADH autofluorescence analysis during a long-duration VF episode. Left, raw (left) and filtered (right) frames showing NADH autofluorescence. Filtered movies are colour-scaled to show differences in NADH accumulations. Scale limits are the same for both frames. The selected left and right ventricular (LV and RV, respectively) regions of interest (ROI) are drawn with coloured dashed contours. Right, NADH autofluorescence histogram of the LV and RV ROIs of the corresponding frames on the left. **B,** Time-course of NADH autofluorescence within the LV-ROI and RV-ROI over 10-minute VF episodes in all animals. NADH autofluorescence is shown relative to fluorescence values of the initial frame. Paired RV-to-LV gradient is shown below. Data are expressed as median and interquartile range. The greater increase in NADH autofluorescence in the RV may suggest a superior metabolic adaptation to global ischaemia conditions and a more efficient shift towards anaerobic energy production. Two-way ANOVA followed by Sídák post-hoc correction was used.

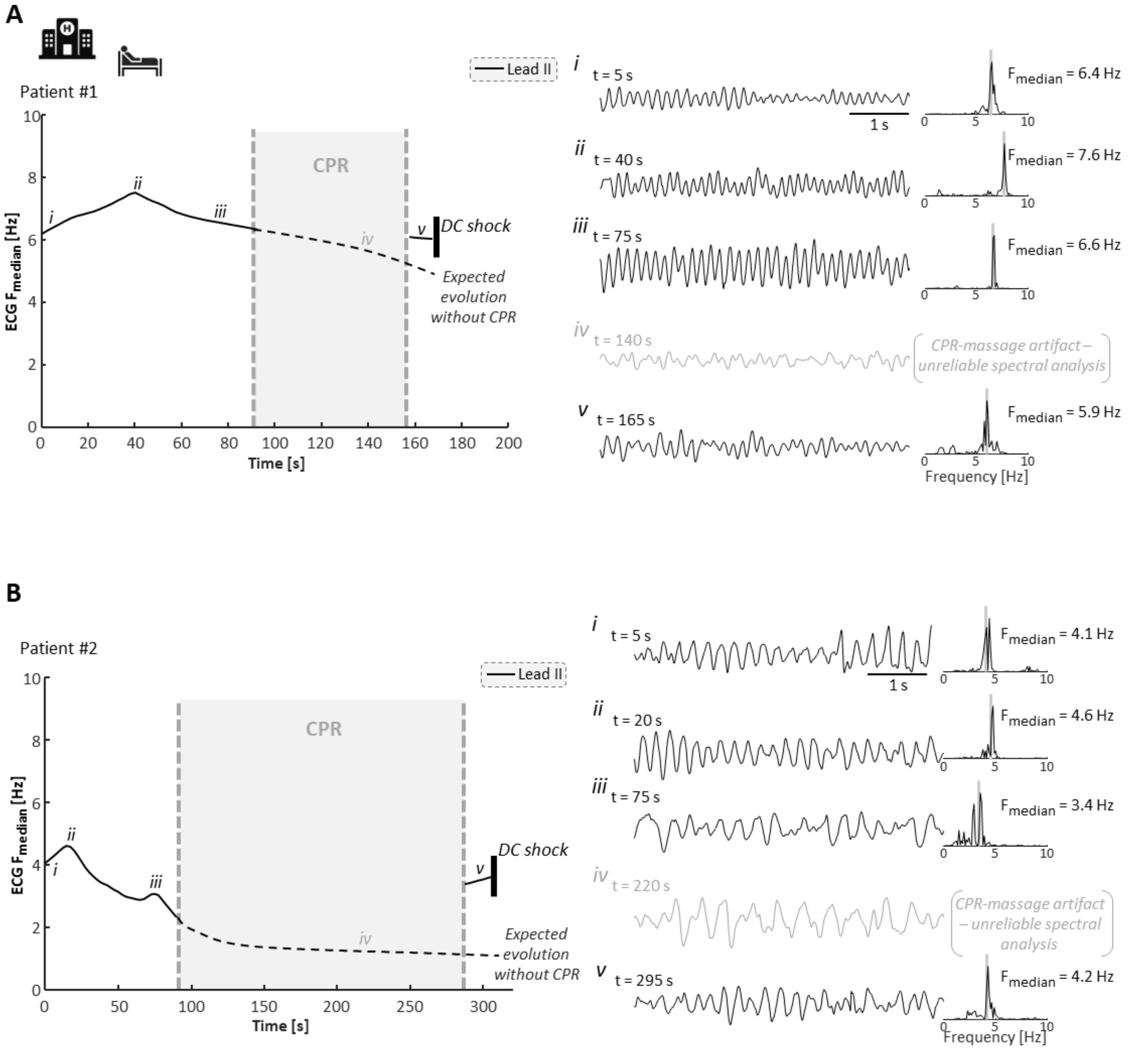

Figure S23. Time-course of surface ECG-derived activation rates during long-duration in-hospital ventricular fibrillation. A, B, Left, time-course of the median frequency (F_{median}) values in lead II tracing during in-hospital ventricular fibrillation (VF) episodes of 2 patients (**A, B**). Spectral analysis during the cardiopulmonary resuscitation (CPR) massage was not possible due to motion artefact, and therefore the F_{median} evolution is not shown during the manoeuvre (shaded in grey). Spectral analysis was possible again during the final seconds of VF prior to the DC shock, when CPR is interrupted. The expected evolution of F_{median} values without CPR is sketched (dashed black line) to highlight the influence of CPR manoeuvres on the activation rate. Right, sample ECG tracings and their frequency-domain analyses at specific timepoints, coded i-v in the corresponding graph. F_{median} values are shown with a grey vertical line in the spectrum.

SUPPLEMENTARY MATERIAL AND METHODS

Experimental animal procedures and protocols

The study was designed as a pilot study and the sample size was estimated to ensure the use of the minimum number of animals to address the hypothesis and underlying mechanisms. No a priori exclusion criteria were used, and no animals were excluded after experimental completion. Animals were allocated to each experimental group in a non-randomized sequential manner, and no possible confounding factors were identified. Animal experiments complied with ARRIVE guidelines, were performed at Centro Nacional de Investigaciones Cardiovasculares (Madrid, Spain), were approved by the competent ethical authority (Comunidad de Madrid, Ref#PROEX097/17 & Ref#PROEX078.8/21) and conformed to the regulations outlined in the EU Directive 2010/63EU and Recommendation 2007/526/EC regarding the protection of animals used for experimental and other scientific purposes, enforced in Spanish law under RD53/2013 and ECC/566/2015.

General anaesthesia

General anaesthesia was induced by intramuscular ketamine injection (15 mg/kg), xylazine (2 mg/kg) and midazolam (0.5 mg/kg). Animals undergoing invasive *in vivo* procedures were further intubated and mechanically ventilated with oxygen (fraction of inspired O_2 of 21%) and anaesthesia was maintained with continuous intravenous infusion of ketamine (2 mg/kg/h), xylazine (0.2 mg/kg/h) and midazolam (0.2 mg/kg/h). During the *in vivo* procedure, electrocardiogram, oxygen and carbon dioxide levels were monitored. The femoral artery was cannulated and used to continuously monitor invasive blood pressure. Animals following a specific protocol requiring open-chest surgery to expose and/or excise the heart were anesthetized with a combination of Fentanyl (0.010 mg/kg) and Sevoflurane (3.5%). Then, the heart was exposed via median sternotomy, and 300 UI//Kg of unfractionated heparin (300 UI/kg) were administered before instrumentation or euthanasia. At the time of euthanasia and heart removal, VF was induced with a 9 VDC battery. The latter aimed to prevent circulating bubbles from the left ventricle (LV) to the coronary arteries at the time of heart excision.

Porcine model of myocardial infarction

A subgroup of pigs underwent an ischaemia-reperfusion protocol to obtain a clinically relevant model of established myocardial infarction, as reported elsewhere.[1] Briefly, pigs underwent percutaneous catheterization of the left anterior descending coronary artery (LAD) to inflate an angioplasty balloon and occlude the artery for 60 minutes. The balloon was inflated either proximal or distal to the first diagonal branch to generate different infarct sizes and variable scar distributions. Balloon location and inflation were monitored angiographically to confirm complete occlusion. A continuous infusion of intravenous amiodarone (150 mg/h) was administered to decrease the incidence of ventricular malignant arrhythmias. In case of ventricular fibrillation (VF) during the ischaemia-reperfusion protocol, a non-synchronized DC shock was delivered with a biphasic defibrillator. After 60 minutes of occlusion, the balloon was deflated, and a coronary angiogram was recorded to confirm patency of the coronary artery and reperfusion. Animals were allowed to recover for 5 days prior to the transfer to specific animal research facilities. Subsequent procedures in animals with myocardial infarction were performed a median of 14 (9, 28 – interquartile range) weeks after the ischaemia-reperfusion protocol, when the myocardial infarction substrate was well-established.[1] Table S1 summarizes the number of animals and characteristics within each experimental group.

Programmed ventricular stimulation during invasive electrophysiological studies

Ventricular stimulation was applied from a screw-in catheter positioned in the right ventricle (RV). Two programmed ventricular stimulation (PVS) protocols were performed during invasive electrophysiological studies:

- VF induction: VF was induced using an S1 basic drive cycle length (CL) pacing (10 pacing beats) at 350, 300, 280, 260 and 250 ms followed by a maximum of four coupled extrastimuli (S2, S3, S4, S5). At each basic drive pacing CL, the extrastimuli were progressively decremented in 10 ms steps until ventricular arrhythmia was induced, refractoriness or a 180 ms coupling interval. In case of monomorphic ventricular tachycardia (VT) induction, the tachycardia was allowed to self-degenerate into polymorphic VT or VF. The VT was degenerated into VF with burst pacing, if the monomorphic VT was stable and hemodynamically tolerated for >30 seconds. Burst pacing (for 10 seconds at 200 ms, 180 ms and 160 ms CL) was used to induced VF, only if the PVS protocol with coupled extrastimuli failed to induce VT or VF.

- Activation-recovery intervals to generate restitution curves: a 3.5-mm irrigated-tip mapping/ablation catheter (Navistar Thermocool, Biosense, USA) was positioned in the left ventricle (LV). Unipolar signals during basic drive cycle length (S1) at different pacing rates (450-320 ms, 10-ms decremental steps) were recorded to obtain a restitution curve of the activation-recovery interval (ARI), as a surrogate of the local action potential duration (APD). Then, during an S1 (at 350 ms CL) S2 protocol was used to measure the ARI at progressively shorter coupling intervals of the S2, aiming to reach ARI values at CLs closer to VF. The mapping/ablation catheter was then positioned in the RV, and the pacing protocol was repeated to obtain comparable RV and LV ARI measurements (Figure 4A and S16).

Ex vivo panoramic optical mapping under global ischaemia conditions

Animals were anesthetized as described in the specific section above. The heart was exposed via median sternotomy and a 9 VDC battery was used to induce VF before extraction. The latter aimed to minimize the chance of air bubbles entering the coronary arteries in a normal contracting heart. After excision, hearts were submerged in cold (4ºC) hyperkalaemic Tyrode's solution (composition in mM: NaCl 130, $NaHCO_3$ 24, NaH_2PO_4 1.2, $MgCl_2$ 1, KCl 12, Glucose 5.6, $CaCl_2$ 1.8, and albumin 0.04 g/L), cleaned, and cannulated through the aorta. Isolated hearts were then connected to a constant-flow Langendorff-perfusion system with oxygenated (O_2-CO_2, 95/5%) Tyrode's solution (in mM: NaCl 130, $NaHCO_3$ 24, NaH_2PO_4 1.2, $MgCl_2$ 1, KCl 4, Glucose 5.6, $CaCl_2$ 1.8, and albumin 0.04 g/L) at a circulating flow rate of 200-240 mL/min.[2] During perfusion, the hearts were further instrumented to achieve a closed-loop perfusion. The pulmonary artery, pulmonary veins and the inferior vena cava were ligated to close the orifices and the superior vena cava was cannulated with an open-end cannula for the outflow. A trans-septal hole was created through the fossa ovalis to equalize intra-cavitary pressures. Then, the heart was submerged inside a custom-built temperature-controlled 25-liter tank filled with saline which was maintained at 37ºC. The pH (7.4) and temperature (37°C) of the perfusate were monitored and maintained within physiological ranges throughout the experiment.

Four high-speed CMOS cameras (IDS Imaging Development Systems GmbH, Germany) (configuration 120x160 superpixels) were positioned on each of the four sides of the tank to provide a panoramic view of the ventricles.[2] Hearts were loaded with 500 µL of stock solution of a 3rd generation near-infrared voltage-sensitive dye (10 mg of di-4-ANEQ(F)PTEA [University of Connecticut School of Medicine, USA] dissolved in 3 mL of pure ethanol) diluted in 10 mL of Tyrode's solution and delivered slowly (without recirculation) over a 1-minute period through an injection port upstream the aortic cannula. Eight red light emitting diodes (LED) were located on the left and right side of each camera (2 per camera) to excite the dye-loaded tissue (Figure 3A). After confirmation of dye-loading and prior to VF induction, the dissolved O_2 in the warm saline within the 25-liter tank was removed using nitrogen purging (N_2) via diffuser stones inside the tank. The latter aimed to simulate global ischaemia conditions associated with a VF-related cardiac arrest. When O_2 <5%, VF was induced by burst pacing on the epicardial ventricular surface with an electrical biphasic stimulator.[2] After VF onset, the coronary perfusion was stopped to further resemble global ischaemia conditions.

Transmembrane voltage changes during VF were captured with each of the 4 high-speed cameras (400 frames/sec) for at least 8 minutes (Figure 3A) without gaps during the entire VF episode. Since motion artefacts are minimal during VF, electromechanical uncouplers were not used in order to avoid altering the pathophysiology of myocardial ischaemia.[3]

Ex vivo mapping of transmembrane voltage changes using optical fibres and voltage ratiometry

During ventricular pacing with an electrical biphasic stimulator, the distal end of a single-core plastic optical fibre (2-3 mm diameter) was pressed gently against the surface of the contracting tissue. Blue (465-495 nm) and red (632-652 nm) excitation lights (blue LED: CBT-90-B; red LED: CBT-90-RX; Luminus Devices Inc., USA; AT480/30X passing 465—495 nm; ZET642/20X passing 632–652 nm; Chroma Technology Corp, USA) were used to generate fluorescence emission corresponding to numerator and denominator signals, respectively. Signals were then focused onto a custom-built silicon photodiode detection system, sampled (1 kHz sampling rate) and recorded using the PowerLab recording system (ADInstruments Inc., USA). The ratio of the numerator and the denominator signals permits the recording of optical action potentials with minimal artefacts during tissue contraction (Figure S17A). Recordings were acquired during ventricular pacing at different basic drive CLs (400, 300 and 250 ms) by positioning the plastic optical fibre against different epicardial ventricular regions: RV outflow tract, RV anterior wall, RV base posterior wall, RV mid posterior wall, LV base anterior wall, LV mid anterior wall, LV base posterior wall, LV mid posterior wall (Figure S17A).

In vivo analysis of metabolism-related parameters in blood samples from the coronary veins and coronary sinus

In anesthetized animals, hearts were exposed via median sternotomy, and 300 UI//Kg of unfractionated heparin (300 UI/kg) were administered before cannulation of specific coronary veins from the RV and LV. Coronary veins of similar calibre draining from the RV and the LV were cannulated using an intravenous line (24 G, 0.7x19 mm; BD Vialon, BD, USA). Blood samples (~100 µL) were drawn and analysed using the epoc® Blood Analysis System (Siemens Healthineers, Germany) according to the manufacturer's instructions. A blood sample from the coronary sinus was also taken using a 22G-needle (0.7x30 mm) and used as reference. Baseline samples were drawn during sinus rhythm before VF induction (Figure S19). Then, VF was induced using a 9 VDC battery and mechanical ventilation was stopped. Blood samples from RV and LV coronary veins were sequentially collected and analysed, aiming at drawing a minimum of 2 samples from each ventricle in the first 6 minutes of VF. Across animals, the first blood sample was alternated between RV and LV veins. After >6 minutes in VF, a blood sample from the coronary sinus was drawn again. Collection-time and time-to-analysis were annotated. For analysis, the measured parameters were assumed to follow a linear progression between samples.

In vivo measurement of cardiac NADH autofluorescence

In anesthetized animals, the antero-lateral walls of both ventricles were exposed via median sternotomy. An ultra-violet light emitting diode (peak emission at 365 nm) was used to homogeneously illuminate the exposed regions of the heart. Blue emitted fluorescence (440-480 nm) was imaged with a CMOS camera (IDS Imaging Development Systems GmbH, configured to record 200x240 super-pixels). An initial recording of 8 minutes, at 20 frames/sec, during sinus rhythm was used to assess the stability of NADH fluorescence levels under baseline conditions. Then, VF was induced using a 9 VDC battery and an 8-minute recording during VF was acquired. Mechanical ventilation was stopped upon VF induction. VF

Movies were processed with a spatial 5x5 median filter and a 21-frame temporal median filter. Filtering of optical signals was performed to mitigate fluctuations due to cardiac motion. Then, a region of interest (ROI) was delimited for each ventricle, with comparable sizes for both ventricles and similarly distanced from the centre of the image, aiming at displaying similar

baseline fluorescence. Fluorescence values from all pixels inside the ROI were averaged and the variations were normalized to the fluorescence at the frame after a 40-second blanking window following VF onset, to exclude signal artefacts associated with the sudden changes in heart size and position. The latter was done to avoid bias on the expected passive ventricular filling and dilation resulting from the cessation of effective myocardial contraction.[4] Nonetheless, ratiometry could not be performed to assess NADH autofluorescence *in vivo*. Therefore, signal artefacts from changes in heart size cannot be completely excluded.[4] However, we confirmed that NADH autofluorescence was stable during sinus rhythm, which indicates that the observed increase during VF is related to the ischaemia conditions of cardiac arrest. Figure S21A shows a sample frame of a post-filtered movie, along with the defined LV and RV ROIs. Figure S21B shows the comparison between the size of the LV and RV ROIs, with no statistically significant differences between both ventricles. Figure S21C shows the time-course of the NADH autofluorescence in the LV and the RV relative to the initial frame in an 8-minute movie during sinus rhythm, in which no NADH accumulation was observed in any case. Finally, Figure S21D shows the initial autofluorescence values during the VF episode measured in the LV and RV ROIs, with no significant differences between both ventricles at VF onset.

In vivo experimental data processing

Cardiac magnetic resonance imaging acquisition and processing

Animals with established myocardial infarction underwent 3D late-gadolinium enhancement cardiac magnetic resonance (LGE-CMR) imaging for characterization of the ischaemic substrate. Imaging was performed with an Achieva 3T-Tx whole-body scanner (Philips Healthcare, The Netherlands) equipped with a 32-element and phased-array cardiac coil. Seven minutes after intravenous contrast injection of 0.2 mmol/kg gadoteric acid (Dotarem, France) 3D LGE-CMR sequences were acquired using an inversion-recovery spoiled turbo field echo (IR-T1TFE) with isotropic resolution of 1.5x1.5x1.5 mm. LGE-CMR images were then processed using the ADAS3D software (v.2.11.0, Adas3D Medical S.L., Spain) for scar identification based on the full-width-half-maximum method, which normalizes signal intensity to maximum myocardial signal intensity.[1] More specifically, signal intensity cut-off values of 0.45 and 0.67 were used for detecting heterogenous and dense scar, respectively.[1]

Since the ADAS3D software is not optimized for RV segmentation, segmentations of the LV alone and of both ventricles together were performed. After file exportation, the epicardial layer (i.e., layer 90%) of the LV segmentation was subtracted from the endocardial layer (i.e., layer 10%) of the biventricular geometry to obtain an endocardial RV geometry (Figure S1). These steps were performed using ParaView software (v5.6.1, Kitware Inc., USA), MeshLab software (v.2016.12, ISTI-CNR, Italy), and custom-made software in Matlab (MathWorks Inc., USA).

Catheter location and projection onto LGE-CMR geometries

In animals undergoing *in vivo* invasive electrophysiological studies, orthogonal fluoroscopic images (antero-posterior and left-lateral views) were acquired during the procedure. X-ray images were recaptured after any DC shock to assess catheter location stability. When multiple 24-pole catheters were advanced into the RV or the epicardium, successive images were acquired after positioning each catheter to ensure a correct identification of each catheter electrodes. Images were then processed off-line with custom-made software in Matlab. Catheter electrodes were identified and tagged, and the heart contour was delineated in the fluoroscopic images. For the basket catheter, each spline (A, B, C, …, H) was identified based on a specific marker based on the position of a wider electrode within the spline, which is visible in X-ray images. Electrodes from 24-pole catheters were identified straightforward starting from the distal tip. LGE-CMR geometries (endo-LV, endo-RV and epicardium) were registered onto the fluoroscopic images using their contours in the corresponding orthogonal views. Electrodes displaying atrial and ventricular electrograms with similar amplitude guided the delineation of annular regions. Electrodes were then projected onto their corresponding geometry using a minimum-total-distance criterion (adding distances from both views). Electrodes displaying

poor contact with overt far field signal were discarded and those projected in an inconsistent manner (i.e., not following the catheter shape) were manually modified. Electrodes projected into LGE-CMR-derived scar regions were identified and confirmed by the presence of fragmented potentials. A schematic is shown in Figure S2A.

Finally, 5 regions were defined for the endocardium of the LV: apex, posterior wall, free wall, septal wall and anterior wall. Each electrode from the basket catheter located in the LV was manually assigned to one of the regions, based on their location on the LV endocardial mesh. Similarly, 4 regions were defined for the endocardium of the RV: apex, free wall, septal wall and outflow tract. Each electrode from the catheters located in the RV was assigned to one region of the RV endocardial mesh. A 2D bird's-eye schematic representation of the 9 endocardial regions was used to easily visualize and summarize the analysed data. A sample cases is shown in Figure S9, where the average VF-leading score from the electrodes in each endocardial region is color-coded into the 2D schematic. This allows for an inter-case reproducible representation of the ventricles.

The position of epicardial multipolar catheters was not reproducible among cases, which did not enable us to assign electrodes to specific epicardial subregions. Therefore, catheter electrodes placed on the epicardium were visually assigned to the LV or the RV. Electrodes close to interventricular grooves (both anterior and posterior) were discarded to minimize the effect of potential errors in the projection process assigning electrodes on these interventricular regions to the wrong ventricle (Figure S2B).

Computation of activation-recovery intervals

The unipolar recordings from the mapping/ablation catheter and the surface ECG during the restitution protocol were acquired with the Labsystem PRO EP recording system (Boston Scientific, USA). The recording was divided into segments for each pacing CL and each ventricle, and processed off-line using custom-made software in Matlab. The unipolar signal was filtered with a temporal 10th-order median filter. The paced beats of interest were identified using an ECG lead and were manually annotated. Activation time was measured in the unipolar signal as the time of maximum negative slope, and the recovery time as the moment of maximum positive slope of the repolarization wave.[5] The ARI was calculated as the difference between these two times (Figure 4A and Figure S16). During pacing at the basic drive CL, multiple beats were averaged to obtain a mean ARI, whilst only the coupled beat (S2) was analysed during the S1-S2 protocol at each pacing rate.

Computation of time-domain instantaneous frequency modulation analysis of bipolar electrograms

The instantaneous frequency modulation (iFM)[6] values of ventricular endocardial and epicardial electrograms were used to study temporal evolution of VF dynamics. Importantly, the iFM computation described by Quintanilla *et al.* was designed and optimized for unipolar recordings of atrial signals during atrial fibrillation.[6] In the ventricles and during VF, we decided to use bipolar recordings, given their reduced susceptibility to noise (mostly common to both unipoles and, therefore, minimized when subtracting the two unipolar signals used to generate the bipolar signal) and, more importantly, because of their superior ability to distinguish low-amplitude local activity within infarcted regions from high-amplitude far-field activity originating from remote healthy tissue. Therefore, here we used a similar iterative method as the one reported by Ng *et al.* to detect local activations at the times of maximum absolute amplitudes in bipolar recordings.[7] Then, an iFM analysis of these local activations was performed to accurately estimate ventricular activation rates during VF. For this purpose, VF episodes were divided into 5-second windows with a 50% window-overlap to yield an accurate estimate of ventricular activation rates every 2.5 seconds. Figure S3 depicts the complete process for the iterative annotation of bipolar local activations using iFM analysis. The medians of the resulting iFM signals were extracted to provide accurate estimates of ventricular activation rates. Examples of bipolar signals and the resulting iFM analysis during VF are shown in Figure S4 and Figure S5.

Figure S4 and Figure S5 also show the comparison between the activation rates estimated with iFM_{median} of bipolar signals versus the more conventional frequency-domain estimator (i.e., Dominant Frequency [DF]).

VF-leading score analysis

We defined a VF-leading score that could detect regions potentially leading the fibrillatory process in terms of their higher hierarchy in activation rates during short VF episodes. The VF-leading score aimed to identify the propensity of a given endocardial region to host the highest activation rates. More specifically, for a given 30-second VF episode, iFM_{median} was calculated for each endocardial bipole and in each 5-second window with a 50% time overlap (i.e., 11 time-windows: [0-5] s, [2.5-7-5] s, [5-10] s, …, [22.5-27.5] s, [25-30] s). For every window, values from both ventricles were normalized using their percentiles so as the bipole with highest iFM_{median} (i.e., fastest activation rate) had a percentile (i.e., score) of 100 and the bipole with the lowest iFM_{median} had a score of 0. The average percentile/score of each electrode along the 11 time-windows was referred to as the VF-leading score, yielding a value ranging from 0 to 100. A value of 100 would correspond to a bipole with the highest iFM_{median} during all the 11 temporal windows of a 30-second VF episode. Conversely, a value of 0 would correspond to a bipole with the lowest iFM_{median} during all the 11 temporal windows of a 30-second VF episode. Finally, bipoles inside each endocardial region were averaged and represented in the 2D schematic, as explained above. Thus, even though local activation rates may vary during the episode, the VF-leading score offers a summary of the activation rate hierarchies present in the endocardium during early VF and identifies high hierarchy regions that might be potentially responsible for the early maintenance of VF. A sample case is depicted in Figure S6.

Computation of a frequency-domain estimator of activation rates in surface ECG tracings

Annotation of local activation times in surface ECG makes no sense since it does not reflect local but global activation. Therefore, time-domain approaches like the iFM analysis were discarded for this purpose. DF analysis seemed also potentially challenging as the ECG reflects the electrical activity of a large portion of the myocardium, with a wide range of activation rates observed in the pig, which often yields a multi-component spectrum with multiple peaks of similar amplitude. The latter makes annotation of the DF challenging. Therefore, the median frequency (F_{median}) was selected as an activation-rate estimator for surface ECG tracings. F_{median} is defined as the frequency value that divides the power spectral density (PSD) into two subranges with equal total power.[8] In pigs, the physiological range of frequencies for VF was 3.75-20 Hz.[9] The PSD was estimated by a conventional periodogram of 5-second signals previously multiplied by a Kaiser window (β=2) and zero padded to the next higher power of 2.

In both pigs and humans, lead II was used as an estimator of the global electrical activity of the heart during VF. This lead was also chosen because it is commonly available in single-lead out-of-hospital VF traces. In pigs, leads V1 and V4 were used as estimators of the electrical activity of the RV and LV, respectively. Figure S7 shows a sample pig case with the spatial location of the six precordial leads over the LV and RV geometries acquired with the electroanatomical mapping system (Carto3, Biosense Webster, Haifa, Israel). In Figure S7, V1 and V4 unipolar leads mostly cover RV and LV activity, respectively. Sample V1 and V4 ECG traces during VF and their F_{median} analysis are also shown.

Ex vivo experimental data processing

Assignment of pixels to epicardial regions

The epicardium of the RV and the LV was divided into thirteen regions (Figure 3B). Each ventricle was divided into posterior, free and anterior walls, and in mid and base segments. Additionally, the LV apex was independently defined. Using custom Matlab software, region boundaries were delineated for each of the 4 field-of-views (i.e., cameras). Pixels inside each

region were identified. Pixels within regions visualized from 2 cameras were analysed in the images of the camera that provided the less lateral distortion.

Computation of time-domain iFM analysis

Optical movies (120x160 pixels) were divided into 5-second windows (400 frames/sec x 5 seconds = 2000 frames) with 50% temporal overlap. A drift removal filter was applied in combination with a low pass spatiotemporal filter with a conical-shaped kernel.[6] The algorithm used to calculate iFM in optical signals was replicated from Quintanilla *et al.*[6] The analysis included the *iFM(t)* of individual optical signals at each pixel location and calculation of their median (iFM_{median}). Sample optical signals and their corresponding iFM analysis are shown in Figure 3B, Figure S13 and Figure S14.

Signal processing of ratiometric signals of transmembrane voltage changes

The ratiometric signal was processed offline with a custom-made software tool in Matlab. A drift removal filter was applied (2nd-order, 4-Hz high-pass filter) to the signal in combination with a temporal 5th-order median filter. For each ventricular region and at each pacing rate, a minimum of 3 action potentials were selected. Then, the activation time of each action potential was calculated at the time of maximum positive slope $(dV/dt)_{max}$. The APD was calculated as the difference between the activation time and the time to 90% repolarization (APD_{90}). The optical action potential upstroke duration, reflecting the depolarization phase, was calculated as the time interval between 10% and 90% depolarization. Due to the spatial integration of the fluorescence registered from the optical fibre tip that collects light emissions from multiple cardiomyocytes, optical upstrokes can be an order of magnitude longer than single-cardiomyocyte upstrokes measured electrically using microelectrodes.[10] Finally, the APD_{90} and upstroke durations of the annotated beats for each region and pacing rate were averaged to obtain a single value per animal and region. Sample optical action potentials are shown in Figure 4B and Figure S17B.

In vitro experimental studies

Protein and gene expressions of the ATP-sensitive potassium channel

In anesthetized animals, hearts were exposed via median sternotomy. Epicardial biopsies from the anterior wall of the RV and LV were rapidly taken during sinus rhythm using a scalpel, and immediately snap-frozen in liquid nitrogen to prevent protein degradation. Tissue samples were then processed following specific protocols to measure gene and protein expressions. K_{ATP} channels are hetero-octameric complexes conformed by 4 pore-forming inward rectifying subunits (K_{ir}6.x gene family) and 4 regulatory sulfonylurea-receptor subunits (SURx gene family).[11] Both subunits have gene variants (K_{ir}6.1 and K_{ir}6.2; SUR1, SUR2A and SUR2B), although scarce data are available on the prevalent subunits assembling K_{ATP} channels in ventricular cardiomyocytes from pigs. Therefore, the analyses included gene and protein expressions of all 5 subunits.

<u>Real time quantitative polymerase chain reaction (RT-qPCR)</u>

Gene expression analysis was performed by RT-qPCR. Total RNA was isolated from the epicardial frozen samples using TRIzol Reagent (Thermo Fisher Scientific, USA) following the standard protocol. Reverse transcription was processed using the Applied Biosystems High-Capacity cDNA Reverse Transcription kit (Thermo Fisher Scientific). RT-qPCR was conducted with SYBR Green (Thermo Fisher Scientific), where cDNA was amplified by using custom DNA primers based on the pig genome (Table S4). GAPDH was used as housekeeping gene for gene expression normalization. Samples with <35 C_t were selected and the $2^{-\Delta\Delta Ct}$ method was used for relative gene expression quantification.[12]

Western blotting

Proteins were extracted from the epicardial frozen samples with Radio-Immunoprecipitation Assay (RIPA) buffer supplemented with complete protease inhibitor cocktail (Roche-Diagnostics, Switzerland) and PhosSTOP phosphatase inhibitor table (Roche-Diagnostics) according to the manufacturers' instructions. Total protein concentration was quantified with the Pierce bicinchoninic acid (BCA) protein assay kit (Thermo Fisher Scientific). Twelve mg of total protein was resolved by Sodium dodecyl-sulphate polyacrylamide gel electrophoresis (SDS-PAGE) and transferred to nitrocellulose membranes (Bio-Rad Laboratories, Hercules, CA). The MemCode Reversible Protein Stain kit (Thermo Fisher Scientific) was used to visualize total protein levels. Membranes were then washed with 0.2% Tween Tris-buffered saline solution and blocked with 5% bovine serum albumin (BSA) for 1 h. Afterwards, primary antibodies were incubated overnight at 4°C. Membranes were then washed and incubated with the corresponding horseradish peroxidase (HRP)-conjugated secondary antibody (Table S5). Total protein staining and the protein bands (enhanced by chemiluminescence with Immobilon Forte Western HRP substrate [Merck Millipore, USA]) were visualized in the iBright™ FL1500 Imaging System (Thermo Fisher Scientific). The iBright™ Analysis Software was then used to perform protein analysis. Band proteins were normalized to the total protein quantification of each sample.

Cell isolation of ventricular cardiomyocytes and patch-clamping

The procedure for cardiomyocyte isolation was adapted from Macías A *et al.*[13] In anesthetized animals, the hearts were exposed and excised as described in previous sections and embedded on ice-cold saline solution. Within a 5-minute window after euthanasia and heart excision, the aorta was cannulated and perfused with 4x50 mL flushes of ice-cold saline solution to remove blood from the coronary arteries and a final one with calcium-free Tyrode solution (CFTS; in mM: NaCl 120, KCl 5.4, KH_2PO_4 1.2, $MgCl_2$ 1, HEPES 10, Pyruvate 5, Taurine 10, and Glucose 20; pH 7.4 with NaOH) to stop contractile activity. Then, the anterior part of the heart including the LAD, was placed into a bath and the LAD was cannulated with a custom-made 18G cannula and perfused through a Langendorff perfusion system with the CFTS at 37ºC for 15 minutes at 4 mL/min. Then, the ventricular tissue was separated into RV and LV and chopped into chunks of ≈1-2 mm^3 with scissors. Chunks were stirred for 12 min in CFTS solution at 37°C, oxygenated with carbogen (O_2-CO_2 95/5%), and softly shaken in a baker. Every 3 minutes, the tissue was transferred to a fresh solution. Tissue was enzymatically digested with digestion-buffer (DB: CFTS supplemented with 0.4 mg/mL Liberase™, 5.5 mmol/L trypsin 2.5% and 12.5 μM $CaCl_2$) for 15-20 minutes at 37°C. Every 5 minutes, the supernatant was visualized under a microscope and chunks transferred to fresh and warm DB. When rod-shape cardiomyocytes were visualized in the supernatant, an additional 5-minute digestion step along with a slight mechanical dissociation were applied to the chunks. Thus, the supernatant containing myocytes was supplemented with 10% v/v FBS to stop the enzymatic digestion and centrifuged for 3 min at 100xg at room temperature. Finally, cardiomyocytes were reloaded with Ca^{2+} by incubation in 10 min-each $CaCl_2$ concentrations (0.112 and 1 mmol/L). Cardiomyocytes were stored in that solution at room temperature until used.

Whole-cell voltage-clamp recordings and data analysis procedures were similar to those reported elsewhere.[13] Cells were placed in a perfusion chamber (RC-26, Warner Instruments, CT) mounted on the stage of an inverted microscope (DMi8, Leica, Germany). Cells were allowed to settle on the bottom of the perfusion chamber before perfusion with a normal Tyrode solution (in mM: NaCl 137, KCl 5.4, $CaCl_2$ 1.8, $MgCl_2$ 0.5, glucose 10, and HEPES 11.8, pH = 7.35 with NaOH). Pipettes made from borosilicate glass (GD-1, Narishige, UK, OD: 1 mm; ID: 0.6 mm) had resistances of 1-3 MΩ when filled with the internal pipette solution, containing (mM): KCl 20, K-aspartate 120, NaCl 10, MgATP 5, HEPES 10, pH = 7.2 with KOH. For $I_{K\text{-}ATP}$ recordings, 500 ms voltage-clamp steps were applied in 10 mV increments from –140 to +20 mV with a –40 mV holding potential, every 5 seconds. ATP-sensitive K^+ current (I_{KATP}) was calculated by subtracting currents recorded in the absence or presence of pinacidil 100 μM.

Computational simulations of myocardial ischaemia and ventricular fibrillation

The experimental results obtained were incorporated into a ventricular myocyte model to create different computational models for LV and RV myocytes and simulate their behaviour during global ischaemia conditions. Action potentials and underlying ionic currents were simulated using the model described by O'Hara *et al.*[14] The model was modified as described by Ferrero *et al*. to simulate acute myocardial ischaemia and incorporate equations to simulate the effects of I_{KATP}.[15] The computer model incorporated the experimental data from RV and LV on the following parameters: *i)* Baseline APD measurements in the absence of ischaemia; *ii)* Whole-cell voltage clamping data (total K^+ inward current, I_{KATP} and cell capacitance); and *iii)* Metabolic parameters (pH and $[K^+]_o$) from blood samples of RV and LV coronary-veins during *in vivo* VF with known electrophysiological effects on APD and excitability. Of note, APD values were not explicitly set in the model, as APD is an output of the O'Hara model. Instead, APD measured experimentally, and total K^+ inward and I_{KATP} currents measured in whole-cell voltage clamping were used to guide the assumption of equal RV-LV conductance of other inward potassium currents (I_{Kr}, I_{Ks} and I_{K1}) that were not measured experimentally and were imposed in the model. Similarly, sodium conductance was not measured experimentally but was set identical for both ventricles given the similar upstroke duration of the action potential observed in *ex vivo* action potentials measured with ratiometric optical mapping (Figure S17D).

First, single isolated cell action potentials during progressive acute ischaemia were simulated by pacing the cell at different constant basic drive CLs ranging from 2000 ms to 200 ms, applying rectangular pulses of 0.5 ms in duration and twice the diastolic threshold in amplitude (Figure 6B). Dynamic restitution curves were obtained for both normoxic and ischaemic conditions, incorporating the experimental data of pH and $[K^+]_o$. To properly simulate progressive ischaemia, time courses of intracellular ATP, ADP and lysophosphatidylcholine (LPC) were also imposed to the model as described elsewhere.[15] Simulations were carried out in Matlab.

Then, tissue simulations were carried out by defining two adjacent 5x5 cm^2 squares of monodomain virtual anisotropic tissue, with LV and RV models under ischaemic conditions. Tissue conductivity was adjusted in order to obtain a longitudinal conduction velocity of 0.7 m/s and a transversal conduction velocity of 0.25 m/s. The tissue was stimulated in the lower side with a constant basic drive CL of 800 ms until reaching a steady-state. Then, re-entrant activity was induced by cross-shock stimulation applied during the vulnerable window. Cross-shock stimulation was performed by delivering a premature stimulus in the lower left quarter of the tissue. Simulations were carried out in the Mare Nostrum 5 super computer of the Barcelona Supercomputing Centre (BSC) using the Elvira software.[16]

Virtual 5-mm electrodes were evenly located 1 mm above the virtual tissue to simulate cavitary bipolar recordings comparable to those obtained *in vivo* (Figure 6C). iFM_{median} was calculated in each bipole for 1 second of the simulated fibrillatory activity and resulting iFM_{median} maps were computed for the LV and RV tissue (9x9 bipoles for each ventricle, Figure 6D).

Clinical study in patients with cardiac arrest events associated with ventricular fibrillation

VF episodes with >2 minutes of continuous ECG telemetry before defibrillation were used to analyse the time-course of ventricular ARs in 3 patients with in-hospital cardiac arrest (Group 1, Figure S8A). The ECG traces of the episode were divided into 5-second segments with 50% temporal overlap. These 5-second VF segments were digitized using a supervised semi-automatic custom tool in Matlab. Briefly, ECG tracings on paper were scanned to a digital image and VF tracings were identified and extracted using image-processing techniques. Resulting digitized VF ECG signals had a temporal resolution equivalent to approximately 1 kHz sampling rate and were band-pass filtered between 2 Hz and 200 Hz. After PSD calculation, F_{median} was used to estimate the VF activation rate, as described above. In this case, the spectral band of interest was set to 2-10 Hz, based on the physiological activation-rate range typically observed during VF episodes in humans (Figure S8B). VF segments during

cardiopulmonary resuscitation were excluded from the analysis due to the artefacts caused by chest compressions, which made spectral analysis unfeasible. The series was conducted at Hospital Clínico San Carlos, Madrid.

A second retrospective series of patients admitted to hospital in comatose status (Glasgow Coma Scale ≤8) after a VF-related cardiac arrest event, and eventual return of spontaneous circulation before admission, was used to study the clinical value of VF activation rates to predict neurological performance at hospital discharge (Group 2, Figure S8A). Spectral analysis of the last seconds of VF before the first DC shock (≥1 second, up to 7 seconds) was performed when ECG traces displayed enough quality and duration for digitization and analysis (Figure S8B). Patients were classified as favourable or non-favourable neurological performance (FNP and non-FNP, respectively) using the Pittsburgh outcome categorization of brain injury.[17] Cerebral performance categories (CPCs) 1 and 2 (good and moderate disability, respectively) were considered as FNP, and CPCs 3, 4 and 5 (severe disability, vegetative state and brain death, respectively) were considered as non-FNP. Table S2 and Table S3 show the clinical and cardiac-arrest characteristics of all patients and their association with the neurological outcome. The series was conducted at the Hospital Universitario La Paz, Madrid. Exclusion criteria for Group 2 patients were age <18 years, non-shockable or shockable rhythms other than VF, a terminal illness or cognitive deterioration prior to the cardiac arrest, possible causes of coma other than cardiac arrest, early mortality or hemodynamic instability leading to incomplete or absence of subsequent withdrawal of sedation to assess cerebral performance.

Retrospective data from all patients were obtained from clinical records during hospitalization. The institutional ethics review committees of both institutions approved the study and data analyses, in accordance with the ethical guidelines of the Declaration of Helsinki and European guidelines for good clinical practice.

Additional details of statistical analysis

A two-way repeated measures ANOVA test was used to compare evolution of RV and LV parameters, or endocardial and epicardial parameters over the time-course of VF episodes, or at different pacing rates, coupling intervals or voltage amplitudes. A significant interaction indicates that the effect of one independent variable (e.g. time) on the dependent variable (e.g. AR) depends on the level of the other independent variable (e.g. RV or LV).

REFERENCES

1. López-Yunta M, León DG, Alfonso-Almazán JM, Marina-Breysse M, Quintanilla JG, Sánchez-González J, Galán-Arriola C, Cañadas-Godoy V, Enríquez-Vázquez D, Torres C, Ibáñez B, Pérez-Villacastín J, Pérez-Castellano N, Jalife J, Vázquez M, Aguado-Sierra J, Filgueiras-Rama D. Implications of bipolar voltage mapping and magnetic resonance imaging resolution in biventricular scar characterization after myocardial infarction. *Europace* 2019;**21**:163–174.

2. Lee P, Calvo CJ, Alfonso-Almazán JM, Quintanilla JG, Chorro FJ, Yan P, Loew LM, Filgueiras-Rama D, Millet J. Low-Cost Optical Mapping Systems for Panoramic Imaging of Complex Arrhythmias and Drug-Action in Translational Heart Models. *Sci Rep 2017 7:1* 2017;**7**:1–14.

3. Venable PW, Sciuto KJ, Warren M, Taylor TG, Garg V, Shibayama J, Zaitsev A V. Mitochondrial depolarization and asystole in the globally ischemic rabbit heart: Coordinated response to interventions affecting energy balance. *Am J Physiol Heart Circ Physiol* 2015;**308**:H485–H499.

4. Wengrowski AM, Kuzmiak-Glancy S, Jaimes R, Kay MW. NADH changes during hypoxia, ischemia, and increased work differ between isolated heart preparations. *Am J Physiol Heart Circ Physiol* 2013;**306**:H529.

5. Cluitmans MJM, Bear LR, Nguyên UC, Rees B van, Stoks J, Bekke RMA Ter, Mihl C, Heijman J, Lau KD, Vigmond E, Bayer J, Belterman CNW, Abell E, Labrousse L, Rogier J, Bernus O, Haïssaguerre M, Hassink RJ, Dubois R, Coronel R, Volders PGA. Noninvasive detection of spatiotemporal activation-repolarization interactions that prime idiopathic ventricular fibrillation. *Sci Transl Med* 2021;**13**:eabi9317.

6. Quintanilla JG, Alfonso-Almazán JM, Pérez-Castellano N, Pandit S V., Jalife J, Pérez-Villacastín J, Filgueiras-Rama D. Instantaneous Amplitude and Frequency Modulations Detect the Footprint of Rotational Activity and Reveal Stable Driver Regions as Targets for Persistent Atrial Fibrillation Ablation. *Circ Res* 2019;**125**:609–627.

7. Ng J, Sehgal V, Ng JK, Gordon D, Goldberger JJ. Iterative method to detect atrial activations and measure cycle length from electrograms during atrial fibrillation. *IEEE Trans Biomed Eng* 2014;**61**:273–278.

8. Huizar JF, Warren MD, Shvedko AG, Kalifa J, Moreno J, Mironov S, Jalife J, Zaitsev A V. Three distinct phases of VF during global ischemia in the isolated blood-perfused pig heart. *Am J Physiol Heart Circ Physiol* 2007;**293**:H1617–H1628.

9. Quintanilla JG, Moreno J, Archondo T, Chin A, Pérez-Castellano N, Usandizaga E, García-Torrent MJ, Molina-Morúa R, González P, Rodríguez-Bobada C, Macaya C, Pérez-Villacastín J. KATP channel opening accelerates and stabilizes rotors in a swine heart model of ventricular fibrillation. *Cardiovasc Res* 2013;**99**:576–585.

10. Girouard SD, Laurita KR, Rosenbaum DS. Unique properties of cardiac action potentials recorded with voltage-sensitive dyes. *J Cardiovasc Electrophysiol* 1996;**7**:1024–1038.

11. Foster MN, Coetzee WA. KATP channels in the cardiovascular system. *Physiol Rev*

12. Livak KJ, Schmittgen TD. Analysis of relative gene expression data using real-time quantitative PCR and the 2(-Delta Delta C(T)) Method. *Methods* 2001;**25**:402–408.

13. Macías Á, González-Guerra A, Moreno-Manuel AI, Cruz FM, Gutiérrez LK, García-Quintáns N, Roche-Molina M, Bermúdez-Jiménez F, Andrés V, Vera-Pedrosa ML, Martínez-Carrascoso I, Bernal JA, Jalife J. Kir2.1 dysfunction at the sarcolemma and the sarcoplasmic reticulum causes arrhythmias in a mouse model of Andersen-Tawil syndrome type 1. *Nat Cardiovasc Res* 2022;**1**:900–917.

14. O’Hara T, Virág L, Varró A, Rudy Y. Simulation of the undiseased human cardiac ventricular action potential: model formulation and experimental validation. *PLoS Comput Biol* 2011;**7**:e1002061.

15. Ferrero JM, Gonzalez-Ascaso A, Matas JFR. The mechanisms of potassium loss in acute myocardial ischemia: New insights from computational simulations. *Front Physiol* 2023;**14**:1074160.

16. Heidenreich EA, Ferrero JM, Doblaré M, Rodríguez JF. Adaptive macro finite elements for the numerical solution of monodomain equations in cardiac electrophysiology. *Ann Biomed Eng* 2010;**38**:2331–2345.

17. Jennett B, Bond M. Assessment of outcome after brain damage: a practical scale. *The Lancet* 1975;**305**:480–484.
