# Supplementary material for: Ventricular fibrillation dynamics reveal regional asymmetry in resilience to cardiac arrest and predict clinical outcome
Source: Cardiovasc Res. 2026 May 28;122(9):1191–205. doi: 10.1093/cvr/cvag101 (PMC13307562; doi:10.1093/cvr/cvag101)
Supplement: cvag101_Supplementary_Data [file cvag101_supplementary_data.zip › Unprocessed blots final.pdf]

Kir6.1

The results obtained from the analysis of these Western Blots are shown in Figure 4G

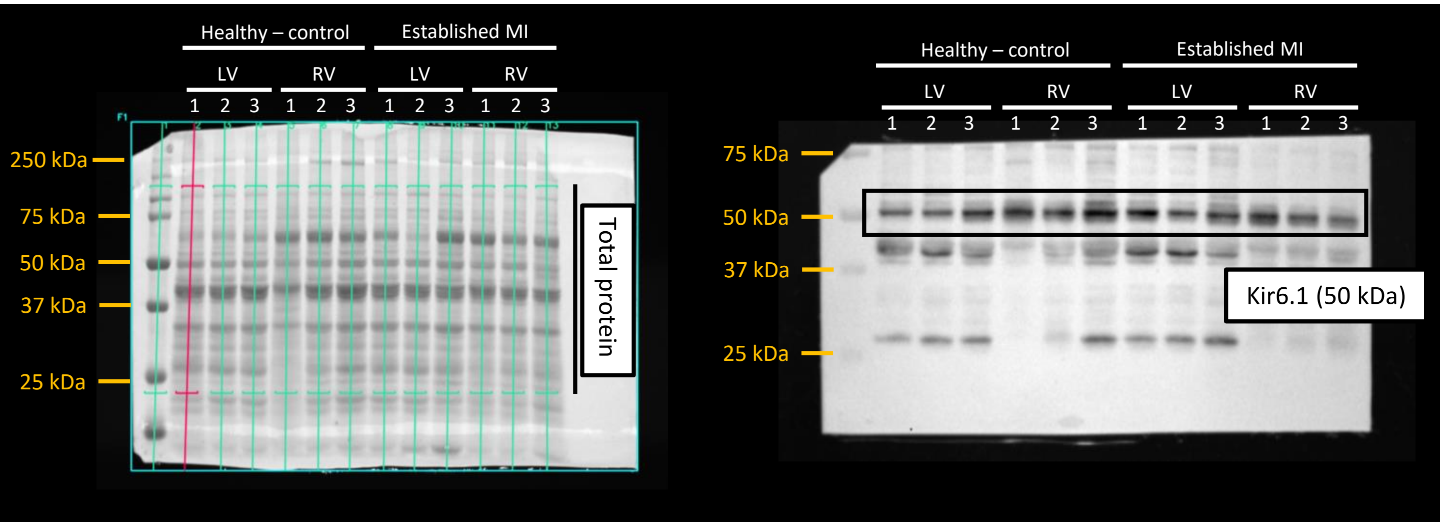

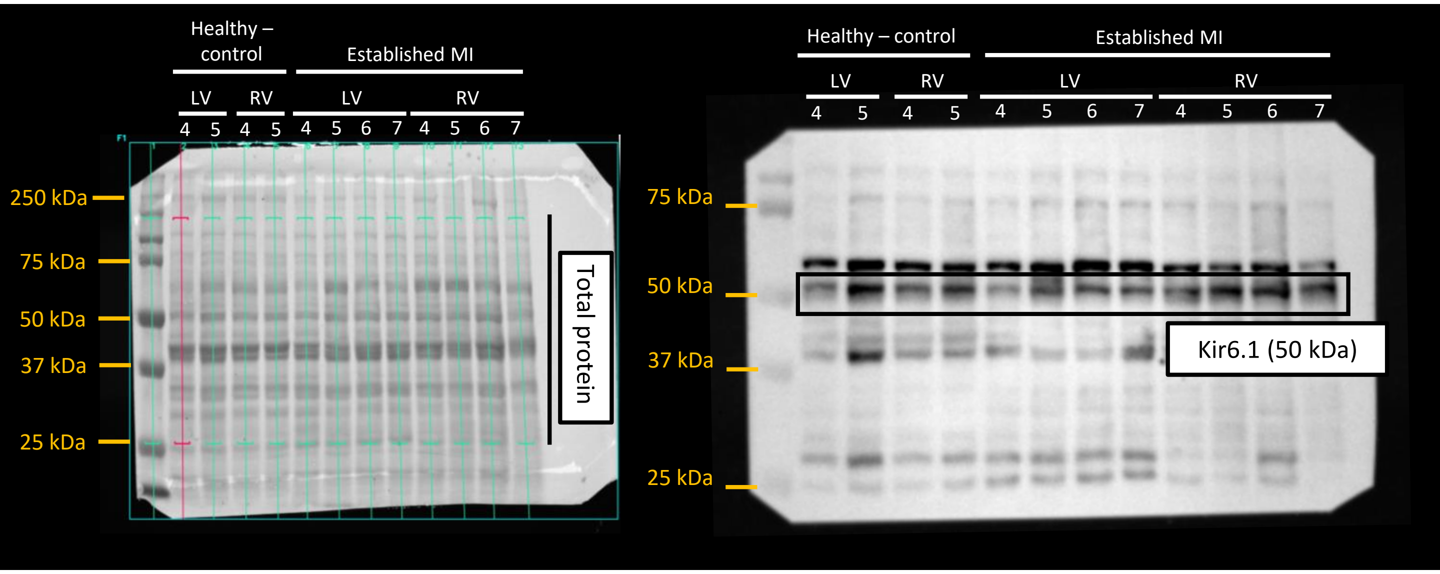

LV: left ventricle
MI: myocardial infarction
RV: right ventricle

SUR2B

The results obtained from the analysis of these Western Blots are shown in Figure 4G

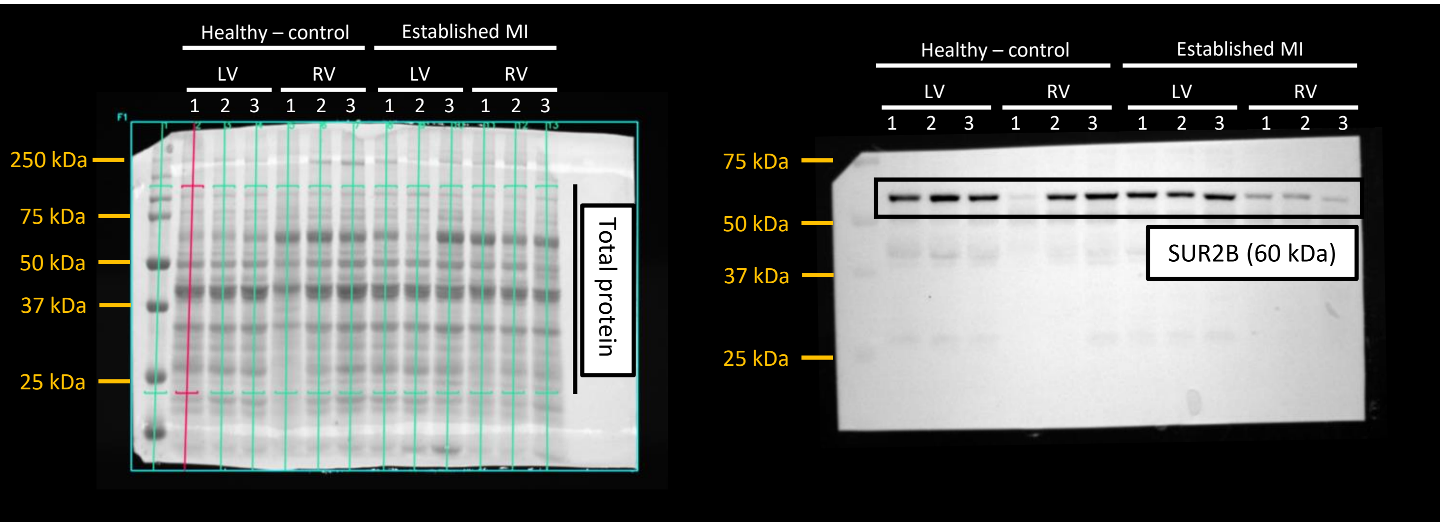

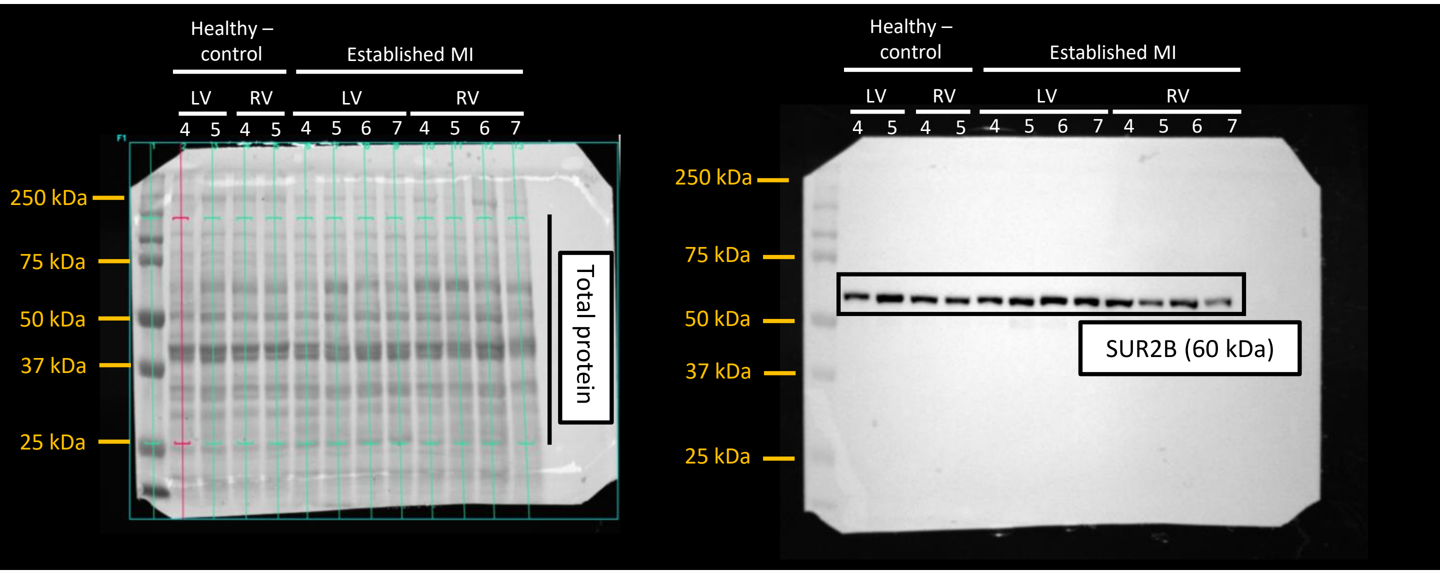

LV: left ventricle
MI: myocardial infarction
RV: right ventricle

Kir6.2

The results obtained from the analysis of these Western Blots are shown in Suppl. Figure 18C

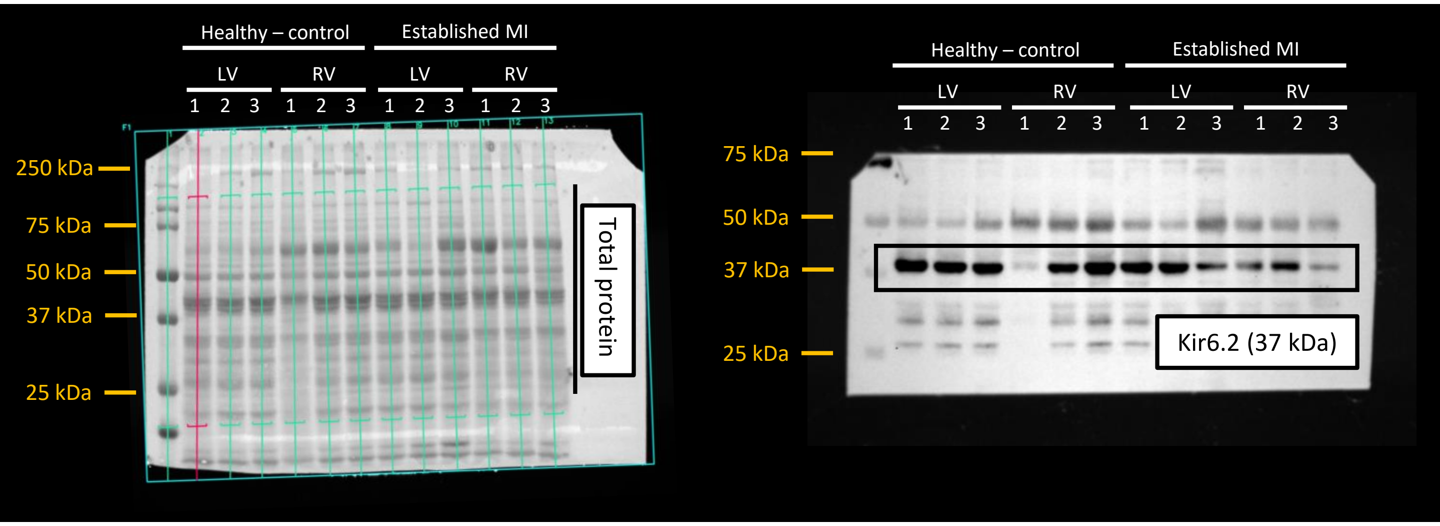

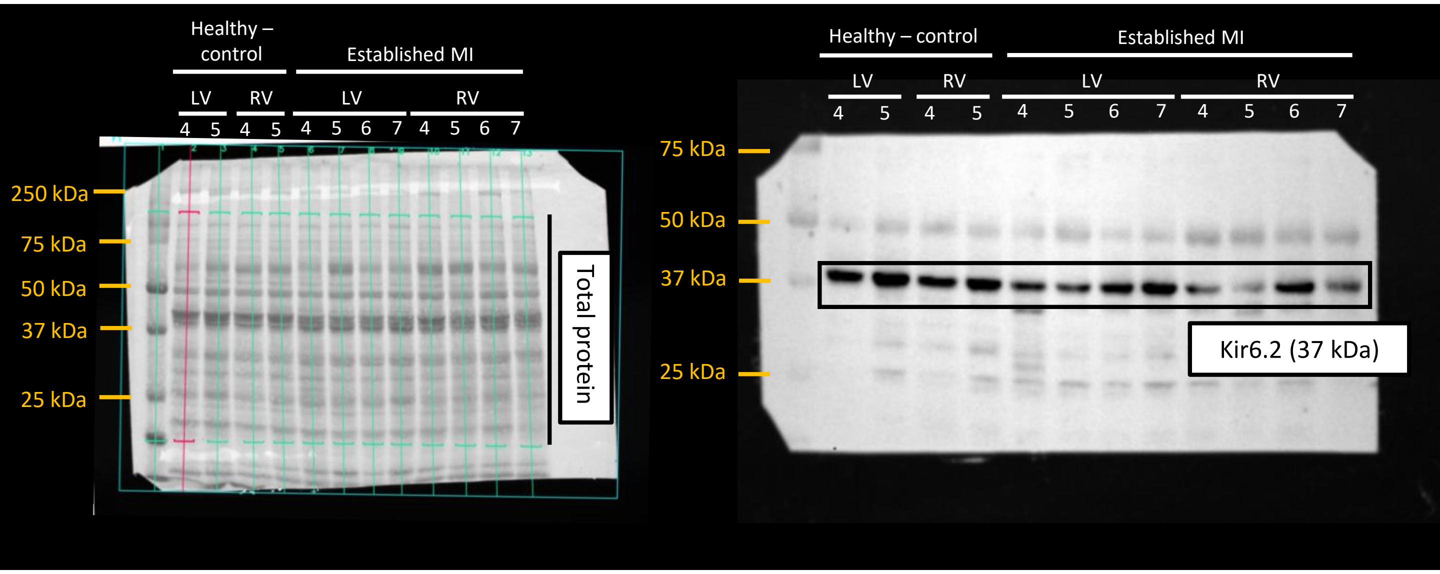

LV: left ventricle
MI: myocardial infarction
RV: right ventricle

SUR2A

The results obtained from the analysis of these Western Blots are shown in Suppl. Figure 18C

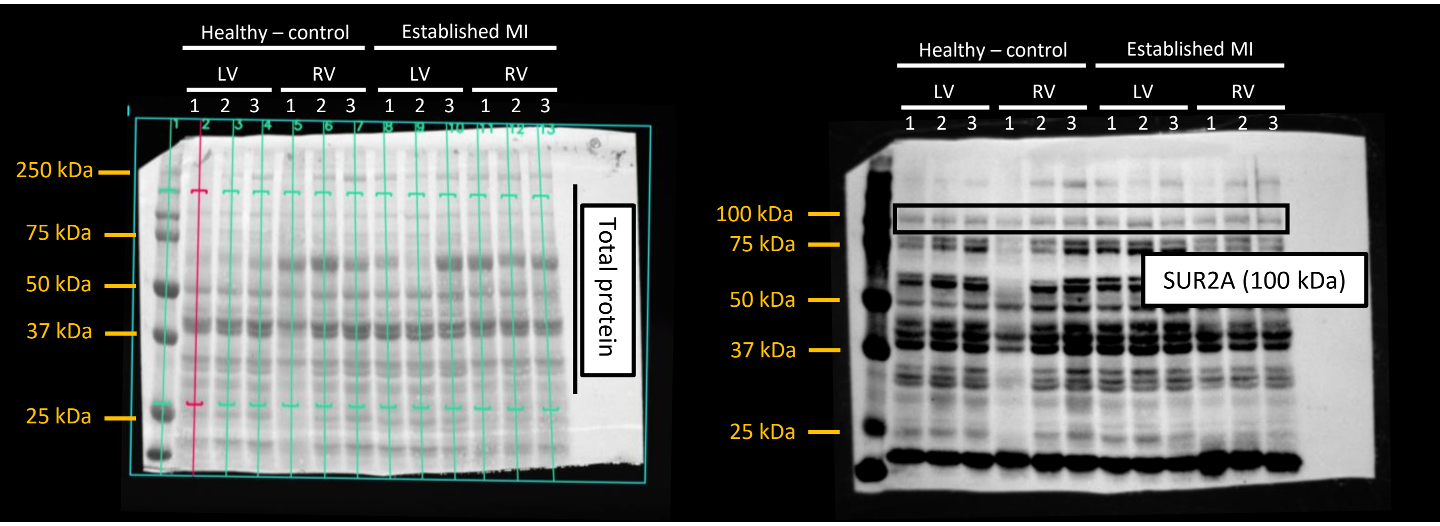

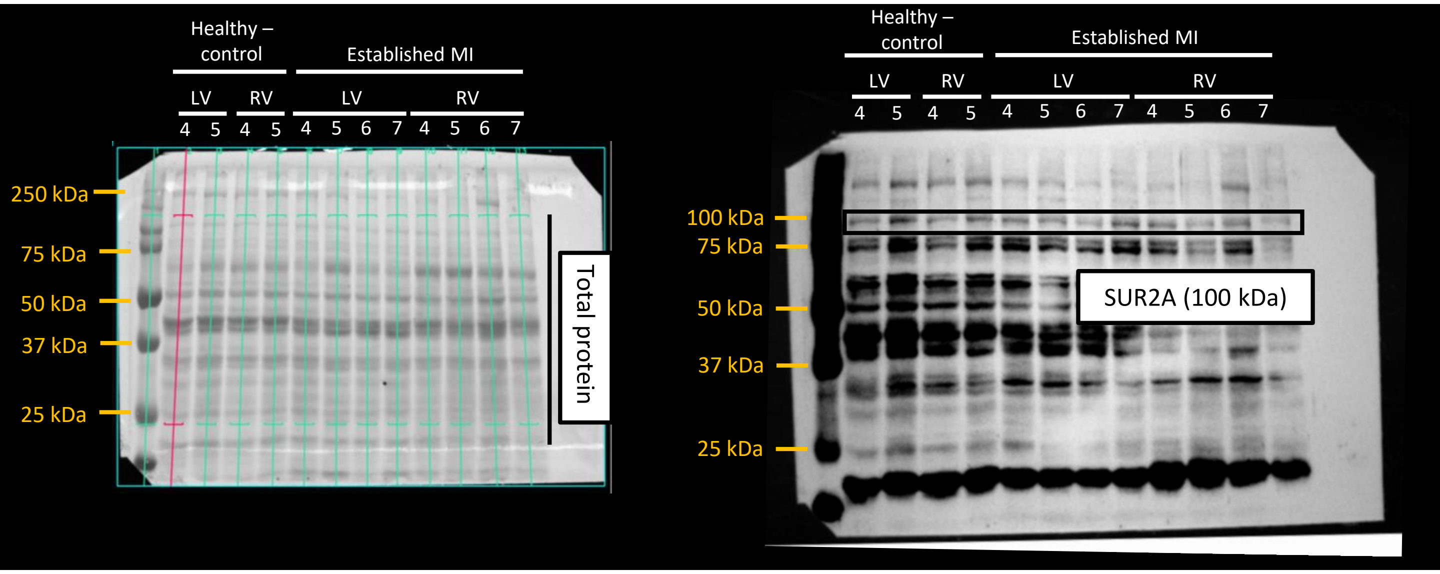

LV: left ventricle
MI: myocardial infarction
RV: right ventricle
